# Unsymmetrical difunctionalization of cyclooctadiene under continuous flow conditions: expanding the scope of ring opening metathesis polymerization†
†Electronic supplementary information (ESI) available: General information concerning experimental procedures, and characterization data including NMR and IR spectra of isolated monomers and NMR, IR, GPC, DSC and TGA spectra of isolated polymers are available. CCDC 1562950. For ESI and crystallographic data in CIF or other electronic format see DOI: 10.1039/c7sc04580h


**DOI:** 10.1039/c7sc04580h

**Published:** 2018-01-08

**Authors:** Xianwang Shen, Honghong Gong, Yang Zhou, Yucheng Zhao, Jun Lin, Mao Chen

**Affiliations:** a State Key Laboratory of Molecular Engineering of Polymers , Department of Macromolecular Science , Fudan University , Shanghai 200433 , China . Email: chenmao@fudan.edu.cn ; http://chenmaofudan.wixsite.com/polymao; b Key Laboratory of Medicinal Chemistry for Natural Resource , Ministry Education , School of Chemical Science and Technology , Yunnan University , Kunming , 650091 , China

## Abstract

Functionalized cyclooctenes (FCOEs) are important monomers in ring-opening metathesis polymerization (ROMP). Herein, a new library of disubstituted FCOEs bearing adjacent heteroatoms were synthesized and applied in ROMP. To address the issues associated with the handling of the reactive thienyl chloride intermediate, a two-step continuous flow method has been developed to prepare 5-thio-6-chlorocyclooctene compounds from abundant cyclooctadiene starting materials. These newly synthesized FCOE monomers were subsequently polymerized through ROMP, giving rise to a range of functionalized polymers with high molecular weights. Furthermore, we demonstrated that the thermal properties of these polymers could be fine-tuned by changing the functional groups in the FCOE monomers. We expect that this functionalization-polymerization strategy will enable the preparation of a range of polymeric materials with complex structures.

## Abstract 2

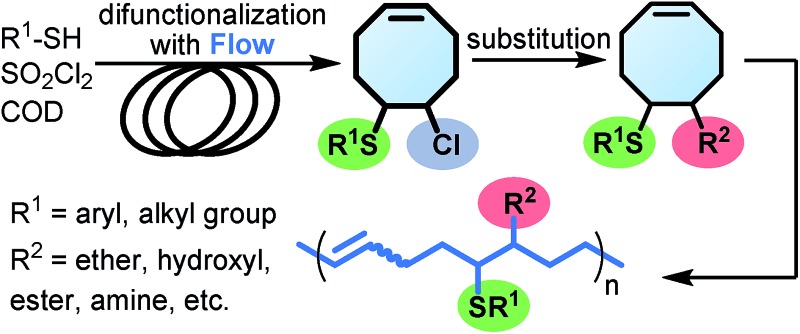
New cyclooctenes have been synthesized under continuous-flow conditions and applied in ring opening metathesis polymerization, providing highly functionalized materials.

## Introduction

The development of synthetic methods to access functionalized polymers is of considerable interest due to the interesting physical and chemical properties associated with these materials. As a result, extensive efforts have been made to accomplish this task by designing well-tailored monomers for different synthetic methods, such as controlled radical polymerization^1^ and ring-opening metathesis polymerization (ROMP).^2^ Alternatively, a number of methods for the postsynthetic modification of polymers have also been developed.^3^ Due to the robustness and functional group tolerance of ROMP, it has become one of the most powerful methods for accessing polymers bearing a wide range of functionalities,^4^ thus enabling the development of materials for drug delivery,^5^ the manipulation of liquids,^6^ ion exchange^7^ and other uses.^8^ While this method is widely utilized, the most frequently used monomers are norbornene, cyclobutene and cyclooctadiene.^4^ A simple method that could provide cyclic olefins with various substituents is important for expanding the scope of functionalized polymers.

**Fig. 1:**
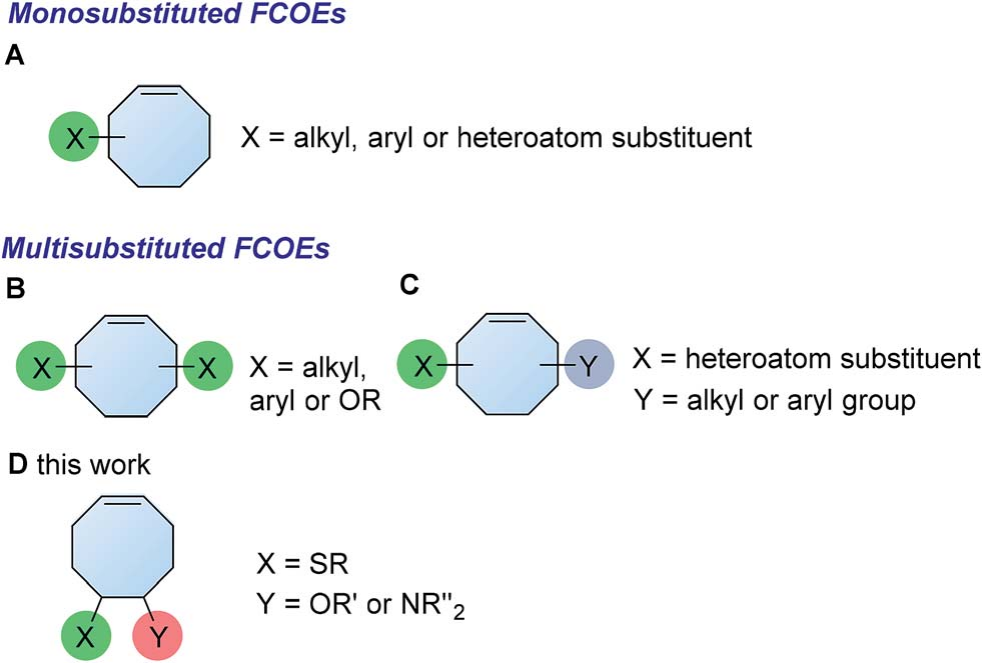
The FCOE toolbox scope for the ROMP study.

FCOE derivatives are a class of the most widely used monomers for ROMP.^6^,8a–e,^9^ Among the many applications of poly(FCOE)s,^6^,8a–e,9a–k ROMP of FCOEs followed by hydrogenation yields linear polyolefins with well-defined chemical structures possessing a wide range of side chains.9a–k This represents a useful approach to high-precision functionalized polyolefins,9a–k which are otherwise difficult to synthesize.^10^ To further explore the utility of ROMP, it is necessary to expand the scope of the FCOEs. Thanks to the efforts devoted to catalyst development and monomer scope exploration, a variety of FCOEs have shown high reactivity in ROMP.^4^,^7^,^8^,9a–k,^11^ Among these, most examples are of mono-substituted compounds (Fig. 1A) prepared *via* C=C bond addition of cyclooctadienes (CODs),^7^,^8^,9a–e,^11^ allyl C–H bond functionalization of cyclooctenes (COEs),9f–j or other methods.9*k*

In contrast to monosubstituted FCOEs, polysubstituted FCOEs are much less investigated for ROMP reactions.9e,^12^ Grubbs and coworkers reported the synthesis and ROMP of symmetrically disubstituted COEs connected with two adjacent hydroxyl groups and their derivatives (Fig. 1B).12a,12b Hillmyer and coworkers reported the preparation and ROMP of ester and methyl/phenyl disubstituted COEs (Fig. 1C).12*c* Nuyken found that the polymerization of dicyano COEs is sluggish, while the monocyano COE polymerizes efficiently.12*d* However, the ROMP of FCOEs possessing different vicinal heteroatoms (Fig. 1D) has not been reported so far. The incorporation of these functional side chains could not only allow for the fine tuning of polymer properties, but also open up new opportunities to introduce orthogonal reactive sites, and is thus highly desirable.

In this regard, we have designed a two-step sequence of thienyl chloride formation/C=C bond addition to prepare FCOEs from *cis*,*cis*-1,5-COD (Fig. 1D: X = SR, Y = Cl). Since the chloride group is easily cleavable through the assistance of the adjacent thioether *via* neighboring group participation,^13^ we envisioned that the 5-Cl,6-SR-COE would be a versatile intermediate to prepare FCOEs with different functionalities (Fig. 1D: X = SR^1^, Y = OR^2^/NR_2_). Although the thienyl chloride (RSCl) species has been known for over half a century, the explosive nature^14^ and unpleasant smell of these compounds somewhat limits their application. Flow processes are useful alternatives to traditional batch procedures.^15^ Many examples have shown the possibility to safely handle hazardous intermediates under flow conditions.^16^ Given our experience with this technique,^17^ we anticipated that a flow approach would significantly enhance the practicality of olefin chlorothiolation processes using thienyl chloride by allowing for the safe and convenient handling of these reactive intermediates.

## Results and discussion

**Scheme 1:**
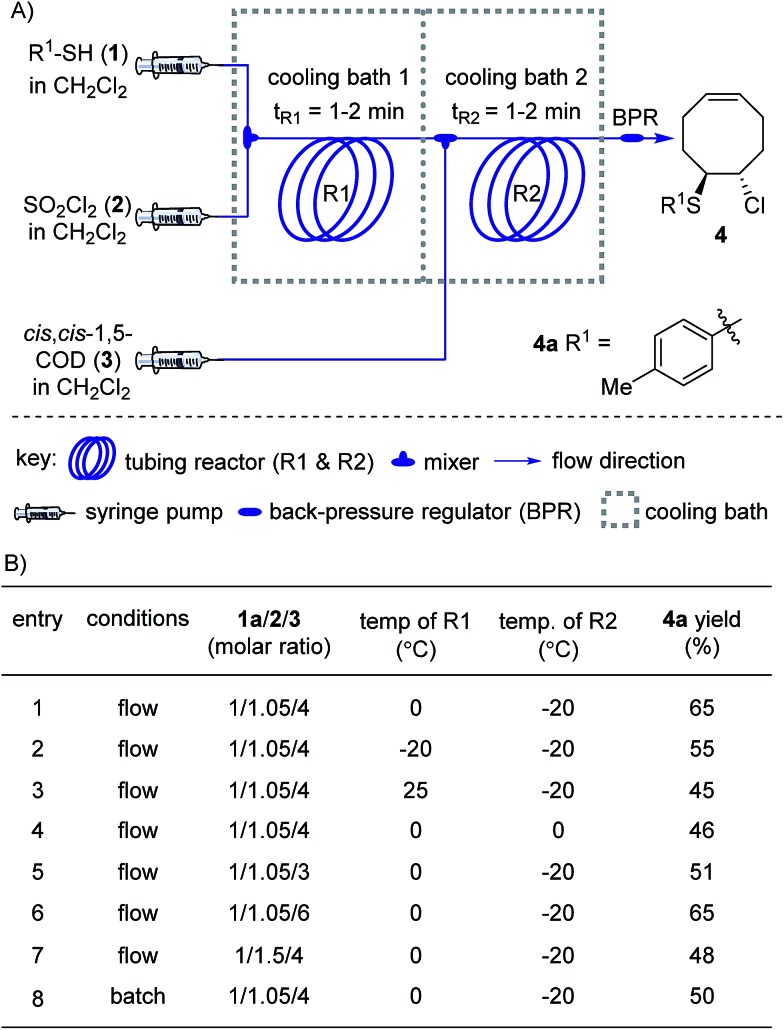
Synthesis of **4** under flow conditions. (A) Schematic of the flow setup. (B) Optimization of the flow conditions. See the ESI for more details.†

We began our studies on the thienyl chloride intermediate formation/difunctionalization sequence with the setup depicted in Scheme 1A with *p*-toluenethiol **1a** as the model substrate. In the flow setup, a solution of **1a** in anhydrous dichloromethane (DCM) was mixed with SO_2_Cl_2_ in anhydrous DCM and introduced into a tubing reactor (R1) immersed in a cooling bath. After the arylthiol was completely converted, as monitored by thin layer chromatography (TLC) analysis, R1 was assembled with the following setup of step II *via* a T-mixer, allowing the solution from R1 to combine with the COD (**3**) solution in-line. The resultant mixture was further delivered into the second tubing reactor (R2), which was submerged in another cooling bath, to perform the direct difunctionalization of the C=C double bond. After the reaction, the mixture was collected and directly analyzed without the isolation of **4a**. Upon investigating a variety of reaction parameters, we determined that the synthesis of **4a** proceeded in good yield with a 1/1.05/4 ratio of **1a**/**2**/**3**, and two reactors cooled at 0 °C and –20 °C respectively (Scheme 1B, entry 1). Notably, this two-step flow method only needed a residence time (*t*_R_) of less than 4 min.^18^ As shown in entries 2 to 7, changing the temperature of either reactor or the molar ratio of the three components resulted in a lower yield of the target product **4a** (see Section II in the ESI†). In contrast, when this reaction sequence was performed under batch conditions only 50% yield of **4a** was obtained in 2.5 h of reaction time, as detected by ^1^H NMR analysis.

Following the two-step flow synthesis (Table 1, step I & II), the solution of compound **4a** was directly added into a vial with anhydrous methanol at room temperature (Table 1, step III). The Cl group on **4a** was efficiently replaced by a OMe group under mild conditions within several hours, as monitored by TLC analysis. The resultant mixture was purified by silica gel column chromatography to afford FCOE **5a** in 64% yield over three steps. In comparison, when 5-Cl-1-cyclooctene^19^ was reacted with MeOH at room temperature for 48 h instead of **4a**, no substitution product was detected by LC-MS, supporting our hypothesis of a vicinal SR group assisted substitution process.^13^

**Table 1:** Synthesis and ROMP of **5a–5g**^*a*^

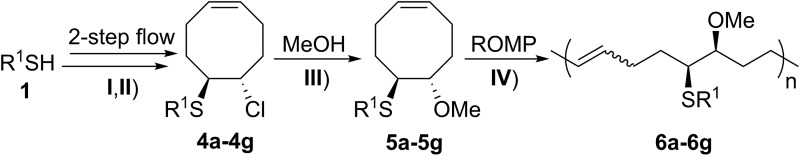								
Entry	R^1^	**5** yield^*b*^ (%)	[**5**]_0_/[G2]	**5** conv^*c*^ (%)	**6** yield^*d*^ (%)	*M*_n,calc_ ^*e*^ (kg mol^–1^)	*M*_n,GPC_ ^*f*^ (kg mol^–1^)	*Đ* ^*f*^
1	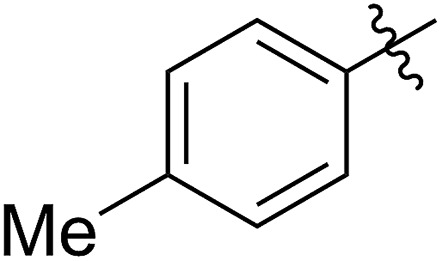	64 (**5a**)	500/1	>99	96 (**6a**)	131	148	1.71
2	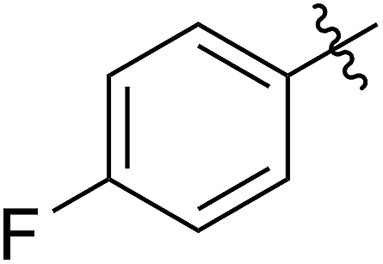	68 (**5b**)	1000/1	>99	95 (**6b**)	266	291	1.49
3	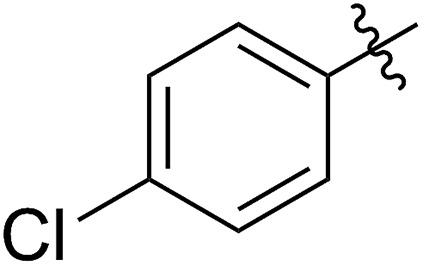	55 (**5c**)	500/1	>99	93 (**6c**)	142	159	1.68
4	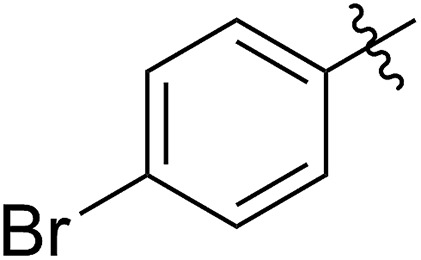	56 (**5d**)	400/1	>99	90 (**6d**)	131	106	1.68
5	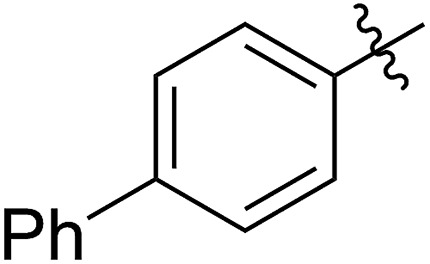	64 (**5e**)	500/1	>99	91 (**6e**)	163	226	1.69
6	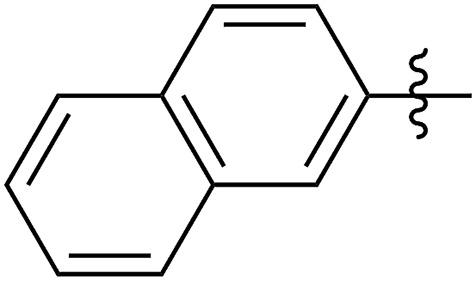	63 (**5f**)	500/1	>99	93 (**6f**)	149	201	1.73
7	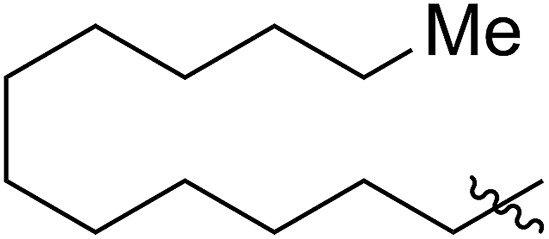	70 (**5g**)	1000/1	36	31 (**6g**)	127	311	1.67

^*a*^Reaction conditions for (I) to (IV): (I, II) **4** was synthesized using the conditions shown in Scheme 1B, entry 1; (III) rt, 4 hours, anhydrous MeOH (10 eq. to **4**); (IV) G2 carbene complex was used to initiate the ROMP, DCM, rt.

^*b*^Isolated yields of the three steps, calculated based on R^1^SH.

^*c*^Calculated based on the amount of the recovered monomer by column chromatography.

^*d*^Isolated yields were calculated based on the monomers added in the ROMP.

^*e*^Calculated based on the conversions of the FCOEs **5**.

^*f*^Analyzed by GPC.

With the method established for the preparation of **5a**, we turned our attention to the synthesis of FCOEs with different SR^1^ substituents. To our delight, all the R^1^SH substrates (**1**) investigated in Table 1 underwent complete conversion to **5b–5g** in about 4 h of reaction time (Table 1, step I to III). After the consecutive three-step transformations, the resultant mixtures were purified by silica gel column chromatography to afford the FCOEs **5b–5g** in satisfactory yields (55–70%). Notably, since aryl halides (*e.g.* Cl and Br) are versatile functional groups in metal-catalysed cross-coupling reactions, the incorporation of such groups (**5c** and **5d**) would bring in reactivity orthogonal to the substituent on the COE backbone.^20^ All FCOE monomers were characterized by nuclear magnetic resonance (NMR), infrared radiation (IR), and high-resolution mass spectroscopy (HRMS) analysis (Section III and Fig. S3–S23†), demonstrating the successful introduction of the two adjacent heteroatom substituents SR^1^ and OMe into the COEs.

Moreover, to streamline the synthesis of the FCOEs **5**, a three-step continuous-flow setup has been developed (Fig. S2†) using a pressurised heating system at 80 °C for step III. As exemplified with **5a**, the reaction time was reduced to 20 min, facilitated by the efficient heat transfer under the flow conditions, affording **5a** in 66% isolated yield.

The 5-SR^1^,6-OMe-COE monomers **5a–5g** were polymerized with the second-generation Grubbs carbene complex (G2) in DCM at room temperature (step IV).^21^ As illustrated in Table 1, full conversions of all monomers upon G2-catalyzed ROMP was achieved when the arylthio group was substituted with an electron-donating group (Me, entry 1, **5a**), an electron-withdrawing group (F, entry 2, **5b**; Cl, entry 3, **5c**; Br, entry 4, **5d**), or a phenyl group (entry 5, **5e**), affording a variety of functionalized polymers in high yields (**6a–6f**: 90–96% yields) following isolation *via* a three-time precipitation from methanol. Similar to the Ru-promoted ROMP of alkylthio mono-substituted COEs reported by Noels and coworkers,11*d* when 5-*n*C_12_H_25_S,6-MeO-COE (**5g**, entry 7) was used a decreased polymerizing reactivity was observed, providing **6g** (*M*_n,GPC_ = 311 kg mol^–1^, *M*_w_/*M*_n_ = 1.67) with 36% monomer conversion in 48 h of reaction time. This is probably due to the increased coordinating effect of an alkylthio group to the metal center compared to that of an arylthiol group. For all examples (**5a–5g**) investigated in Table 1, high molecular weight polymers (*M*_n,GPC_ = 106–311 kg mol^–1^, *Đ* = 1.49–1.73) were obtained, further confirming the reliability of the ROMP of these new FCOEs (Section IV and Fig. S24–S58†). Notably, polymers **6a–6g** have the same chemical component, with butadiene/vinyl ether/vinyl thioether terpolymers present in a 1/1/1 molar ratio for each monomer, representing a novel group of functionalized polyolefins.

It has been shown that substitution of the chloro group on substrates **4** with a methoxy group is efficient, and that FCOEs **5** were successfully polymerized. We further focused on expanding the ROMP substrate scope by replacing the Cl group with other functionalities.

A solution containing COE **4** freshly prepared *via* a flow process was concentrated and treated with silica gel chromatography using 0–2% (v/v) EtOAc in petroleum ether as an eluent. During the column chromatography process, **4a** underwent a full hydrolysis within 30 min, resulting in the cyclic olefin **7a** which has a hydroxy handle. Upon reaction with different electrophiles (step IV), the hydroxy handle was readily connected to a *t*-butyldimethylsilane (TBS, **7b**, **7c**), a benzyl (Bn, **7d**), or an acetyl (MeCO, **7e**) group. Additionally, the chloro group was also converted to an *N*-heteroatom containing substituent by simply reacting with a nucleophile (step V, *e.g.* morpholine, **7f**). Although 3–4 steps were employed, compounds **7a** to **7f** were isolated in good overall yields, and these compounds were characterized by NMR, IR and HRMS analysis (Section V and Fig. S59–S82†). To further identify the FCOE structure, **7f** was analysed by X-ray crystallography (Table 2, bottom left). While the C=C double bond keeps a *cis* configuration, the SAr group and the morpholine group are *trans* to each other. This is consistent with the vicinal SR group assisted substitution process, which could proceed through a thiiranium ion intermediate.13a–d

**Table 2:** Synthesis and ROMP of **7a–7h**^*a*^ 
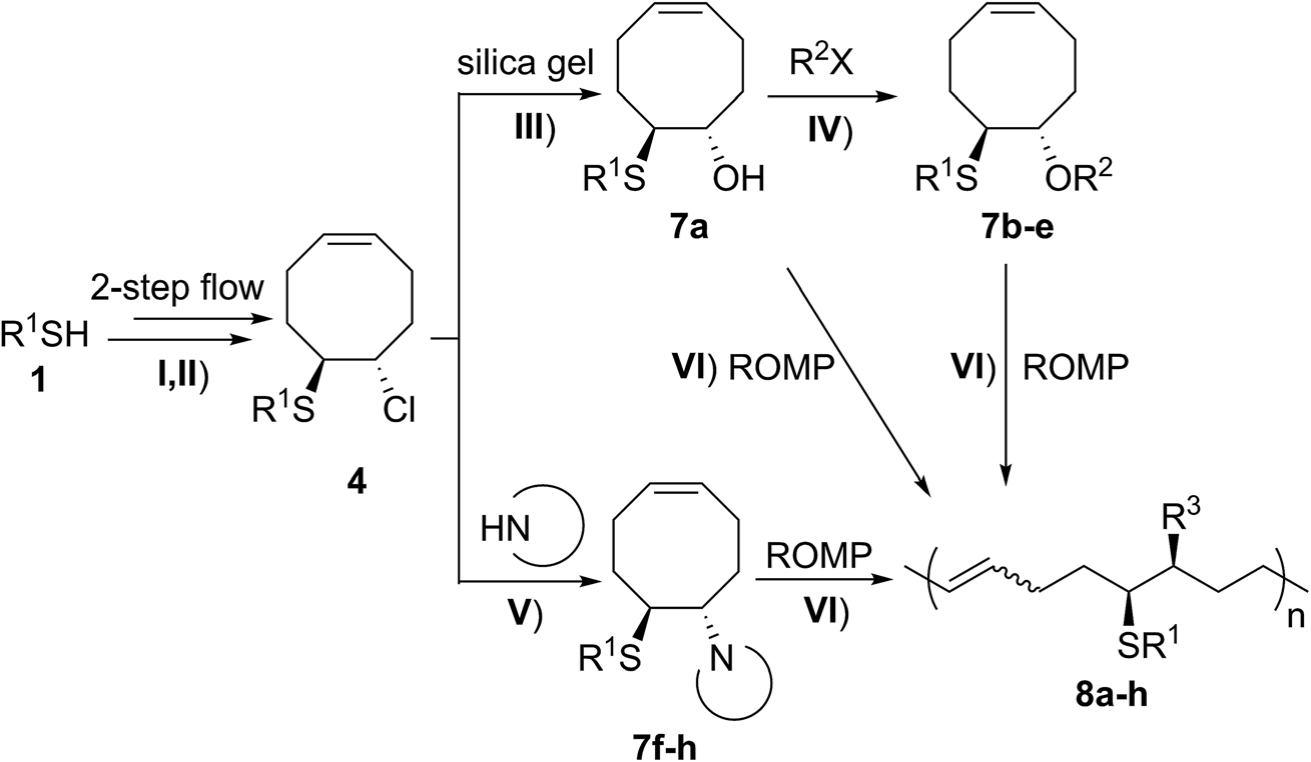

Entry	R^1^	R^3^	**7** yield^*b*^ (%)	**7** conv^*c*^ (%)	**8** yield^*d*^ (%)	*M*_n,calc_ ^*e*^ (kg mol^–1^)	*M*_n,GPC_ ^*f*^ (kg mol^–1^)	*Đ* ^*f*^
1	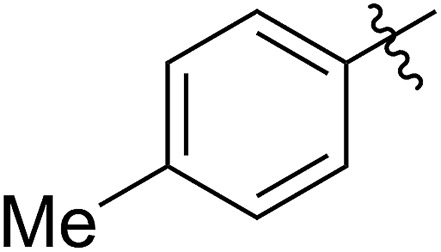	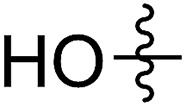	67 (**7a**)	41	20 (**8a**)	51	80	1.66
2^*h*^				>99	90 (**8a′**)	6.2	6.8	1.65
3^*i*^	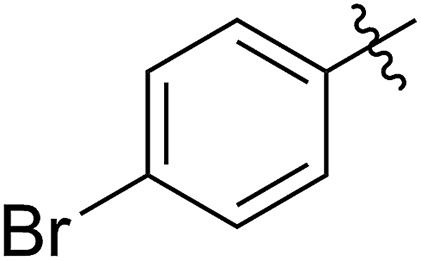	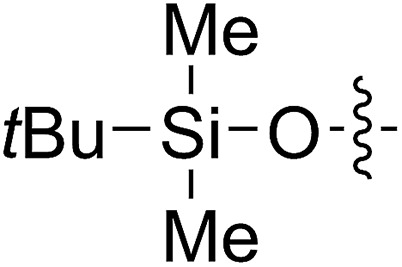	63 (**7b**)	>99	81 (**8b**)	85	109	1.71
4^*i*^ ^,^^*j*^			(**7b**)	>99	82 (**8b′**)	85	106	1.62
5	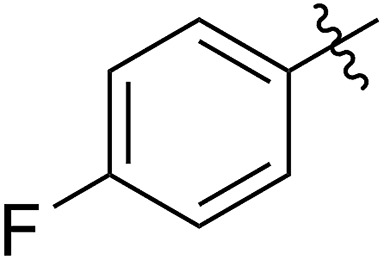	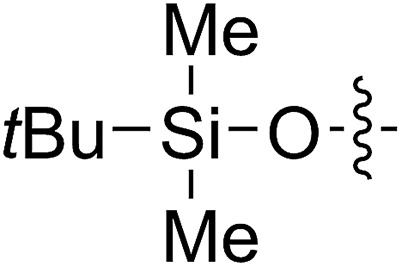	65 (**7c**)	>99	78 (**8c**)	183	160	1.76
6	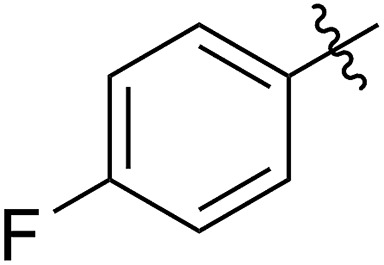	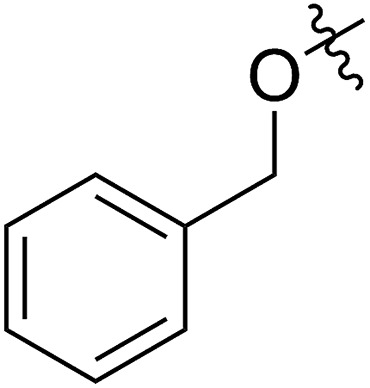	62 (**7d**)	>65	45 (**8d**)	111	71	1.57
7	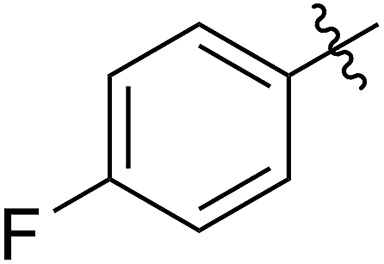	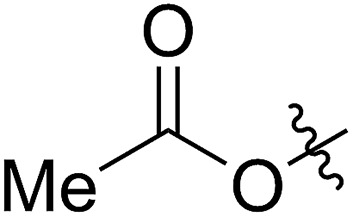	54 (**7e**)	>90	77 (**8e**)	125	104	1.58
8	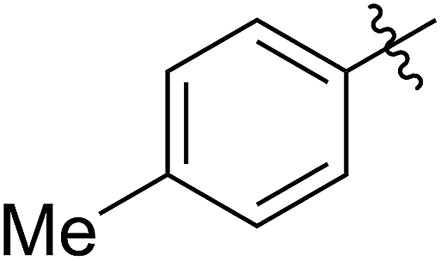	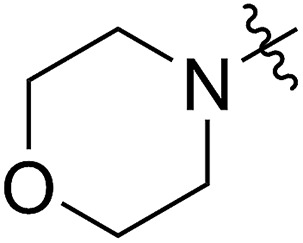	59 (**7f**)	92	82 (**8f**)	146	193	1.78
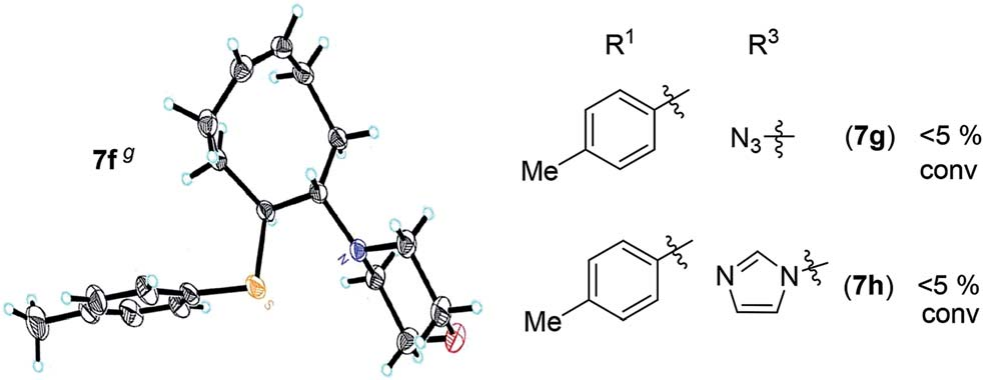								

^*a*^Reaction conditions for (I) to (IV): (I, II) **4** was synthesized using the optimized conditions shown in Scheme 1B, entry 1; (III) silica gel; (IV) **7b** and **7c**: TBSCl, imidazole, DMAP, DCM, 0 °C to rt; **7d**: BnBr, NaH, 0 °C to rt; **7e**: AcOH, DCC, DMAP, DCM, 0 °C to rt; (V) **7f**: morpholine, rt; **7g** N(*n*Bu)_4_N_3_, rt; **7h**: imidazole, rt; (VI) G2 was used to initiate the ROMP, [M]/[G2] = 500/1, room temperature.

^*b*^Isolated yields of three steps (**7a** and **7f**) or four steps (**7b–7e**), calculated based on R^1^SH.

^*c*^Calculated based on the amount of the recovered monomer by column chromatography.

^*d*^Isolated yields were calculated based on the monomers added in the ROMP.

^*e*^Calculated based on the conversions of **7**.

^*f*^Analyzed by GPC.

^*g*^X-ray structure of **7f**.

^*h*^[M]/[G2] = 20/1.

^*i*^[M]/[G2] = 200/1.

^*j*^Reaction temperature = 45 °C.

The newly synthesized FCOE monomers (**7a–7h**) were next polymerized in the presence of G2 at room temperature (Table 2, step VI).^22^ When FCOE **7a** with an unprotected hydroxy group was employed in a [**7a**]/[G2] ratio of 500/1, less than 50% conversion was achieved in 48 h of reaction time, providing **8a** in 20% isolated yield (*M*_n,GPC_ = 80 kDa mol^–1^, entry 1). Although decreasing the monomer/G2 ratio to 20/1 led to complete monomer conversion within 24 h, **8a′** with a much lower *M*_n,GPC_ of 6.8 kDa was provided (entry 2), with a *Đ* value similar to **8a** (for **8a**, *Đ* = 1.66, for **8a′**, *Đ* = 1.65). We hypothesized that the improved monomer conversion was due to less of the transition-metal being poisoned by increasing the G2/monomer ratio. When the reaction temperature was increased from room temperature to 45 °C, poly(FCOE)s were generated with a similar *M*_n_ and slightly improved control over the molecular weight distributions (entry 3, *M*_n_ = 109 kDa and *Đ* = 1.71 *vs.* entry 4, *M*_n_ = 106 kDa and *Đ* = 1.62). When the third-generation of Grubbs carbene complex (G3) was used to initiate the ROMP of **7b** ([**7b**]/[G3] = 200/1) at room temperature, the corresponding polymer was produced with *Đ* = 1.65 and *M*_n_ = 94 kDa at >99% conversion.

To produce poly(FOE)s with high molecular weights, a monomer/G2 ratio of 500/1 was used during the ROMP reaction of the other FOCEs. When **7c–7e** were applied in the ROMP for 24 h, isolated yields of 45–78% were obtained for the polymers **8c–8e** with *M*_n,GPC_ values of 71–160 kDa (entries 5–7). When the SR^1^ group was adjacent to a morpholine group instead, polymer **8f** was isolated in 82% yield (*M*_n,GPC_ = 193 kg mol^–1^, entry 8). Both NMR and IR analyses clearly demonstrate that both types of functional group have been successfully incorporated in polymers **8a–8f** (Section VI and Fig. S83–S112†). Replacing the morpholine group with an azide or an imidazole group provided less than 5% monomer conversion, which is probably caused by the irreversible coordination of the functional group to the Ru-center, as observed by Noels and coworkers.11*d* Notably, these represent the first ROMPs of FCOEs possessing adjacent substituents of SR^1^ and OR^2^/NR_2_ functionalities.

**Fig. 2:**
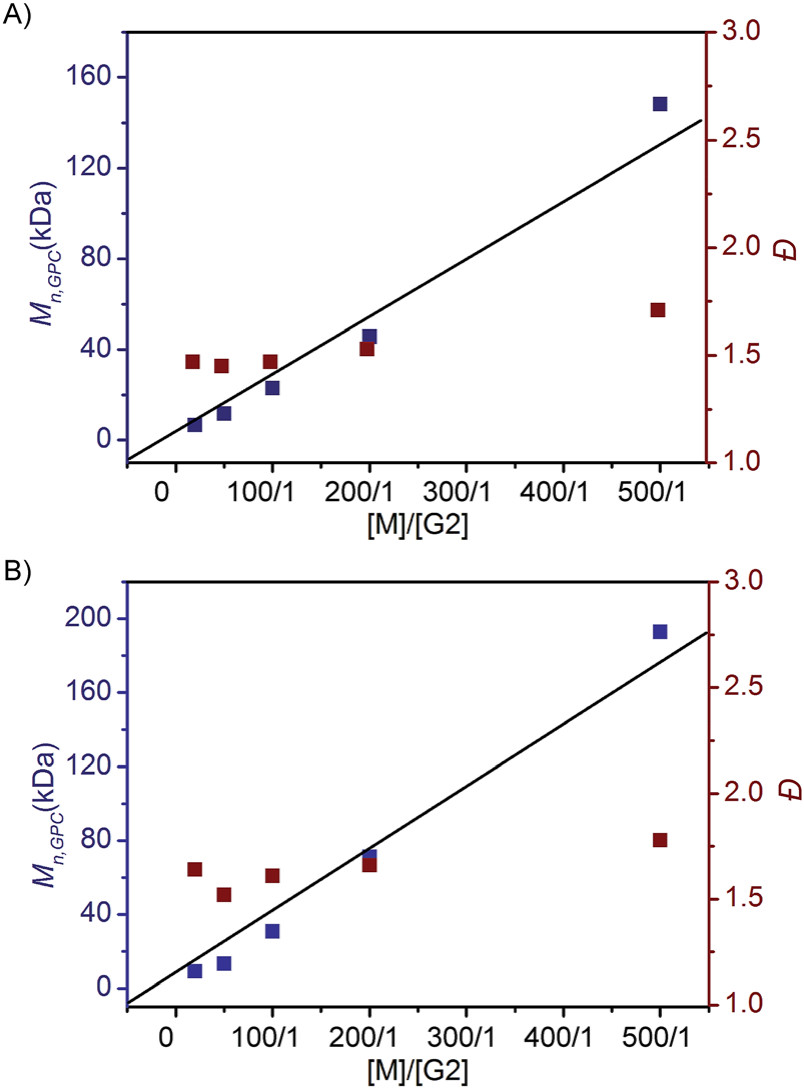
ROMP of the FCOEs at different [M]/[G2] ratios (20/1, 50/1, 100/1, 200/1 and 500/1) for 24 h in DCM. *M*_n,GPC_ and values were analysed by GPC. (A) **5a** was used. (B) **7f** was used.

To investigate the ROMP of difunctionalized FCOEs at different monomer/G2 ratios, **5a** and **7f** were employed. As shown in Fig. 2, when the [M]/[G2] ratios were increased from 20/1 to 500/1 for both monomers, poly(FCOE)s were produced with different *M*_n,GPC_ values, while the *Đ* values stayed at a similar level (*Đ* = 1.47–1.71 in Fig. 2A, *Đ* = 1.52–1.78 in Fig. 2B). Notably, a linear increase of *M*_n,GPC_*vs.* [M]/[G2] was observed for both examples, which demonstrated that these poly(FCOE)s can be generated at the desired *M*_n_ by choosing a proper [M]/[G2] ratio within the investigated range.^22^

**Fig. 3:**
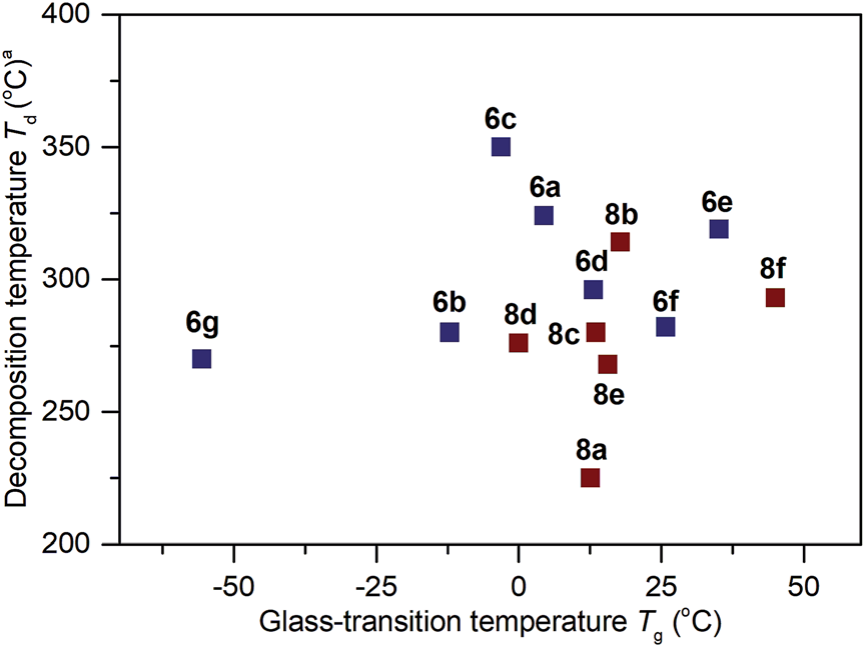
Thermal properties of the polymers. The *T*_g_ and *T*_d_ values were determined by DSC and TGA measurements, respectively. All values were obtained under a nitrogen atmosphere at a scan rate of 10 °C min^–1^. DSC experiments were conducted between –80 to 200 °C. Temperatures at 5% weight loss (*T*_d_) are given.

The thermal properties for the polymers **6a–6g** and **8a–8f** were analyzed by differential scanning calorimetry (DSC) and thermogravimetric analysis (TGA). The summarized results of their glass-transition temperature (*T*_g_) and decomposition temperature (*T*_d_) are shown in Fig. 3 23,24 (for the DSC and TGA profiles, see Section IV and VI of the ESI†). From **6a** to **6g**, while keeping the MeO group constant, changing the alkylthio side chains to arylthio chains resulted in polymers possessing increased *T*_g_ values (**6g**: –56 °C *vs.***6a–6f**: –12 °C to 35 °C). Among **6b–6g**, an increased functional group size on the aryl ring (from **6b** to **6e**: –12 °C, –3 °C, 13 °C and 35 °C respectively) or an increased degree of conjugation (*e.g.*, **6f**: 26 °C *vs.***6a**: 4 °C) led to increased *T*_g_ values. These results are in agreement with the sidechain influence on the glass transition temperature, as observed by others.9g,24a,24b For the polymers **8b–8e**, when the hydroxy side groups were protected with groups larger than methyl, the resultant *T*_g_ values were higher than **6b** (**8b–8e**: 0–18 °C *vs.***6b**: –12 °C). Replacement of the MeO group with a morpholine group also led to an increased glass-transition temperature (**8f**: 45 °C *vs.***6a**: 4 °C). The thermogravimetric analysis in Fig. 3 shows that the thermal stabilities of these polymers are also dictated by the connection of different functional groups. Polymers **6a–6g** and **8a–8f** possess *T*_d_ values ranging from 225 °C to 350 °C at 5% weight loss.

**Fig. 4:**
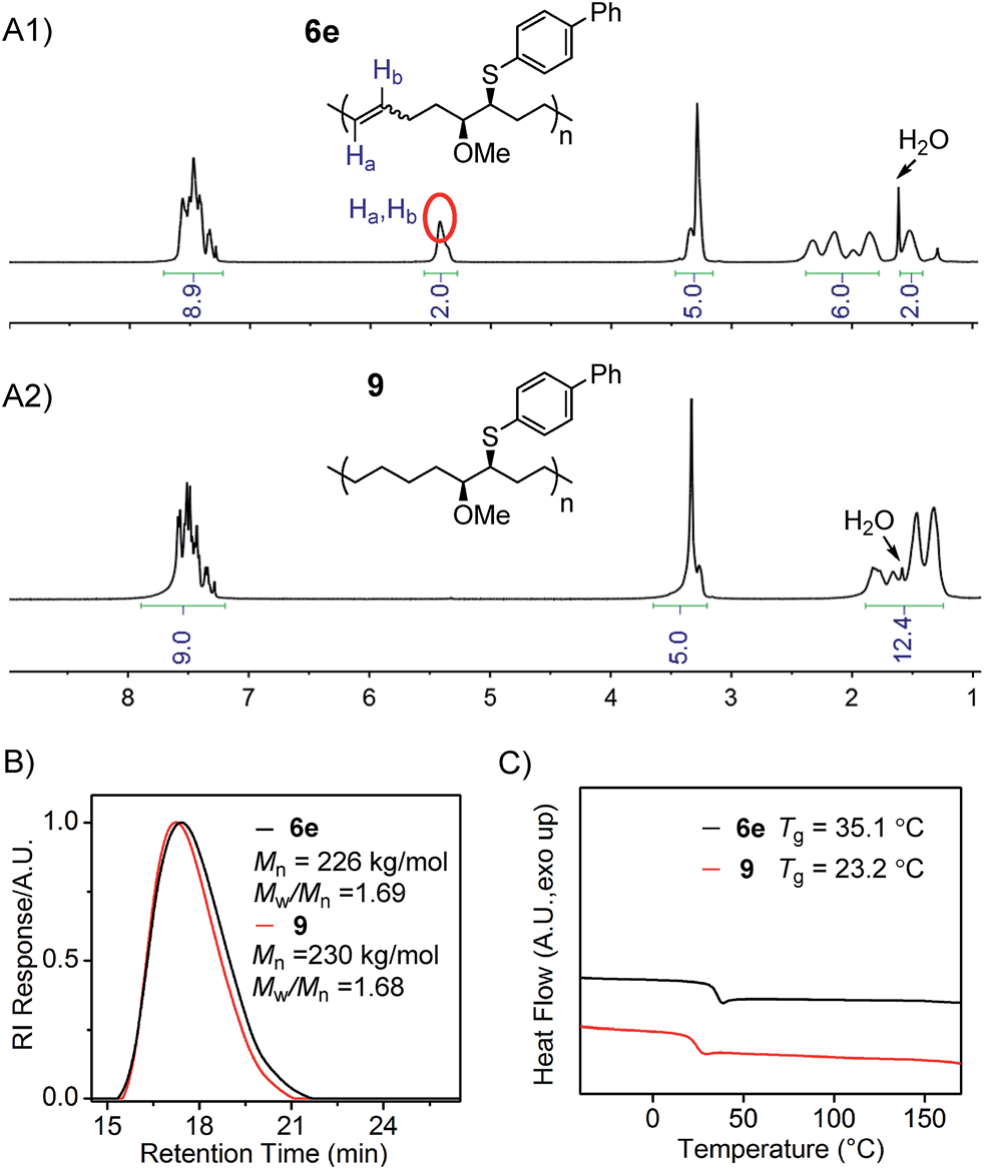
Characterization of polymers **6e** and **9**. (A1) and (A2) ^1^H NMR spectra; (B) GPC traces, *M*_n_ and *M*_w_/*M*_n_ values are analysed with GPC; (C) DSC profiles obtained at a heating rate of 10 °C min^–1^.

Finally, the hydrogenation of polymer **6e** was conducted to demonstrate the preparation of linear polyolefins possessing two different side chains on every seventh and eighth backbone carbon, from the corresponding poly(FCOE)s. The hydrogenation reaction was performed using *p*-toluenesulfonylhydrazide as the reductant and tri-*n*-hexylamine as the base with a catalytic amount of 2,6-di-*t*-butyl-4-methylphenol (BHT) in *o*-xylene solvent.9c–k,^25^ The reduced product **9** was obtained in 88% isolated yield *via* precipitation from methanol. As shown in the ^1^H NMR spectra (Fig. 4A1 and A2; Section VIII and Fig. S104–S108†), during the hydrogenation process, the signals found between 5.5–5.3 ppm corresponding to H_a_ and H_b_ of polymer **6e** are completely absent in the spectrum of polymer **9**. As a result, an increase in the signal region corresponding to alkyl protons is clearly observed for polymer **9** (Fig. 4A1 *vs.*4A2 in the 1.0–2.5 ppm region), indicating the successful hydrogenation transformation. The GPC analyses of **6e** and **9** (Fig. 4B) show: (1) similar *M*_n,GPC_ and *M*_w_/*M*_n_ values, and (2) no new shoulder peaks in the GPC traces, suggesting that the polymer backbone remains intact during the reduction process. Moreover, the hydrogenated polymer **9** has a lower *T*_g_ value than **6e** (Fig. 4C), indicating that the formation of a saturated backbone results in a higher molecular mobility. Hillmyer9*g* and Tanaka9f,9h have also reported a decrease in the *T*_g_ values upon hydrogenating the corresponding poly(FCOE)s.

## Conclusions

In conclusion, the synthesis and ROMP of FCOEs bearing adjacent heteroatom groups have been successfully realized. Notably, the unstable thienyl chloride species has been generated and used under flow conditions for the first time, allowing for an efficient synthesis of 5-SR,6-Cl-COE compounds, which were employed as versatile intermediates for the preparation of a library of FCOEs. Moreover, the ROMP of these new cyclic monomers has produced a library of polyolefins with different substituents connected by S, O or N heteroatoms in high molecular weights. This represents a useful avenue to synthesize polymers with a high level of complexity. The investigation of the thermal properties of these functionalized polymers has shown the effect of the side chains on their glass-transition temperatures and thermal stabilities. Finally, this approach complements the useful strategy of producing high precision model polyolefins *via* ROMP, allowing the preparation of terpolymers of ethylene, vinyl thioether, and a variety of polar olefins including vinyl ethers, vinyl esters and vinyl amines, which are inaccessible *via* other methods.

## Experimental

The experimental procedure for the preparation of **5a** with the optimized reaction conditions: a syringe was loaded with a solution of *p*-toluenethiol **1a** (1.0 M, flow rate = 250 μL min^–1^) in anhydrous DCM, and fitted to the syringe pump. Another syringe was loaded with a solution of **2** (1.05 M, flow rate = 250 μL min^–1^) in anhydrous DCM, and fitted to a same syringe pump. The third syringe was loaded with a solution of COD (0.5 M, flow rate = 2.0 mL min^–1^) in anhydrous DCM, and fitted to the second syringe pump. Following the setup as shown in Scheme 1, the solutions of **1a** and **2** were mixed and reacted in the tubing reactor R1 (volume = 1.0 mL, *t*_R1_ = 2.0 min) submerged in a cooling bath. When the reaction was complete, the resultant solution was mixed with the solution of COD and reacted in the tubing reactor R2 (volume = 5 mL, *t*_R2_ = 2.0 min) submerged in another cooling bath. After the reaction, the resultant mixture was passed through a back-pressure regulator (BPR, 20 psi) before collection. After reaching steady state (waiting for 12 min), 1.0 mmol samples (10 mL reaction solution) were collected into an oven-dried vial equipped with a stir bar.

Anhydrous MeOH (10 mmol) was added into the vial *via* syringe at room temperature. When the reaction was completed, as monitored by TLC analysis, the mixture was treated with DCM (150 mL) and NaHCO_3_ saturated aqueous solution (20 mL). The separated organic layer was washed with brine two times (2 × 10 mL), dried over Na_2_SO_4_ and then concentrated under vacuum. The residue was purified by column chromatography (eluting with 0–2% EtOAc in petroleum ether) to afford **5a** in 64% isolated yield.

An oven-dried vial equipped with a stir bar was charged with a 1.0 mL solution of **5a** (0.5 M) in anhydrous DCM under N_2_. The G2 compound solution (100 μL, 8.5 mg mL^–1^ in degassed DCM) was added *via* micro syringe into the vial at room temperature. After stirring for 24 h, the mixture was concentrated and then added dropwise into MeOH with vigorous stirring. The solid compound was collected and re-dissolved in a minimal amount of DCM. The precipitation procedure was repeated three times in total to afford the target product. The produced polymer was characterized by ^1^H NMR, ^13^C NMR, FT-IR, GPC, DSC and TGA analysis.

## Conflicts of interest

There are no conflicts to declare.

## Supplementary Material

Supplementary information

Crystal structure data
