# Supplementary material for: Unsymmetrical difunctionalization of cyclooctadiene under continuous flow conditions: expanding the scope of ring opening metathesis polymerization
Source: Chem Sci. 2018 Jan 8;9(7):1846–53. doi: 10.1039/c7sc04580h (PMC5890785; doi:10.1039/c7sc04580h)
Supplement: Supplementary file 1 [file SC-009-C7SC04580H-s001.pdf]

Electronic Supplementary Material (ESI) for Chemical Science.
This journal is © The Royal Society of Chemistry 2018

Supporting Information—Table of Contents

Section I: *General Information of Materials and Analytical Methods*

Section II: *Experimental Procedures of the flow synthesis*

Section III: *Synthesis and Characterization of Monomers***5a-5g**

Section IV: *Synthesis and Characterization of Polymers* ***6a-6g***

Section V: *Synthesis and Characterization of Monomers* 7***a-7h***

Section VI: *Synthesis and Characterization of Polymer*s ***8a-8f***

Section VII *Synthesis and Characterization of Polymer*s ***9***

Section VIII *X-ray Characterization of* ***7f***

***Section I**: General Information of Materials and Analytical Methods*

Tetrahydrofuran (THF) was freshly distilled from sodium. Dichloromethane (DCM) were freshly distilled from CaH_2. Arylthiolsand alkylthiols were purified with a column chromatography on silica gel before use. Sulfuryl chloride and COD were distilled before use. Anhydrous methanol, Grubbs second generation catalyst, other reagents and solvents were purchased from Sigma-Aldrich, Adamas or TCI, and were used as received without further purification.

Nuclear magnetic resonance (NMR) was recorded on an AvanceIII 400 MHz Bruker spectrometer at 298 K. ^{1}H NMR signals were measured relative to the signal for residual chloroform (7.26 ppm) in deuterochloroform ($CDCl_3$), and are reported in δ units, parts per million (ppm). ^{13}C NMR signals were obtained are reported in ppm units relative to $CDCl_3$ (77.16 ppm). Gel permeation chromatography (GPC) measurements were performed in THF at 35 °C with an elution rate of 1.0 mL/min on an Agilent 1100 equipped with a G1310A pump, a G1362A refractive index detector. Three columns were employed including one 5 μm LP gel column (molecular range 500–2×10^4 g mol^{-1}) and two 5 μm LP gel mixed bed columns (molecular range 200–3×10^6 g mol^{-1}). The calibration was performed with PS standards. Infrared (IR) spectra were recorded on a ThermoScientific Nicolet 6700 FT-IR instrument. High resolution mass spectra (HRMS) were measured with a Waters Micromass GCT instrument, accurate masses are reported for the molecular ion ($[M+H]^+$). The thermogravimetric analysis (TGA) of polymers was performed using a Perkin Elmer Pyris 1 at a heating rate of 20 °C min^{-1}. Differential scanning calorimetry (DSC) was carried out on a DSC Q2000 thermal analysis system (Shimadzu, Japan). The samples were first heated from -80°C to 200 °C at a heating rate of 10 °C min^{-1} under a nitrogen atmosphere, followed by cooling to -80 °C at −10 °C min^{-1} after stopping at 200 °C for 3 minutes. Column chromatography was carried out using silica gel (230-400 mesh).

***Section II**: Experimental Procedures of the flow synthesis*

***II-1:** General Material Information*

All tubings, connectors, nuts, ferrules, fittings and back-pressure regulators were purchased from IDEX Health and Science, unless otherwise stated. Syringe pumps were

purchased from Longer company. The equipment configurations that were used for the flow reactions are depicted in Figures S1. The tubing reactors and all connecting tubing in all figures were made of plastic tubing (0.0625" OD × 0.04" ID, or 0.125" OD × 0.0625" ID). Connections for all reactors were made using super flangeless ferrules and super flangeless nuts. The Y-mixers (0.04" ID) were used for mixing different solutions. Refrigerated bath circulator (-20 ºC) and ice water bath (0 ºC) were used for the cooling purpose. Oil bath (80 ºC) were used for the heating purpose.

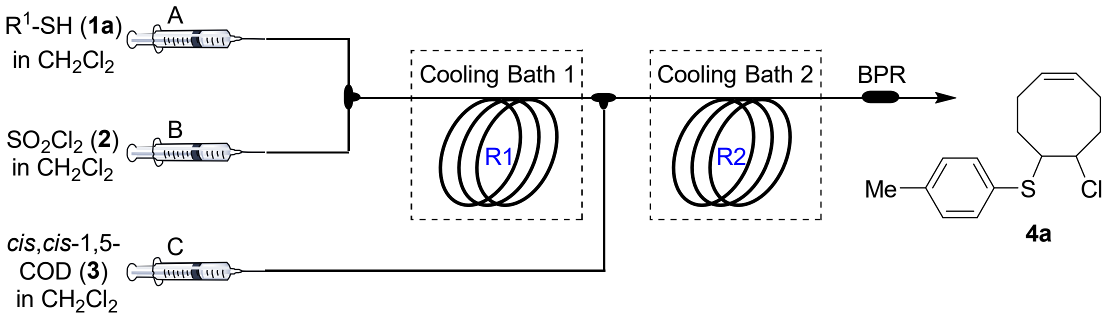

Figure S1. Flow setup for the synthesis of **4**

II-2: General procedure for flow experiments

Syringe A was loaded with the solution of *p*-toluenethiol **1a** (1.0 M) in anhydrous DCM, and fitted to a syringe pump. Syringe B was loaded with the solution of **2** (1.0-1.5 M) in anhydrous DCM, and fitted to a same syringe pump. Syringe C was loaded with the solution of COD (0.5 M) in anhydrous DCM, and fitted to the second syringe pump. Following the setup as shown in Figure S1, solutions of **1a** and **2** were mixed and reacted in tubing reactor R1 (2 min) submerged in cooling bath 1. When the reaction was complete, the resulting solution was mixed with the solution of COD and reacted in tubing reactor R2 (2 min) submerged in cooling bath 2. After reaction, the resulting mixture was passed through a back-pressure regulator (BPR 20 psi) before collection. After reaching steady state (normally waiting for 12 min), 0.5-2.0 mmol (5.0-20 mL) samples were collected into oven-dried vials for further reaction step. A small aliquot of the resulting mixture was directly analyzed with TLC and NMR.

Table S1

Entry	**1a**/**2**/**3**	Concentration (mmol/mL)			Flow rate (mL/min)			Volume of reactor (mL)	
		**1a**	**2**	**3**	**1a**	**2**	**3**	R1	R2
1	1/1.05/4	1.0	1.05	0.5	0.25	0.25	2.0	1	5
2	1/1.05/3	1.0	1.05	0.5	0.25	0.25	1.5	1	4
3	1/1.05/6	1.0	1.05	0.5	0.25	0.25	3.0	1	7
4	1/1.50/4	1.0	1.5	0.5	0.25	0.25	2.0	1	5

II-3: *Synthesis of* ***4a*** *under batch conditions*

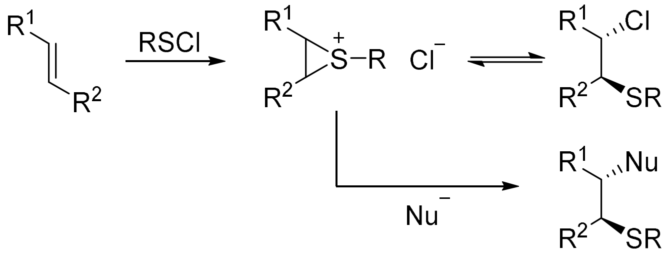

Scheme S1. Proposed mechanism for the vicinal SR group assisted substitution.

An oven-dried flask equipped with a stir bar was charged with *p*-toluenethiol **1a** (124.2 mg, 1 mmol) and 2 mL anhydrous DCM at 0 ºC. 1mL sulfuryl dichloride (1.05 M) in DCM was dropwisely added into the flask under nitrogen atmosphere at 0 ºC. The color of the solution changes to yellow. The mixture was stirred at 0 ºC for 20 min to afford the complete conversion of *p*-toluenethiol as monitored by TLC analysis before next step. Another oven-dried flask equipped with a stir bar was charged with COD (4.0 mmol) and 8 mL DCM at -20 ºC. The previously prepared solution was dropwisely added into the flask under nitrogen at -20 ºC. After stirring at -20 ºC for 2 h, the mixture was treated $NaHCO_3$ saturated aqueous solution and extracted with DCM. The organic layer was dried over Na_2SO_4 and concentrated under vacuum. A small aliquot of the resulting mixture was directly analyzed with TLC and NMR.

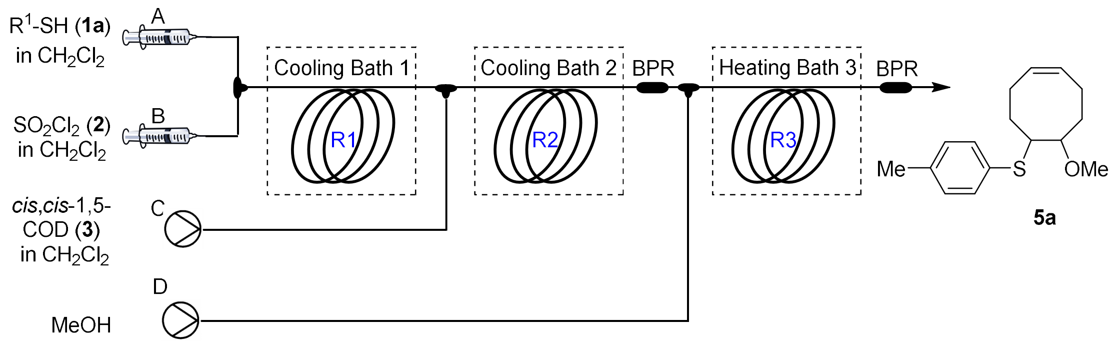

Figure S2. Flow setup for the three-step continuous-flow synthesis of **5**

II-4: *Experimental procedure for the synthesis of* ***5a*** *under flow conditions*

Following the optimized two-step synthesis of **4a** under continuous-flow conditions, the produced solution was directly mixed with anhydrous methanol (introduced with a HPLC pump, flow rate 0.2 mL/min) in line with a T-mixer as shown in Figure S2. The resulted mixture was subsequently introduced into R3 (20 min) submerged in a pre-heated oil bath at 80 ºC. After reaction, the solution passed through a BPR (100 psi) before collection. After reaching steady state as monitored with TLC, 7 mL solution was collected (based on 0.5 mmol of **1a**) into a vial. The mixture was concentrated under vacuum. The residue was purified by column chromatography to afford **5a** in 66% isolated yield.

Entry	Concentration (mmol/mL)			Flow rate (mL/min)				Volume of reactor (mL)		
	**1a**	**2**	**3**	**1a**	**2**	**3**	MeOH	R1	R2	R3
1	1.0	1.05	0.5	0.05	0.05	0.4	0.20	0.2	1.0	14

Section III: *Synthesis and Characterization of Monomers* ***5a-5g***

III-1: *General procedure for the synthesis of* ***5a-5g***

After 1.0 mmol (10 mL) solution of intermediate **4** was collected into an oven-dried vial equipped with a stir bar, anhydrous MeOH (10 eq.) were added via a syringe at room temperature. When the reaction was completed as monitored by TLC analysis, the mixture was treated with DCM (150 mL) and $NaHCO_3$ (20 mL) saturated aqueous solution. The separated organic layer was washed brine for two times (2*10 mL), dried over Na_2SO_4, concentrated under vacuum. The residue was purified by column

chromatography (eluting with 0-2% EtOAc in petroleum ether) to afford **5a-5g** in 55-70% isolated yields.

III-2:** Characterization of **5a**-**5g

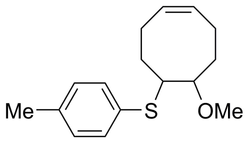

5a ^{1}H NMR (400 MHz, $CDCl_3$) δ: 7.32 (d, J = 8.1 Hz, 2H), 7.08 (d, J = 7.9 Hz, 2H), 5.72-5.61 (m, 1H), 5.58-5.51 (m, 1H), 3.58-3.52 (m, 1H), 3.43-3.39 (m, 1H), 3.38 (s, 3H), 2.38-2.46 (m, 2H), 2.31 (s, 3H), 2.21-2.13 (m, 2H), 2.09-2.02 (m, 2H), 1.85-1.76 (m, 1H), 1.71-1.65 (m, 1H) ppm; ^{13}C NMR (100 MHz, $CDCl_3$) δ: 136.6, 132.4, 132.3, 130.1, 129.5, 128.2, 83.1, 57.8, 52.5, 31.7, 30.2, 24.5, 23.2, 21.0 ppm; FT-IR (KBr, cm^{-1}) 3015, 2925, 2820, 1650, 1450, 1110, 807, 707; HRMS (ESI-TOF): m/z calcd for $C_{16}H_{23}OS$ $[M+H]^+$: 263.1464; found: 263.1458.

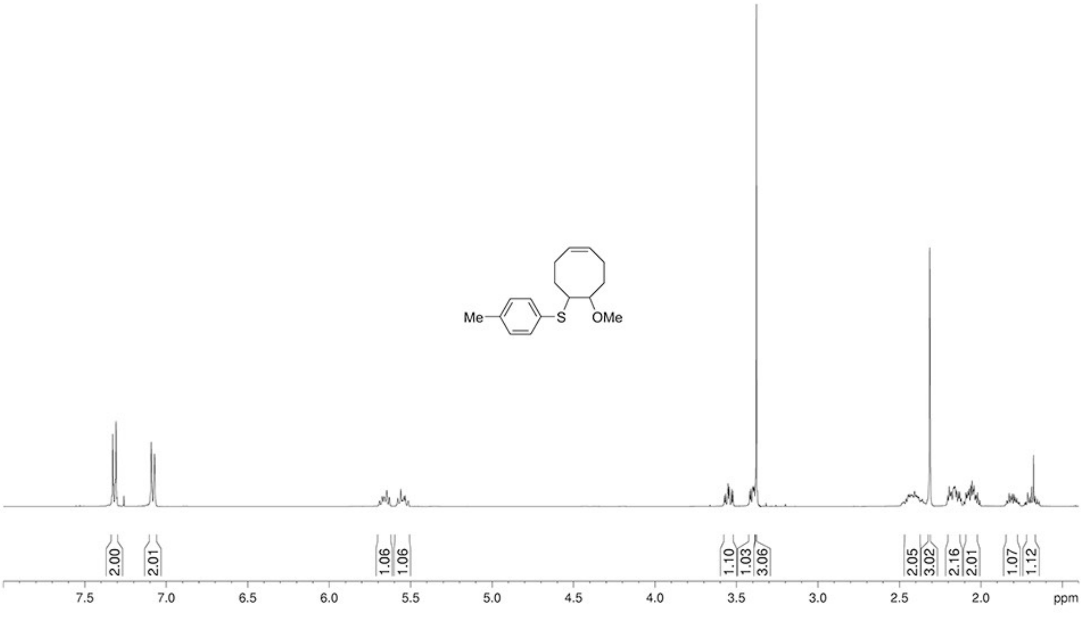

Figure S3. ^{1}H NMR (400 MHz, $CDCl_3$, 25 °C) of Monomer **5a**.

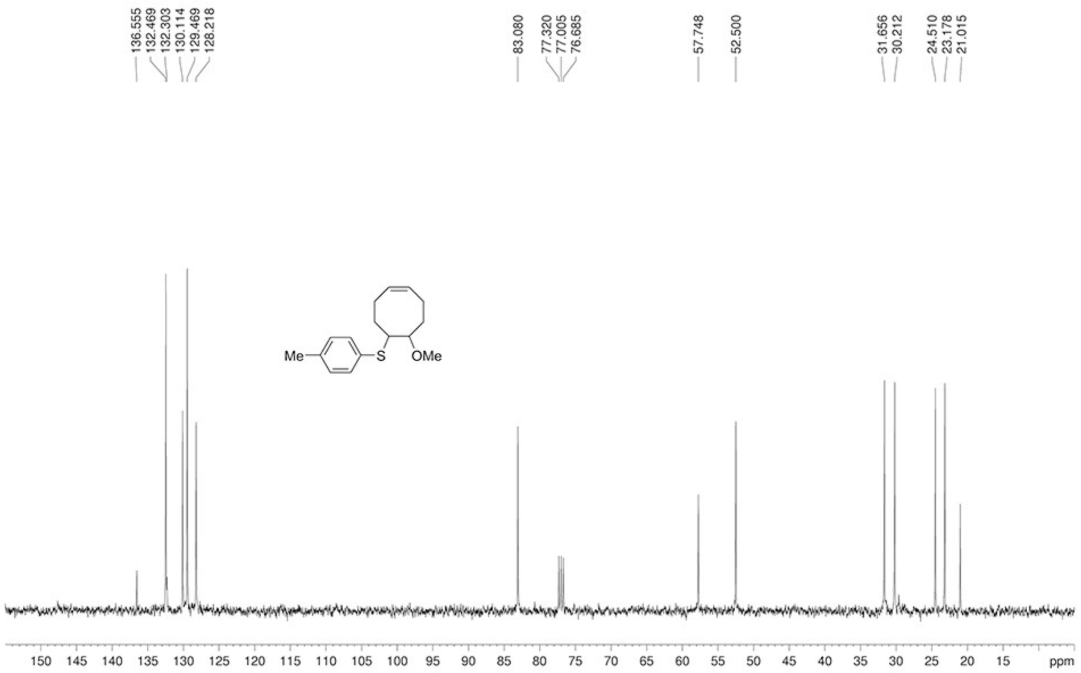

Figure S4.^{13}C NMR (100 MHz, $CDCl_3$, 25 °C) of Monomer **5a**.

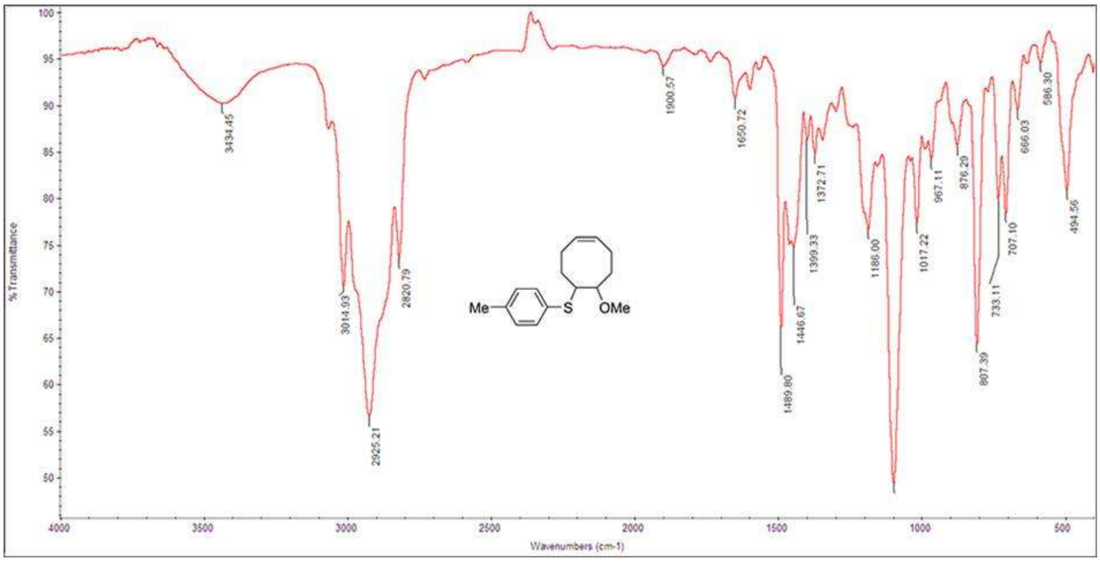

Figure S5. FT-IR spectrum of Monomer **5a**.

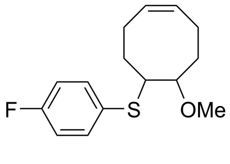

5b ^{1}H NMR (400 MHz, $CDCl_3$) δ: 7.45-7.39 (m, 2H), 7.01-6.97 (m, 2H), 5.71-5.64 (m, 1H), 5.59-5.51 (m, 1H), 3.54-3.49 (m, 1H), 3.42-3.38 (m, 4H), 2.49-2.37 (m, 2H), 2.23-2.15 (m, 2H), 2.11-2.01 (m, 2H), 1.85-1.77 (m, 1H), 1.72-1.65 (m, 1H) ppm; ^{13}C NMR (100 MHz, $CDCl_3$) δ: 162.1 (d, J = 249.7 Hz), 134.6 (d, J = 7.9 Hz), 131.1, 130.3, 128.1, 115.8 (d, J = 20.0 Hz), 83.3, 57.8, 53.3, 31.7, 30.2, 24.5, 23.3 ppm; FT-IR (KBr, cm^{-1}) 2929, 1588, 1485, 1223, 1095, 829, 632; HRMS (ESI-TOF): m/z calcd for $C_{15}H_{20}FOS$ $[M+H]^+$: 267.1213; found: 267.1208.

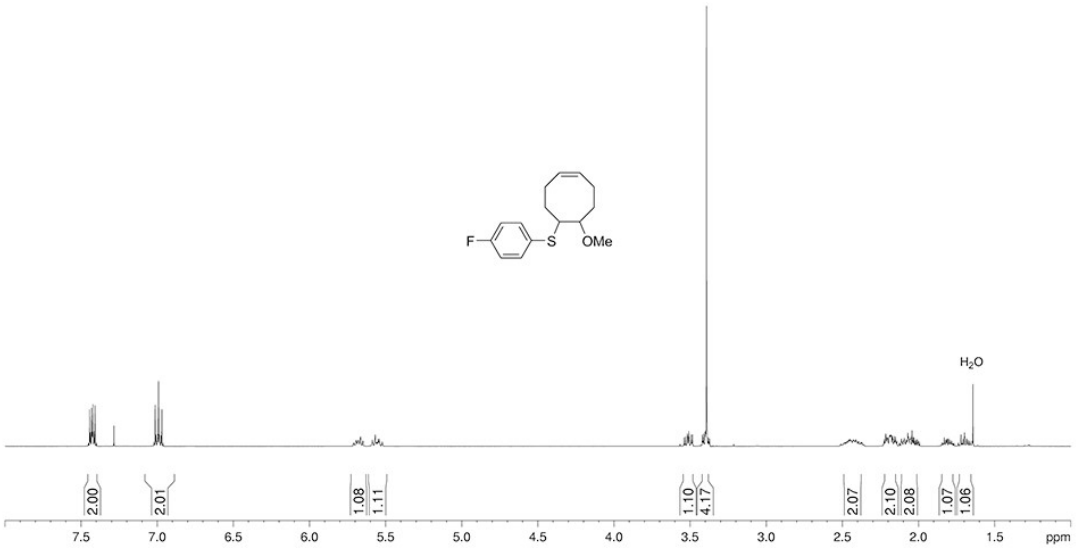

Figure S6. ^{1}H NMR (400 MHz, $CDCl_3$, 25 °C) of Monomer **5b**.

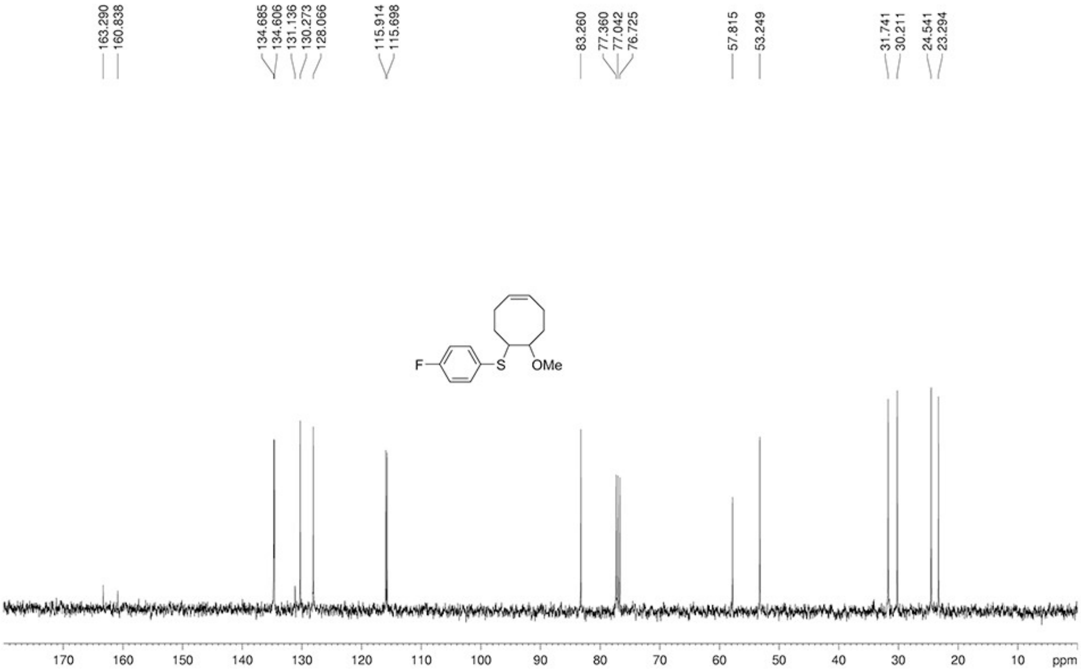

Figure S7.^{13}C NMR (100 MHz, $CDCl_3$, 25 °C) of Monomer **5b**.

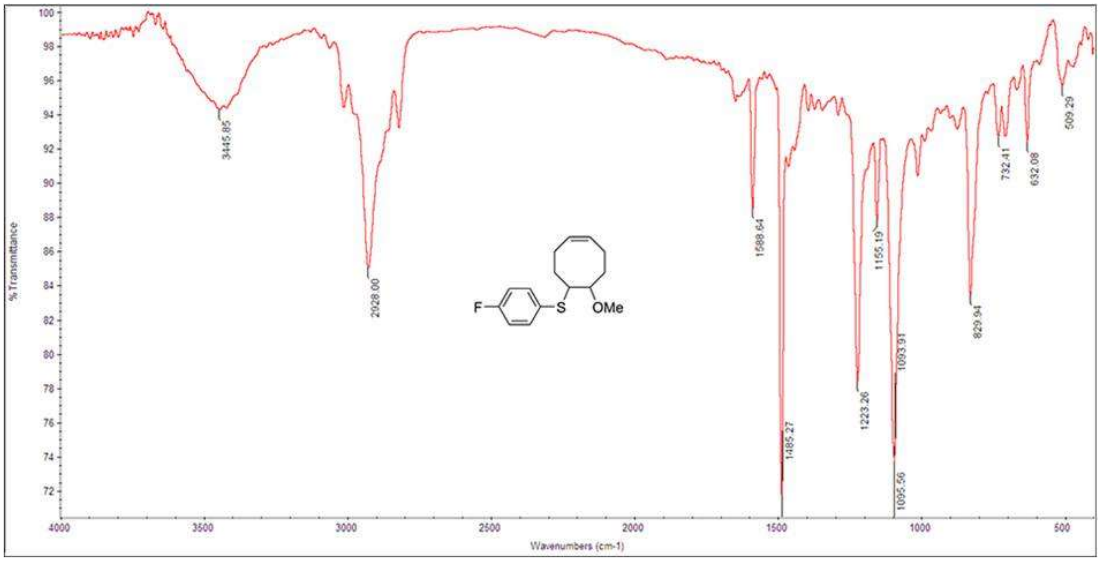

Figure S8. FT-IR spectrum of Monomer **5b**.

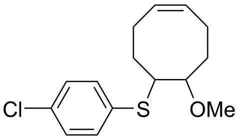

5c ^{1}H NMR (400 MHz, $CDCl_3$) δ: 7.35 (d, J = 8.7 Hz, 2H), 7.25 (d, J = 8.7 Hz, 2H), 5.74-5.64 (m, 1H), 5.61-5.52 (m, 1H), 3.61-3.56 (m, 1H), 3.43-3.37 (m, 4H), 2.52-2.36 (m, 2H), 2.25-2.14 (m, 2H), 2.13-2.02 (m, 2H), 1.86-1.76 (m, 1H), 1.76-1.66 (m, 1H) ppm; ^{13}C NMR (100 MHz, $CDCl_3$) δ: 135.1, 133.0, 132.4, 130.4, 128.9, 128.0, 83.3, 57.9, 52.6, 31.8, 30.3, 24.6, 23.3 ppm; FT-IR (KBr, cm^{-1}) 3014, 2926, 2822, 1650, 1474, 1387, 1096, 818, 737; HRMS (ESI-TOF): m/z calcd for $C_{15}H_{20}ClOS$ $[M+H]^+$: 283.0918; found: 283.0914.

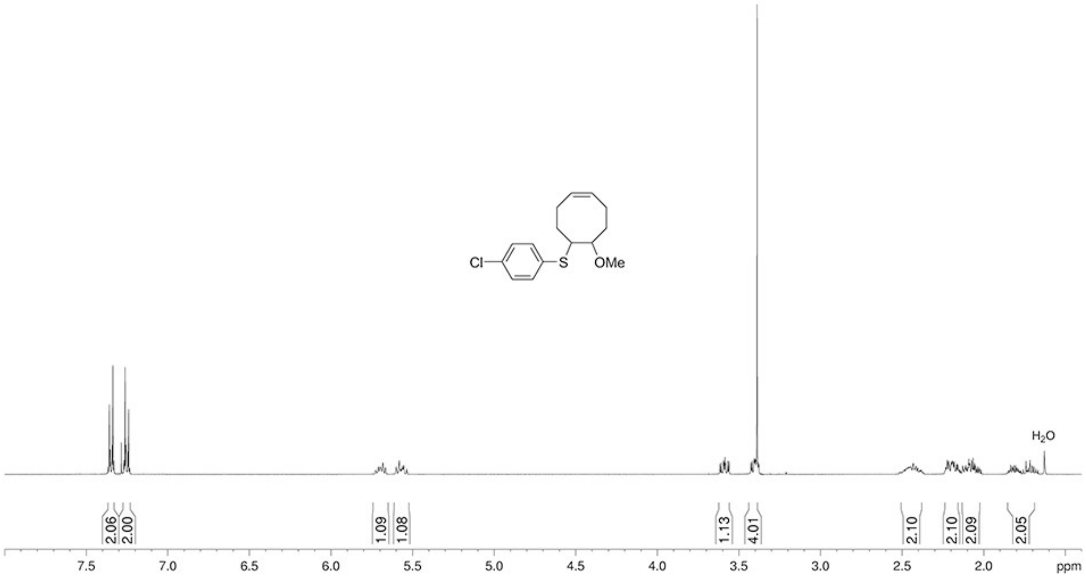

Figure S9. ^{1}H NMR (400 MHz, $CDCl_3$, 25 °C) of Monomer **5c**.

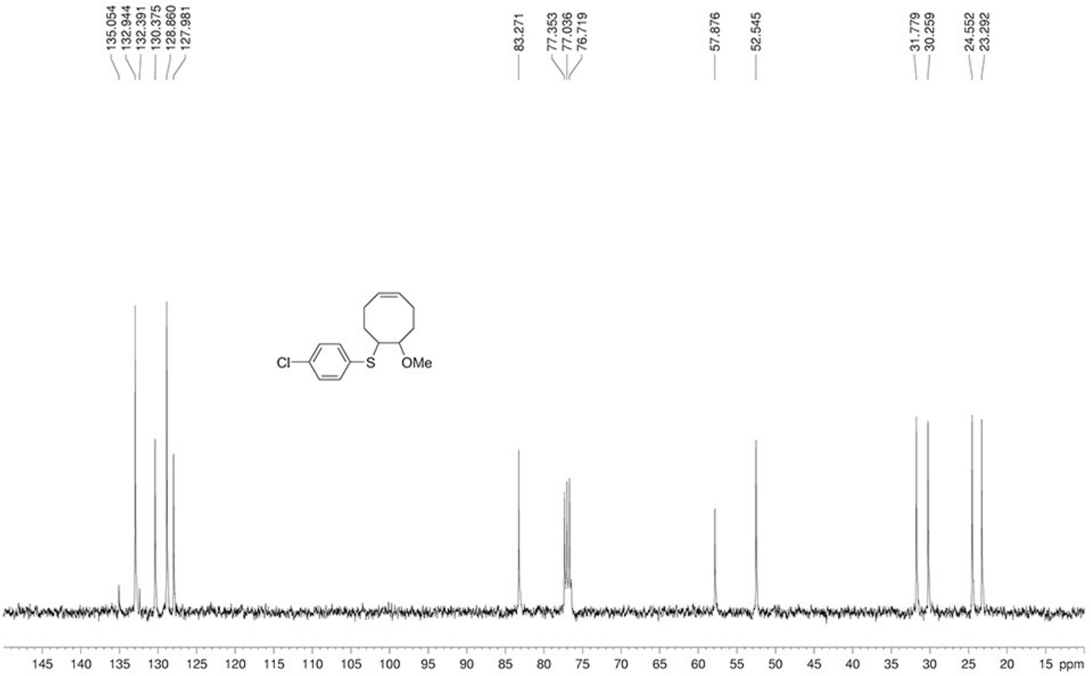

Figure S10. ^{13}C NMR (100 MHz, $CDCl_3$, 25 °C) of Monomer **5c**.

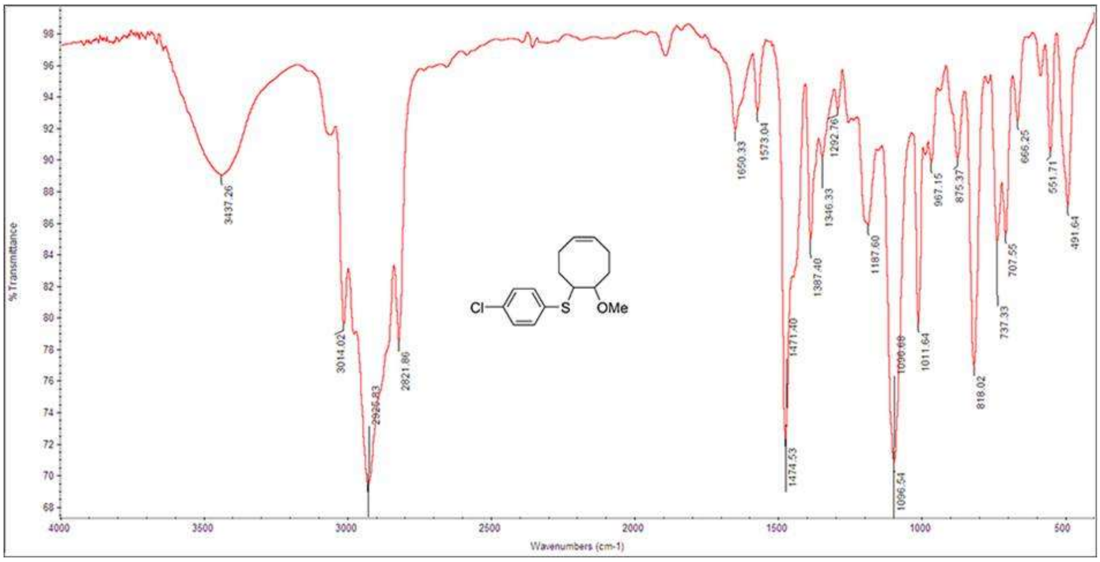

Figure S11. FT-IR spectrum of Monomer **5c**.

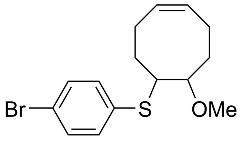

5d ^{1}H NMR (400 MHz, $CDCl_3$) δ: 7.43-7.37 (m, 2H), 7.30-7.26 (m, 2H), 5.73-5.66 (m, 1H), 5.61-5.52 (m, 1H), 3.63-3.57 (m, 1H), 3.43-3.38 (m, 4H), 2.50-2.38 (m, 2H), 2.23-2.15 (m, 2H), 2.12-2.04 (m, 2H), 1.85-1.77 (m, 1H), 1.74-1.67 (m, 1H) ppm; ^{13}C NMR (100 MHz, $CDCl_3$) δ: 135.8, 133.1, 131.8, 130.4, 128.0, 120.3, 83.3, 57.9, 52.4, 31.8, 30.3, 24. 6, 23.3 ppm; FT-IR (KBr, cm^{-1}) 3012, 2925, 2821, 1634, 1470, 1381, 1094, 812; HRMS (ESI-TOF): m/z calcd for $C_{15}H_{20}BrOS$ $[M+H]^+$: 327.0413; found: 327.0408.

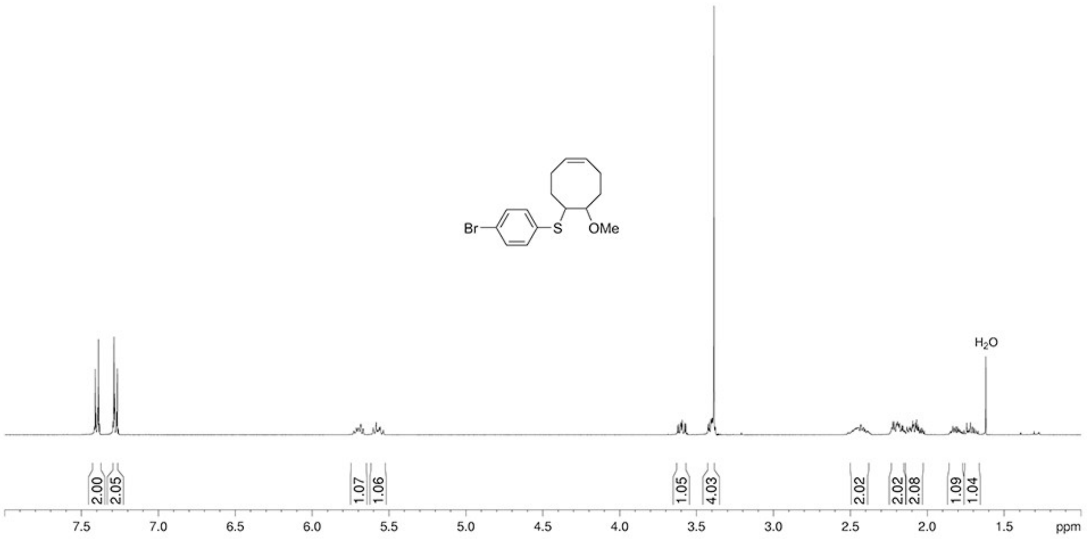

Figure S12.^{1}H NMR (400 MHz, $CDCl_3$, 25 °C) of Monomer **5d**.

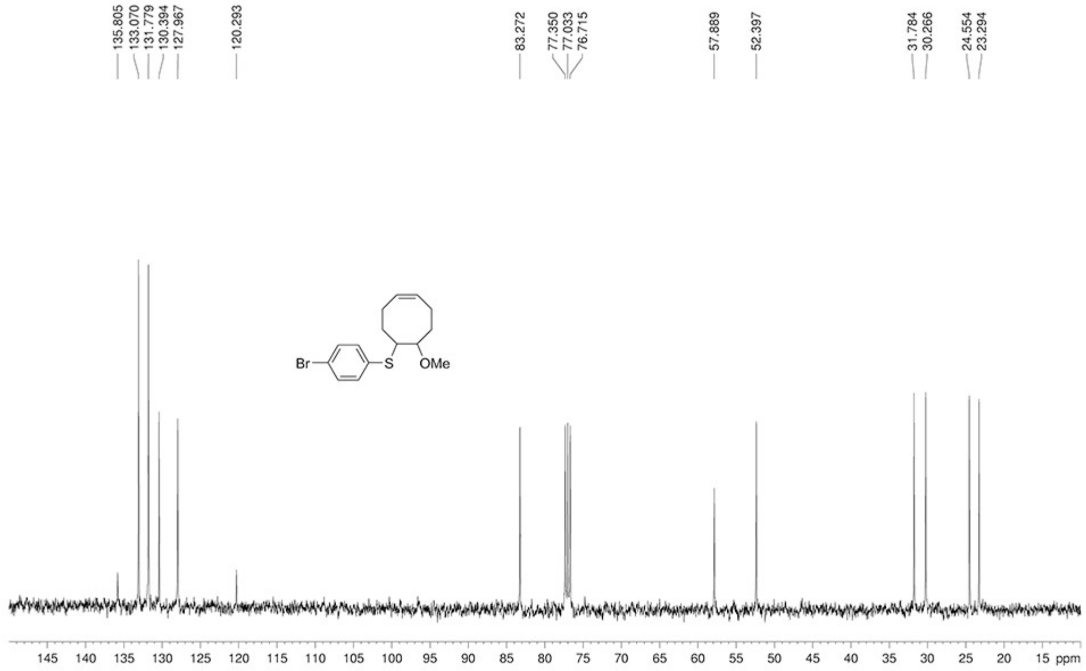

Figure S13.^{13}C NMR (100 MHz, $CDCl_3$, 25 °C) of Monomer **5d**.

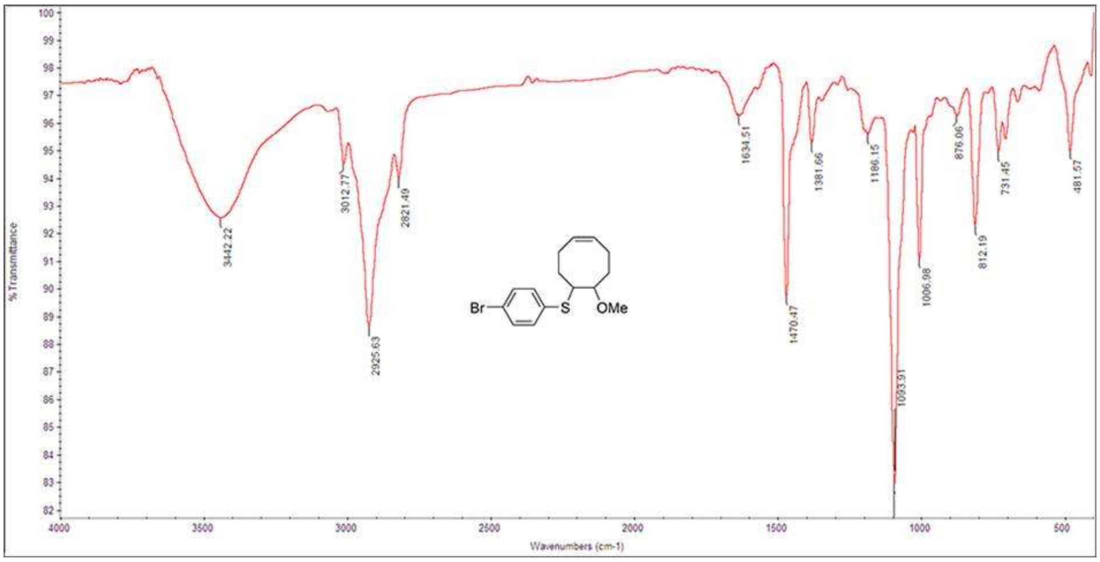

Figure S14. FT-IR spectrum of Monomer **5d**.

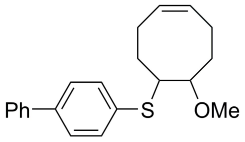

5e ^{1}H NMR (400 MHz, $CDCl_3$) δ: 7.63-7.58 (m, 2H), 7.55-7.43 (m, 6H), 7.39-7.33 (m, 1H), 5.77-5.68 (m, 1H), 5.66-5.58 (m, 1H), 3.73-3.67 (m, 1H), 3.49-3.45 (m, 1H), 3.43 (s, 3H), 2.53-2.43 (m, 2H), 2.25-2.13 (m, 4H), 1.89-1.77 (m, 2H) ppm; ^{13}C NMR (100 MHz, $CDCl_3$) δ: 140.5, 139.2, 135.6, 131.9, 130.3, 128.8, 128.2, 127.4, 127.3, 126.9, 83.2, 57.9, 52.2, 31.9, 30.4, 24.6, 23.3 ppm; FT-IR (KBr, cm^{-1}) 3019, 2927, 2821, 1599, 1478, 1097, 831, 759, 697; HRMS (ESI-TOF): m/z calcd for $C_{21}H_{25}OS$ [M+H]$^+$: 325.1621; found: 325.1620.

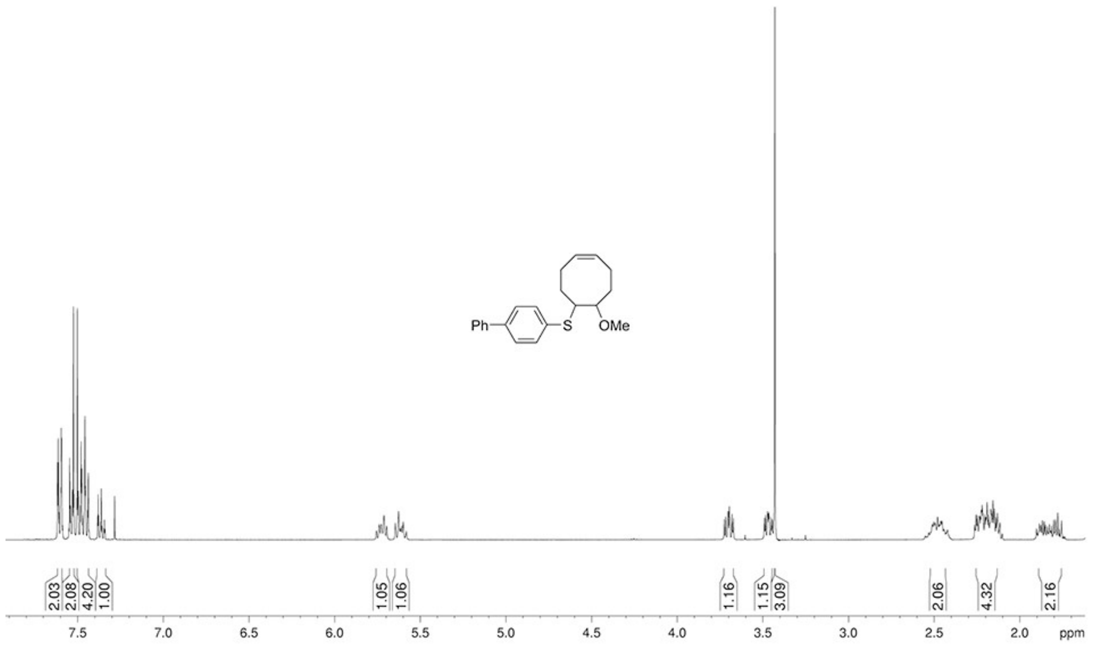

Figure S15. ^{1}H NMR (400 MHz, $CDCl_3$, 25 °C) of Monomer **5e**.

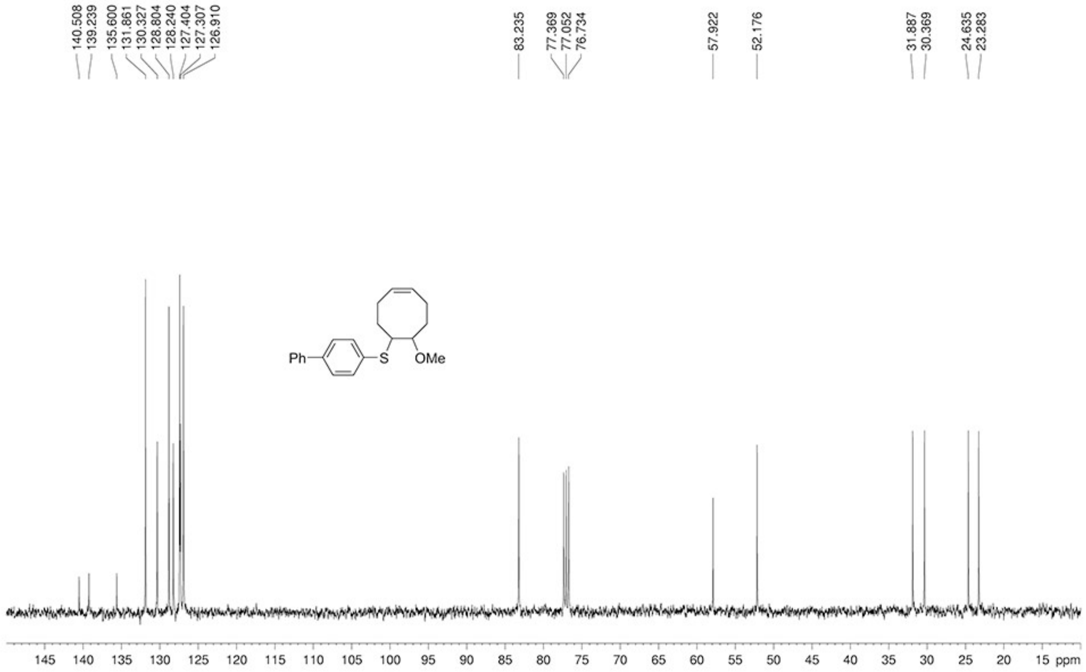

Figure S16.^{13}C NMR (100 MHz, $CDCl_3$, 25 °C) of Monomer **5e**.

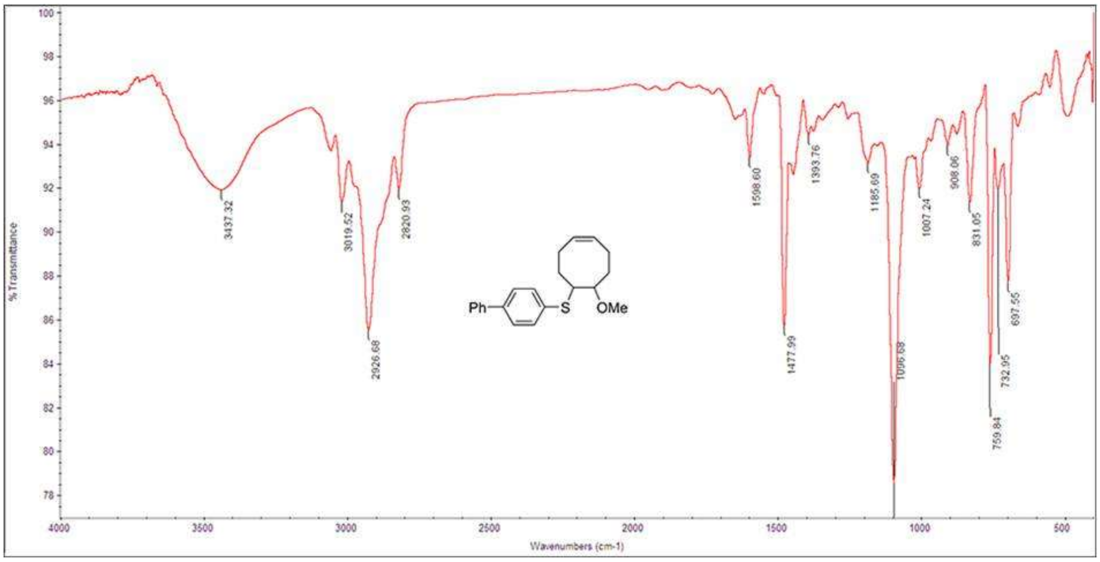

Figure S17. FT-IR spectrum of Monomer **5e**.

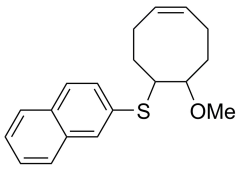

5f ^{1}H NMR (400 MHz, $CDCl_3$) δ: 7.89-7.87 (m, 1H), 7.83-7.74 (m, 3H), 7.54-7.45 (m, 3H), 5.76-5.70 (m, 1H), 5.64-5.56 (m, 1H), 3.81-3.76 (m, 1H), 3.51-3.46 m, 1H), 3.43 (s, 3H), 2.52-2.43 (m, 2H), 2.27-2.18 (m, 2H), 2.16-2.10 (m, 2H), 1.89-1.77 (m, 2H)

ppm; ^{13}C NMR (100 MHz, $CDCl_3$) δ 133.7, 132.0, 130.3, 130.0, 129.5, 128.3, 128.2, 127.7, 127.3, 126.4, 125.8, 124.5, 83.2, 57.9, 52.1, 31.8, 30.4, 24.6, 23.3 ppm; FT-IR (KBr, cm^{-1}) 3012, 2926, 2821, 1623, 1587, 1496, 1459, 1093, 811, 741; HRMS (ESI-TOF): m/z calcd for $C_{19}H_{23}OS$ $[M+H]^+$: 299.1464; found: 299.1465.

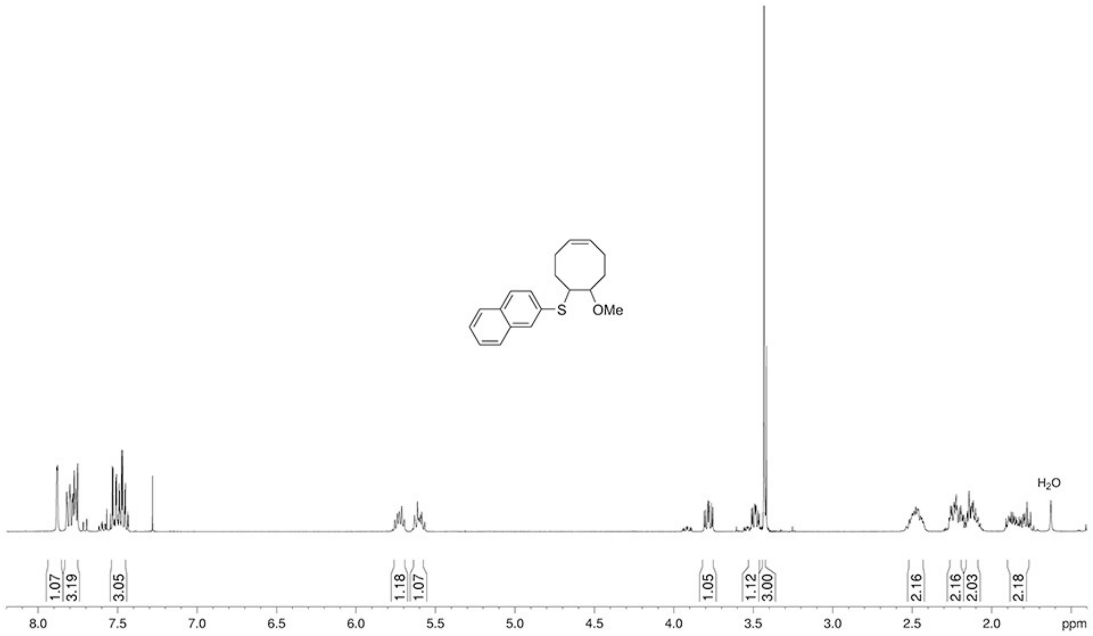

Figure S18.^{1}H NMR (400 MHz, $CDCl_3$, 25 °C) of Monomer **5f**.

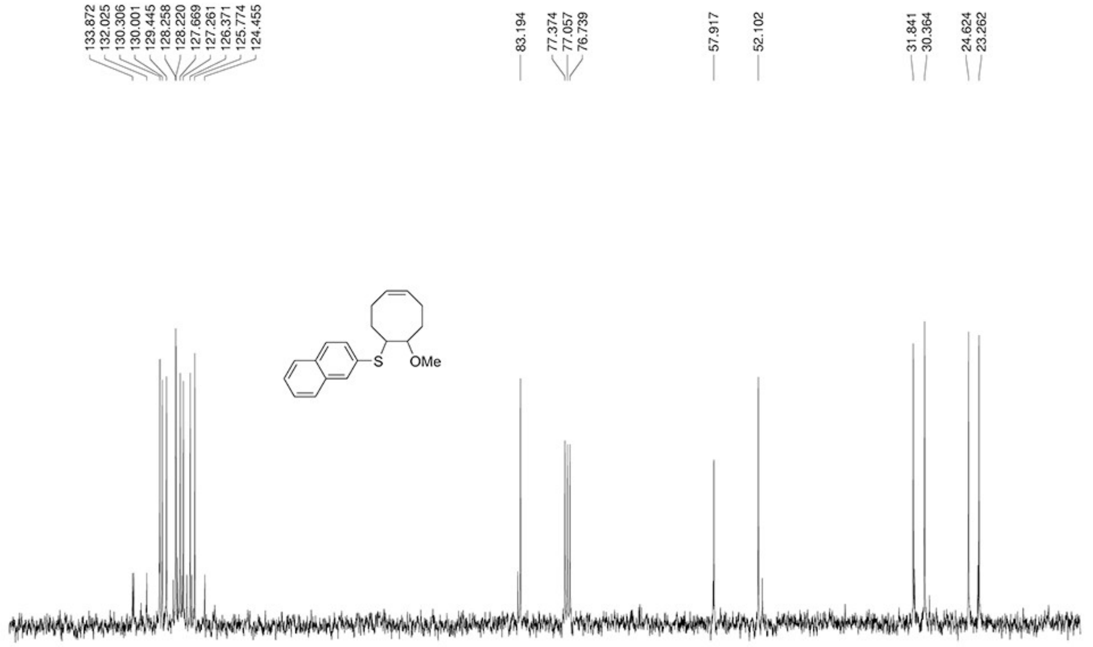

Figure S19. ^{13}C NMR (100 MHz, $CDCl_3$, 25 °C) of Monomer **5f**.

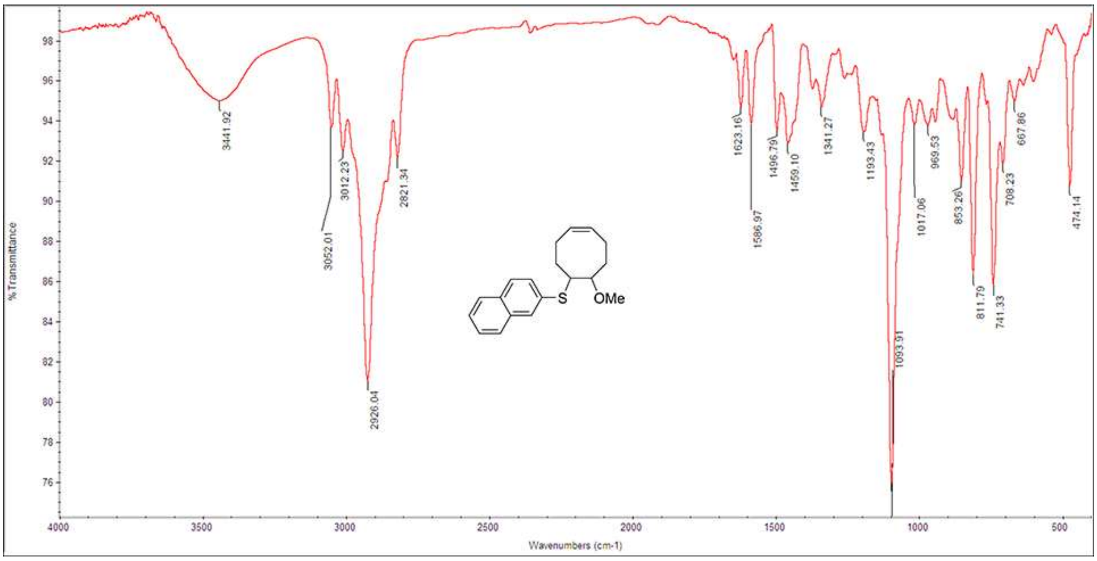

Figure S20. FT-IR spectrum of Monomer **5f**.

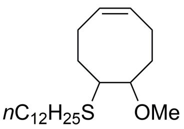

5g ^{1}H NMR (400 MHz, $CDCl_3$) δ: 5.70-5.64 (m, 1H), 5.61-5.55 (m, H), 3.38 (s, 3H), 3.37-3.32 (m, 1H),3.12-3.07 (m, 1H), 2.60-2.56 (m, 2H), 2.50-2.31 (m, 2H), 2.21-2.09 (m, 4H), 1.79-1.68 (m, 2H), 1.61-1.54 (m, 2H), 1.40-1.21 (m, 18H), 0.89 (t, J = 6.9 Hz, 3H) ppm; ^{13}C NMR (100 MHz, $CDCl_3$) δ: 130.3, 128.2, 84.3, 57.8, 49.0, 32.5, 31.9, 31.9, 30.5, 29.8, 29.6, 29.6, 29.5, 29.3, 29.3, 29.1, 24.7, 23.3, 22.7, 14.1 ppm; FT-IR (KBr, cm^{-1}) 2930, 285, 1462, 1099, 469; HRMS (ESI-TOF): m/z calcd for $C_{21}H_{41}OS$ $[M+H]^{+}$: 341.2873; found: 341.2868.

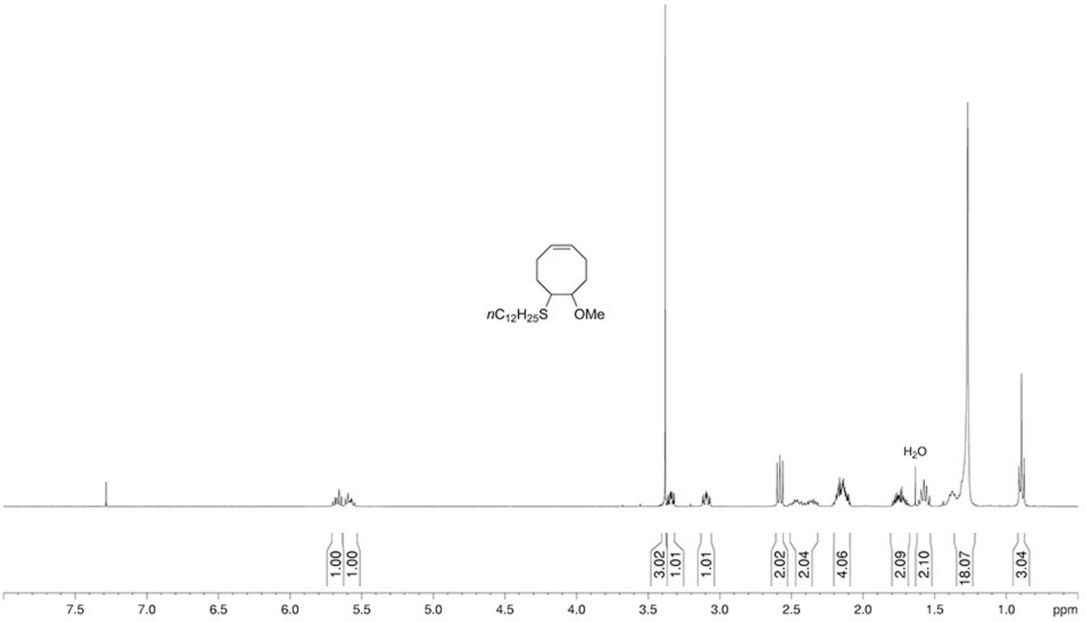

Figure S21. ^{1}H NMR (400 MHz, $CDCl_3$, 25 °C) of Monomer **5g**.

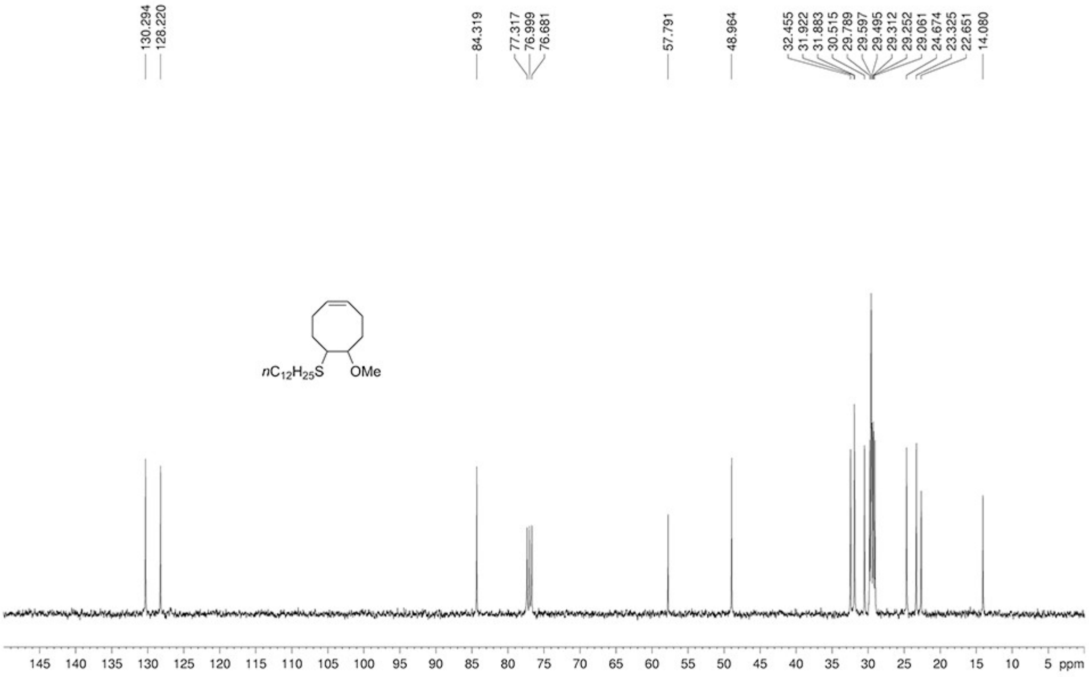

Figure S22.^{13}C NMR (100 MHz, $CDCl_3$, 25 °C) of Monomer **5g**.

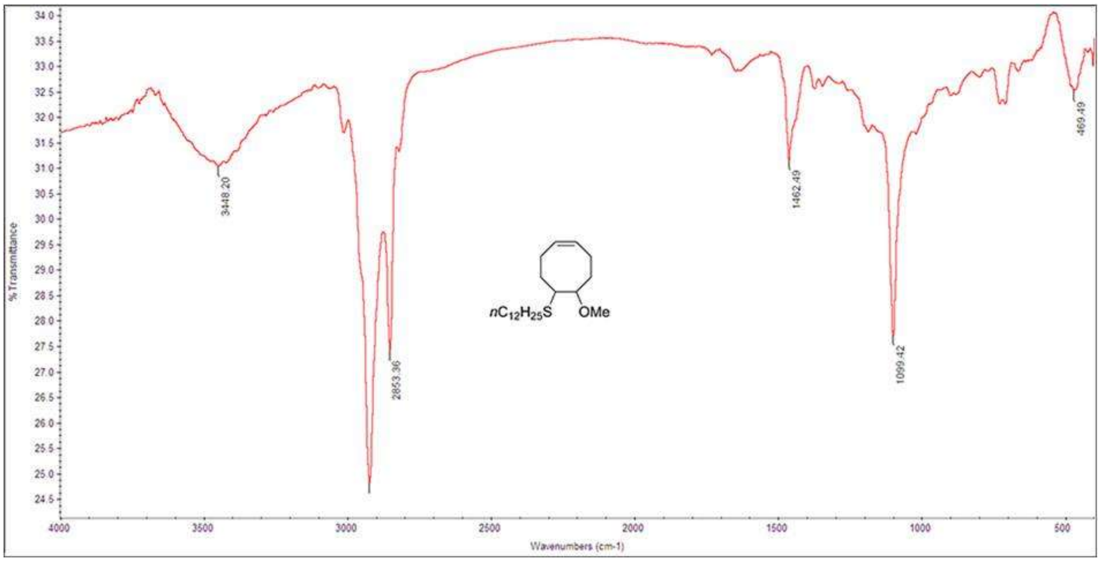

Figure S23. FT-IR spectrum of Monomer **5g**.

Section IV**: Synthesis and Characterization of Polymers **6a**-**6g

IV-1:** General procedure for the synthesis of **6a-6g

An oven-dried vial equipped a stir bar was charged with 1.0 mL solution of **5** (0.5 M) in anhydrous DCM under N_2. The G2 (Grubbs second-generation) catalyst solution (8.5 mg/mL in degassed DCM) was added via a micro syringe into the vial at room temperature in a corresponding volume (50 µL-125 µL). After stirring for 24-48 h, the mixture was concentrated and was dropwisely added into MeOH with vigorously stirring. Solid compound was collected and re-dissolved in minimal amount of DCM. The precipitation procedure was repeated for three times in total. The combined organic layer was concentrated. The resulted residue was purified by column chromatography to recovery the unreacted monomer and calculate the monomer conversion. The produced polymer was characterized with ^{1}H NMR, ^{13}C NMR, FT-IR, GPC, DSC and TGA analysis.

IV-2:** Characterization of **6a**-**6g

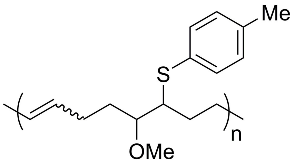

6a ^{1}H NMR (400 MHz, $CDCl_3$) δ: 7.36-7.30 (m, 2H), 7.15-7.04 (m, 2H), 5.47-5.31 (m, 2H), 3.31-.3.15 (m, 5H), 2.37-2.28 (m, 4H), 2.23-2.06 (m, 2H), 2.05-1.90 (m,1H), 1.89-

1.75 (m, 2H), 1.57-1.39 (m, 2H) ppm; FT-IR (KBr, cm^{-1}) 2926, 1635, 1491, 1446, 1091, 806, 474.

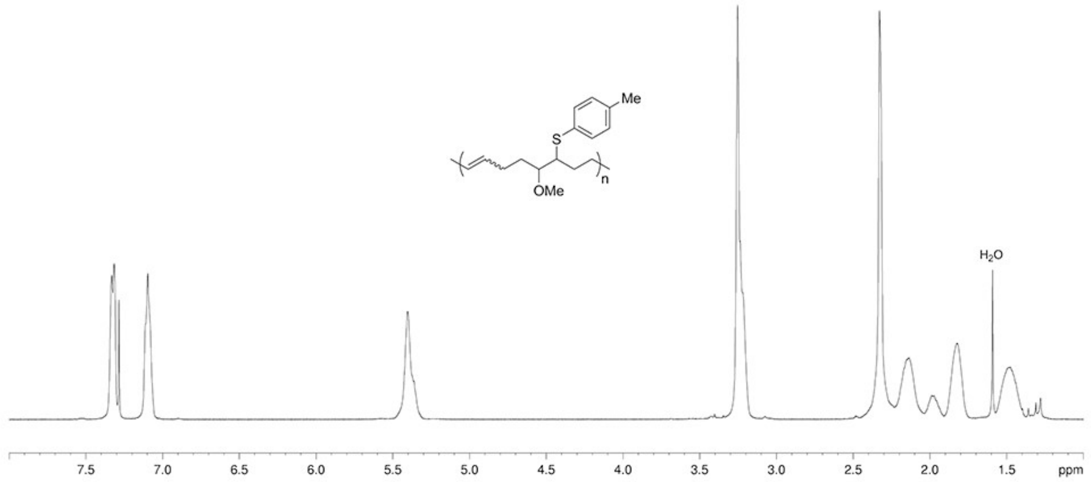

Figure S24. ^{1}H NMR (400 MHz, $CDCl_3$, 25 °C) of Polymer **6a**.

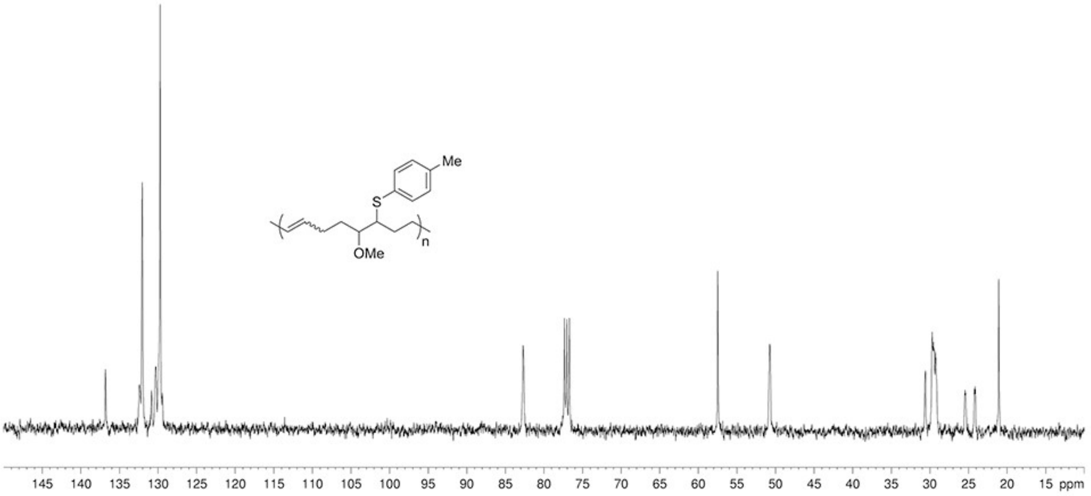

Figure S25. ^{13}C NMR (100 MHz, $CDCl_3$, 25 °C) of Polymer **6a**.

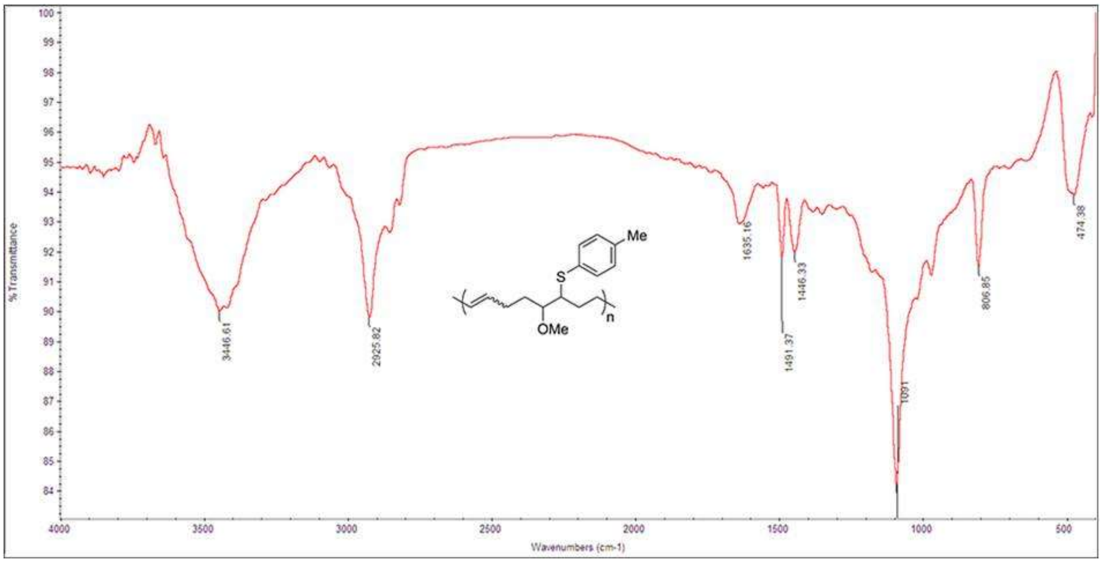

Figure S26. FT-IR spectrum of Polymer **6a**.

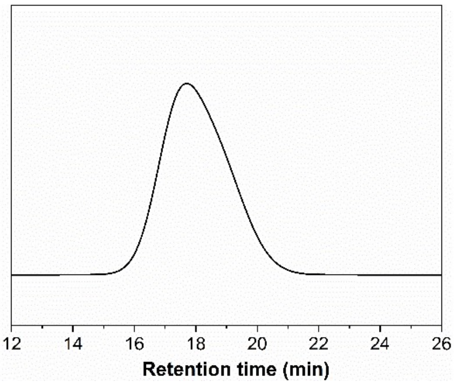

Figure S27. GPC Characterization of Polymer **6a**.

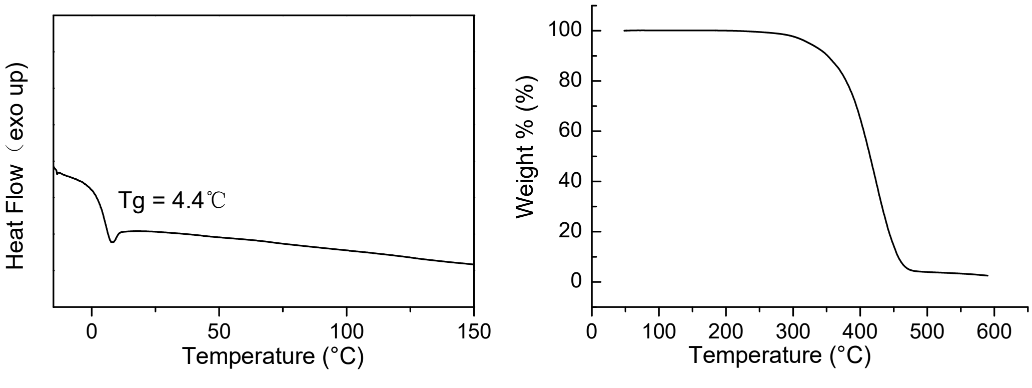

Figure S28. DSC and TGA Characterization of Polymer **6a**.

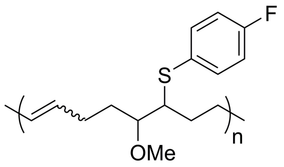

6b ^{1}H NMR (400 MHz, $CDCl_3$) δ:7.50-7.36 (m, 2H), 7.10-6.90 (m, 2H), 5.48-5.30 (m, 2H), 3.31-3,13 (m, 5H), 2.40-1.91 (m, 4H), 1.90-1.72 (m, 2H), 1.60-1.37 (m, 2H) ppm; FT-IR (KBr, cm^{-1}) 2928, 1589, 1488, 1448, 1225, 1091, 830, 634.

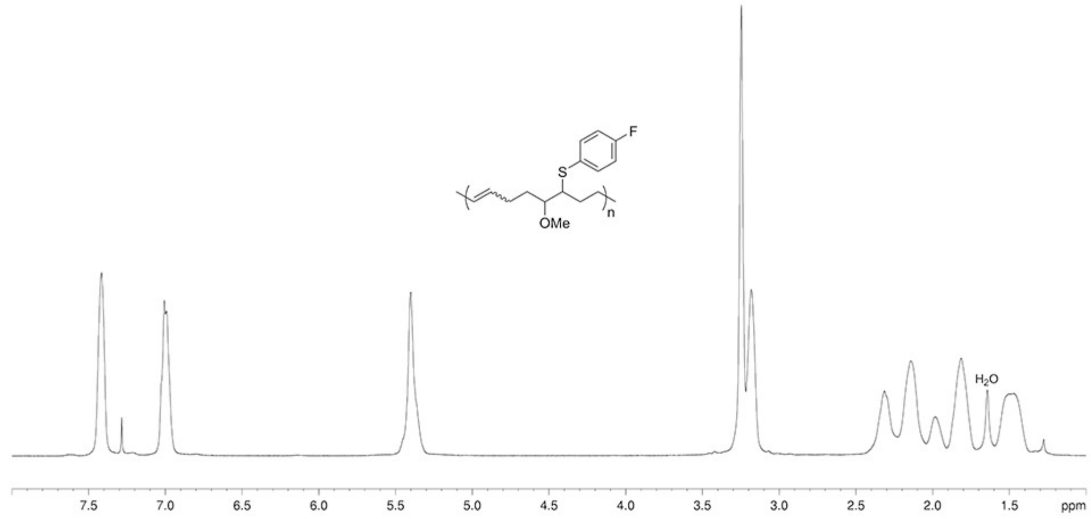

Figure S29. ^{1}H NMR (400 MHz, $CDCl_3$, 25 °C) of Polymer **6b**.

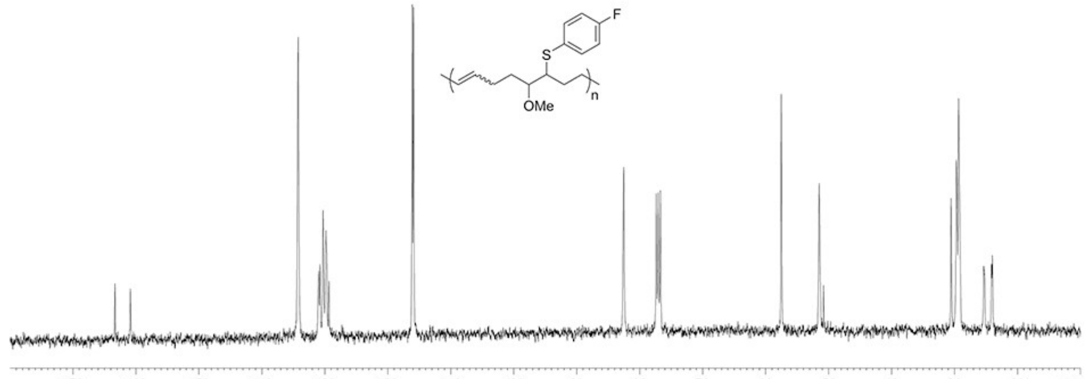

Figure S30. ^{13}C NMR (100 MHz, $CDCl_3$, 25 °C) of Polymer **6b**.

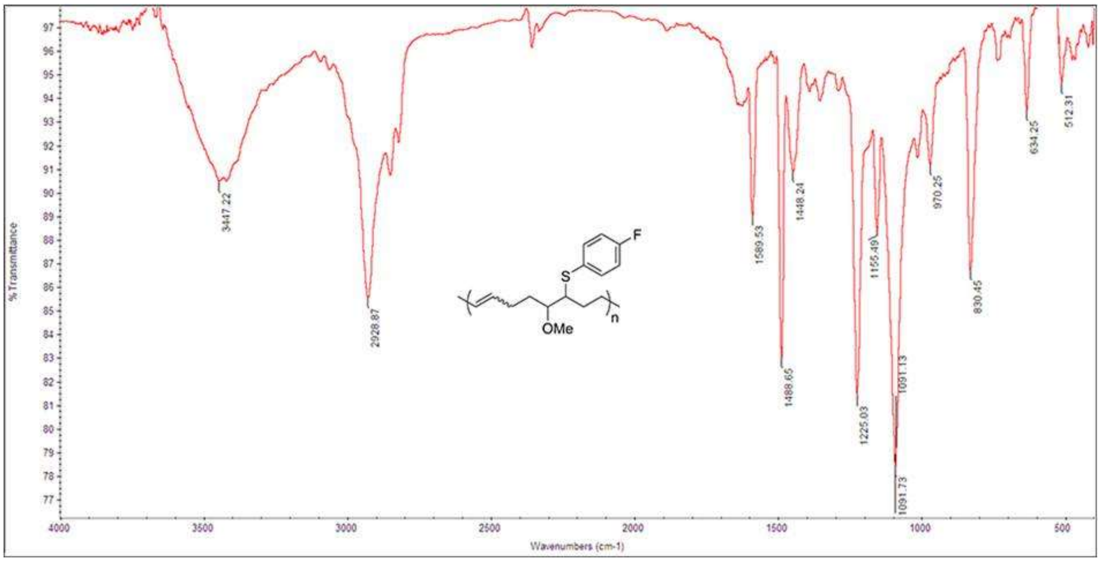

Figure S31. FT-IR spectrum of Polymer **6b**.

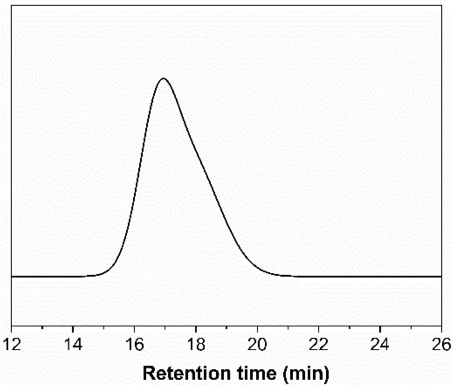

Figure S32. GPC Characterization of Polymer **6b**.

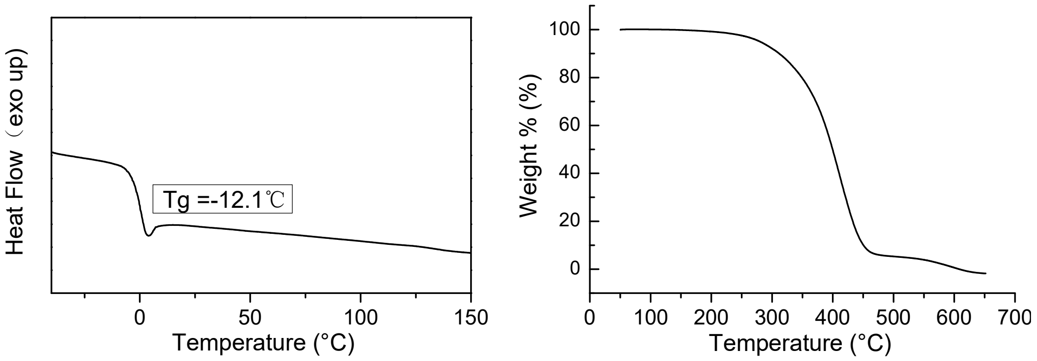

Figure S33. DSC and TGA Characterization of Polymer **6b**.

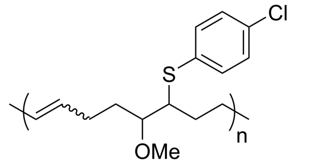

6c ^{1}H NMR (400 MHz, $CDCl_3$) δ:7.42-7.13 (m, 4H), 5.48-5.28 (m, 2H), 3.40-3.06 (m, 5H), 2.40-1.90 (m, 4H), 1.89-1.69 (m, 2H), 1.60-1.36 (m, 2H) ppm; FT-IR (KBr, cm^{-1}) 2928, 1636, 1474, 1445, 1387, 1097, 819, 735.

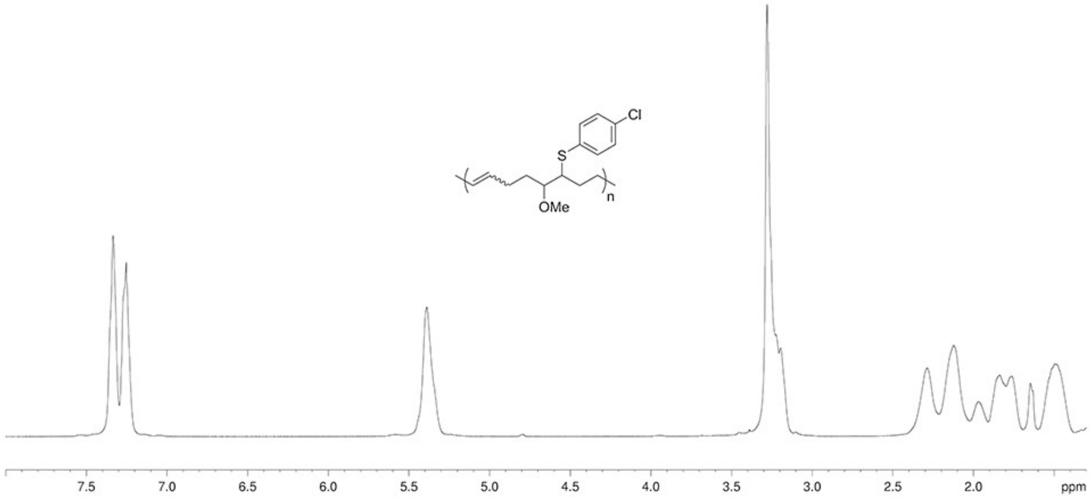

Figure S34. ^{1}H NMR (400 MHz, $CDCl_3$, 25 °C) of Polymer **6c**.

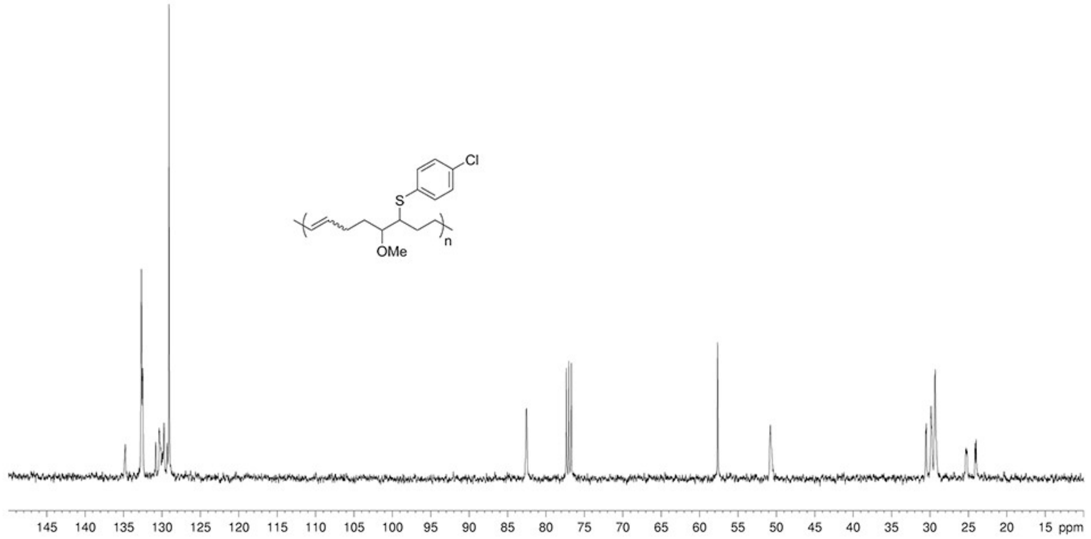

Figure S35. ^{13}C NMR (100 MHz, $CDCl_3$, 25 °C) of Polymer **6c**.

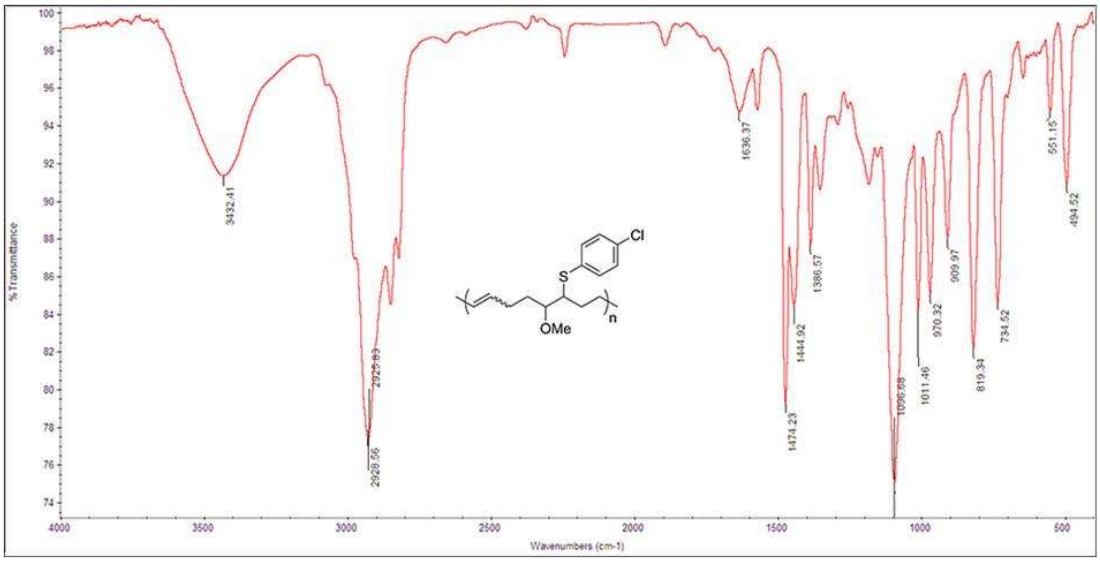

Figure S36. FT-IR spectrum of Polymer **6c**.

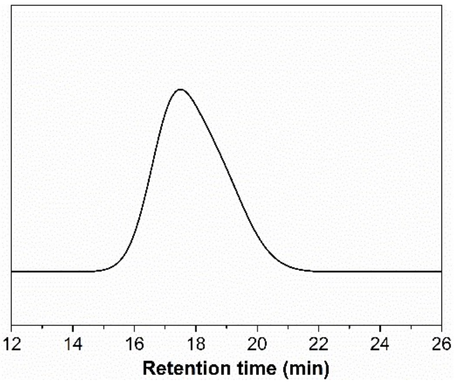

Figure S37. GPC Characterization of Polymer **6c**.

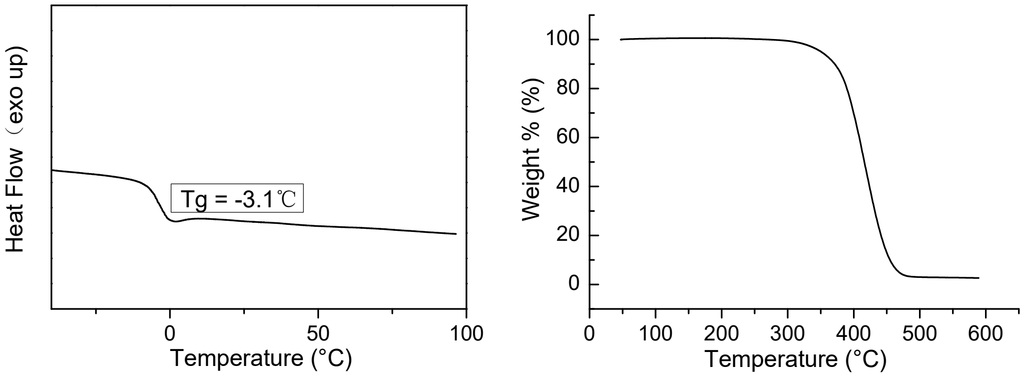

Figure S38. DSC and TGA Characterization of Polymer **6c**.

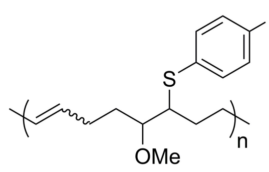

6d ^{1}H NMR (400 MHz, $CDCl_3$) δ: 7.46-7.34 (m, 2H), 7.33-7.22 (m, 2H), 5.50-5.27 (m, 2H), 3.50-3.01 (m, 5H), 2.36-2.91 (m, 4H), 1.91-1.70 (m, 2H), 1.60-1.39 (m, 2H) ppm; FT-IR (KBr, cm^{-1}) 2927, 1471, 1384, 1091, 813, 730.

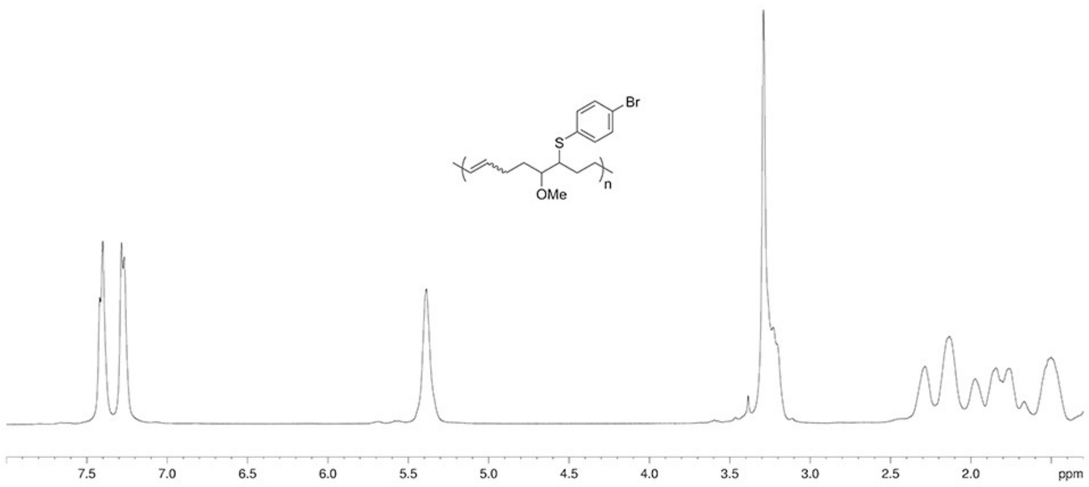

Figure S39. ^{1}H NMR (400 MHz, $CDCl_3$, 25 °C) of Polymer **6d**.

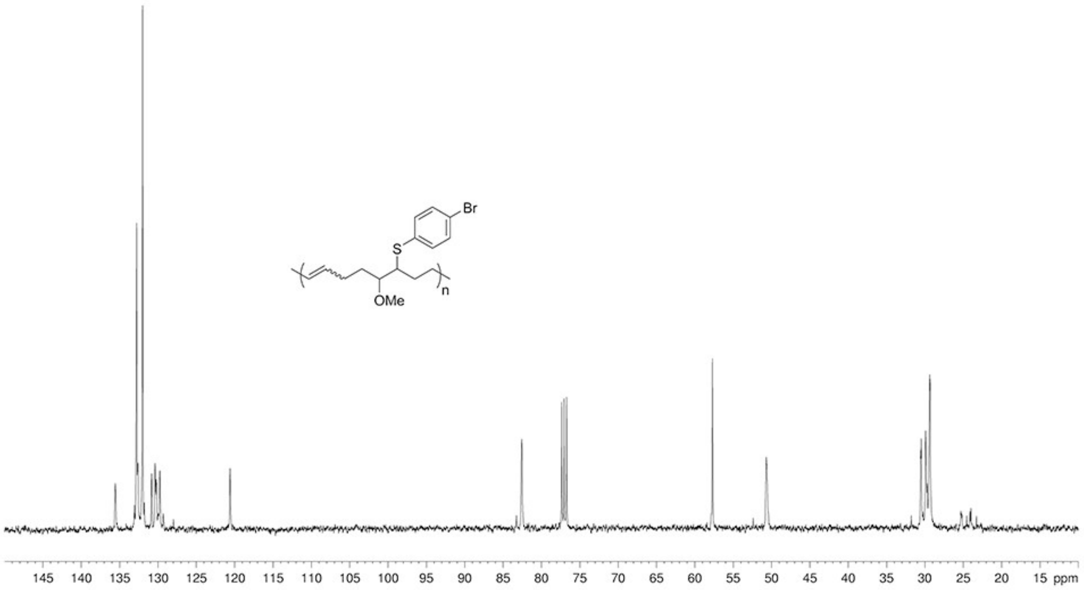

Figure S40. ^{13}C NMR (100 MHz, $CDCl_3$, 25 °C) of Polymer **6d**.

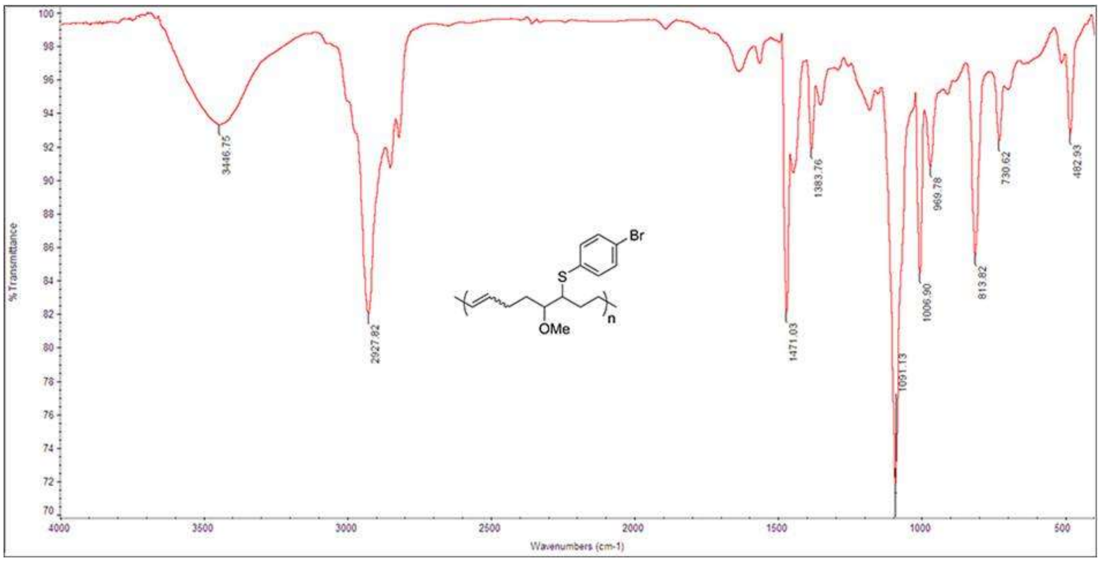

Figure S41. FT-IR spectrum of Polymer **6d**.

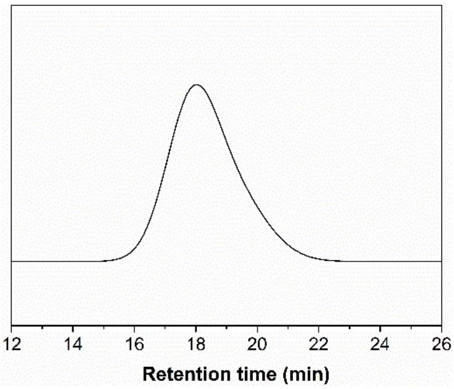

Figure S42. GPC Characterization of Polymer **6d**.

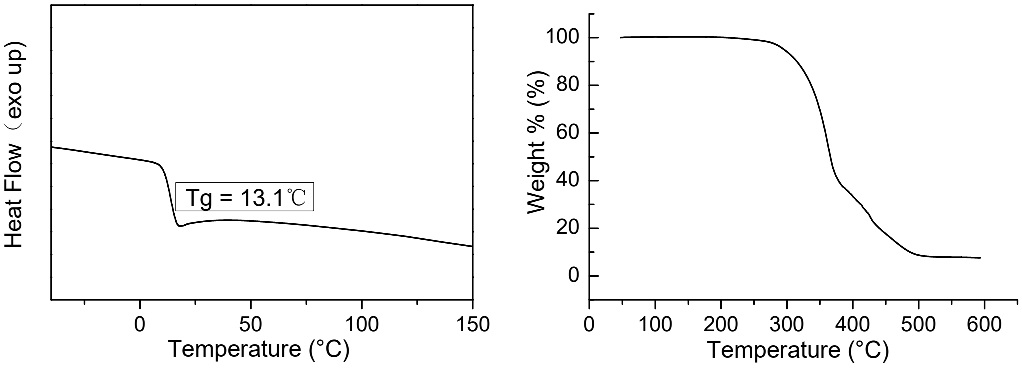

Figure S43. DSC and TGA Characterization of Polymer **6d**.

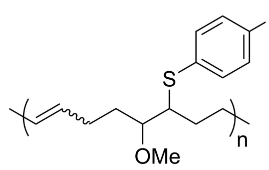

6e ^{1}H NMR (400 MHz, $CDCl_3$) δ: 7.63-7.30 (m, 9H), 5.54-5.26 (m, 2H), 3.43-3.19 (m, 5H), 2.49-2.13 (m, 6H), 1.59-1.42 (m,2H) ppm; FT-IR (KBr, cm^{-1}) 3025, 2928, 2852, 1599, 1479, 1446, 1093, 832, 760;

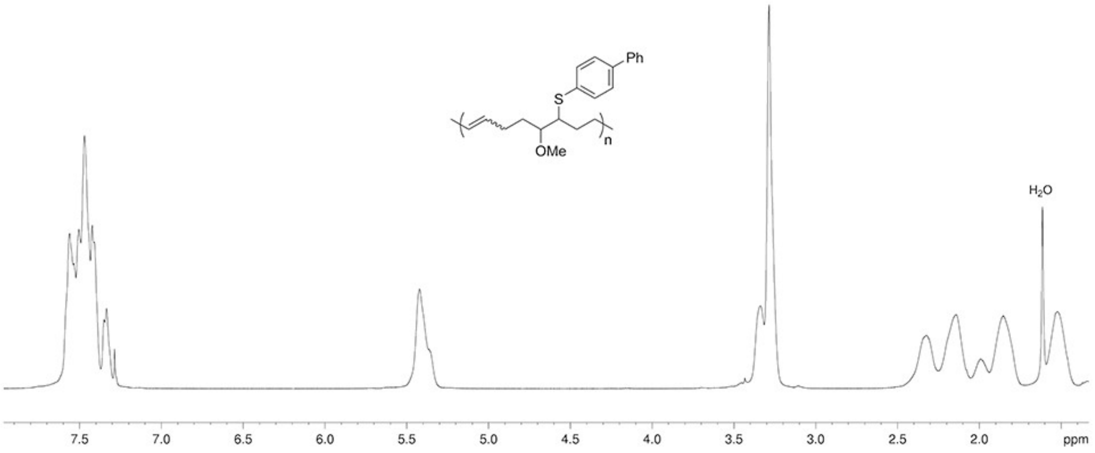

Figure S44. ^{1}H NMR (400 MHz, $CDCl_3$, 25 °C) of Polymer **6e**.

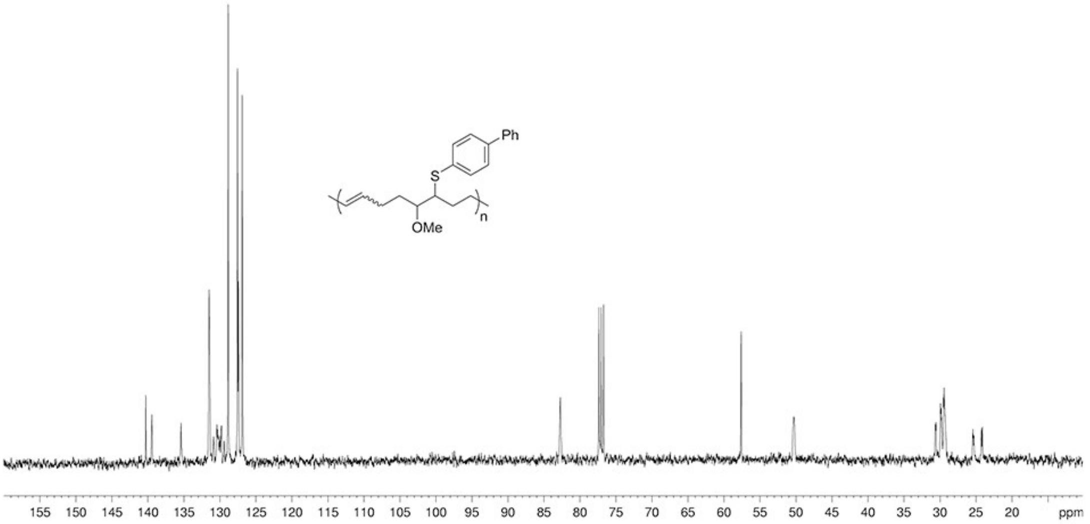

Figure S45. ^{13}C NMR (100 MHz, $CDCl_3$, 25 °C) of Polymer **6e**.

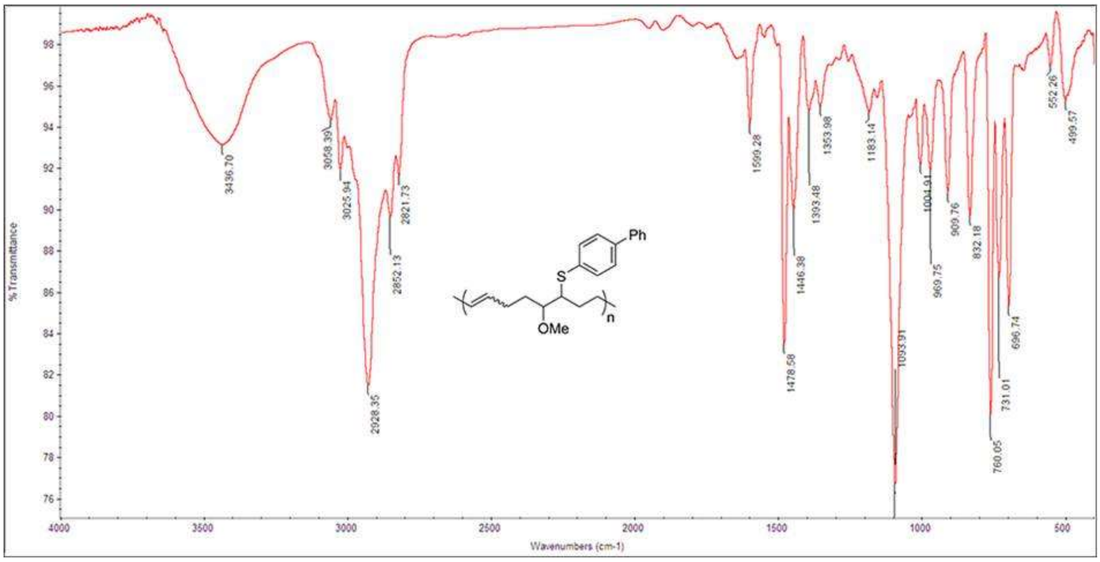

Figure S46. FT-IR spectrum of Polymer **6e**.

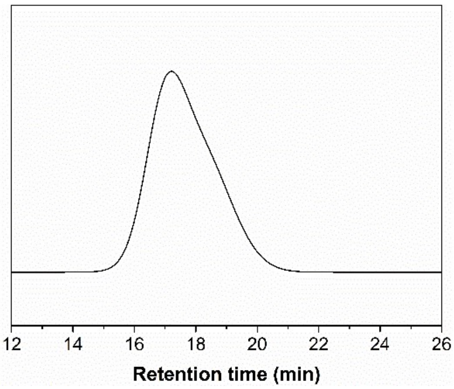

Figure S47. GPC Characterization of Polymer **6e**.

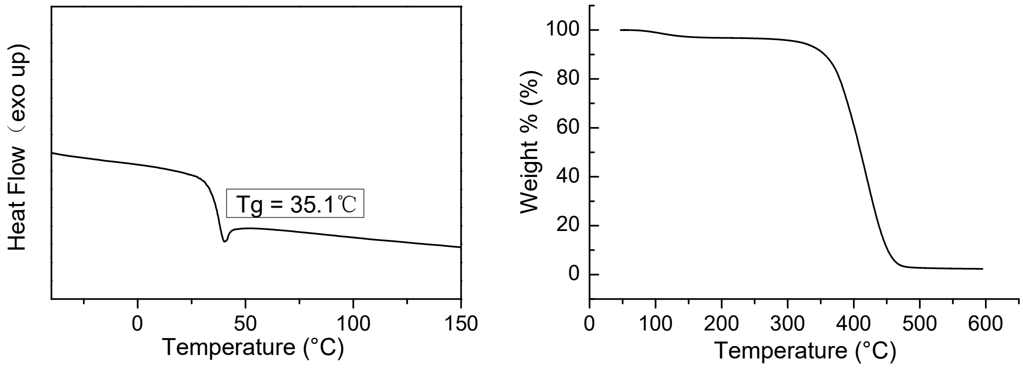

Figure S48. DSC and TGA Characterization of Polymer **6e**.

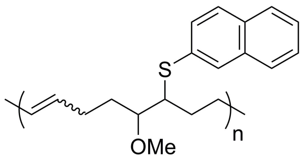

6f ^{1}H NMR (400 MHz, $CDCl_3$) δ: 7.79-7.60 (m, 4H), 7.59-7.35 (m,3H), 5.50-5.20 (m, 2H),3.60-3.40 (m, 1H), 3.33-3.13 (m, 4H), 2.50-1.70 (m, 6H), 1.59-1.39 (m, 2H) ppm; FT-IR (KBr, cm^{-1}) 3052, 2929, 2853, 1623, 1587, 1448, 1091, 812, 737.

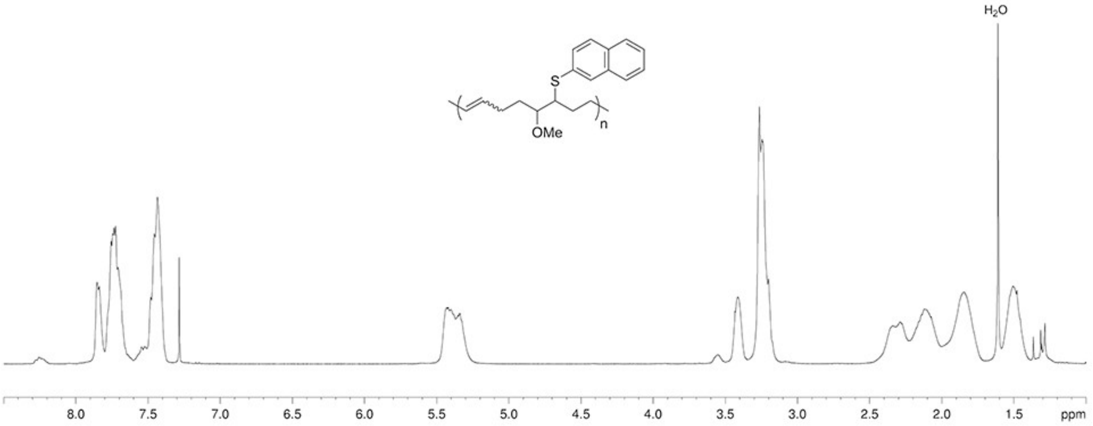

Figure S49. ^{1}H NMR (400 MHz, $CDCl_3$, 25 °C) of Polymer **6f**.

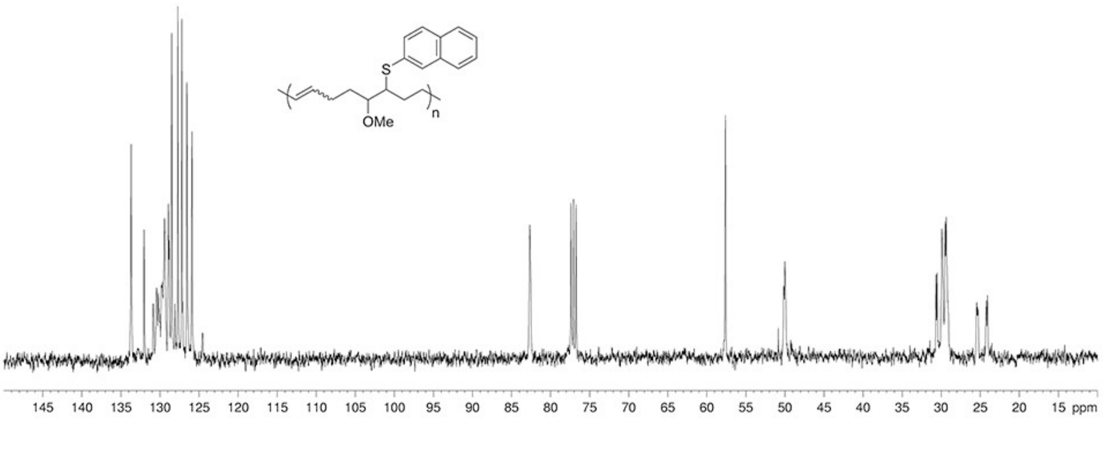

Figure S50. ^{13}C NMR (100 MHz, $CDCl_3$, 25 °C) of Polymer **6f**.

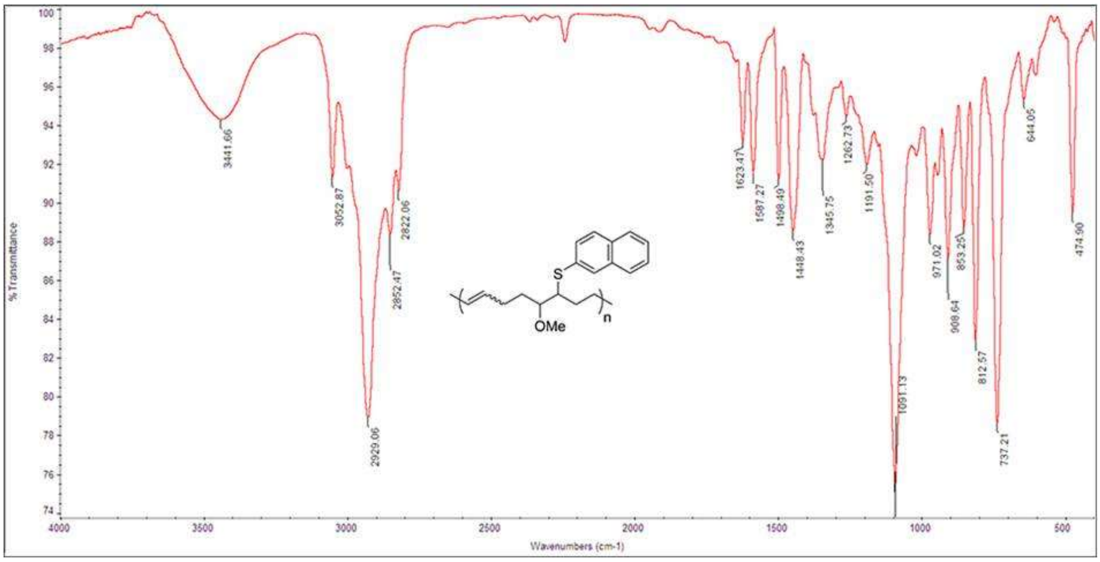

Figure S51. FT-IR spectrum of Polymer **6f**.

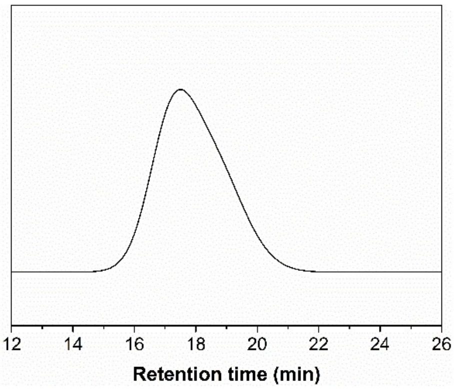

Figure S52. GPC Characterization of Polymer **6f**.

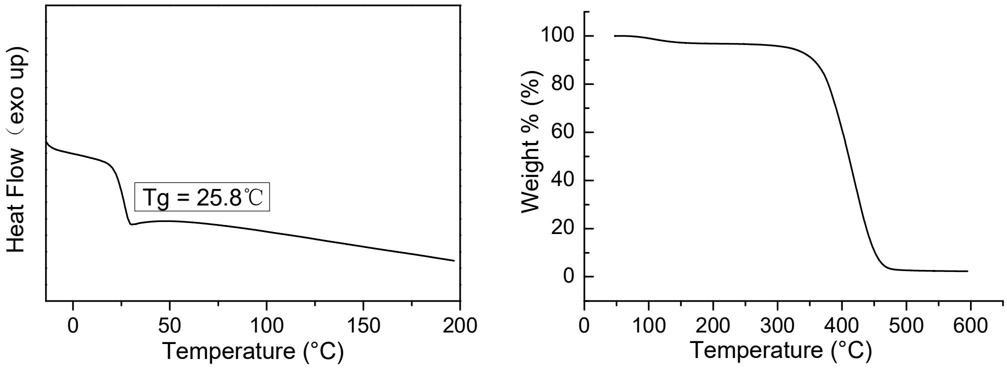

Figure S53. DSC and TGA Characterization of Polymer **6f**.

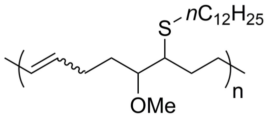

6g ^{1}H NMR (400 MHz, $CDCl_3$) δ: 5.60-5.30 (m, 2H), 3.45-3.31 (m, 3H), 3.30-3.20 (m,1H), 2.83-2.67 (m, 1H), 2.61-2,45 (m, 2H), 2.40-1.95 (m, 4H), 1.85-1.68 (m, 2H), 1.64-1.15 (m, 22H), 0.97-0.8 (m, 3H) ppm; FT-IR (KBr, cm^{-1}) 2923, 2853, 1626, 1460, 1095, 719.

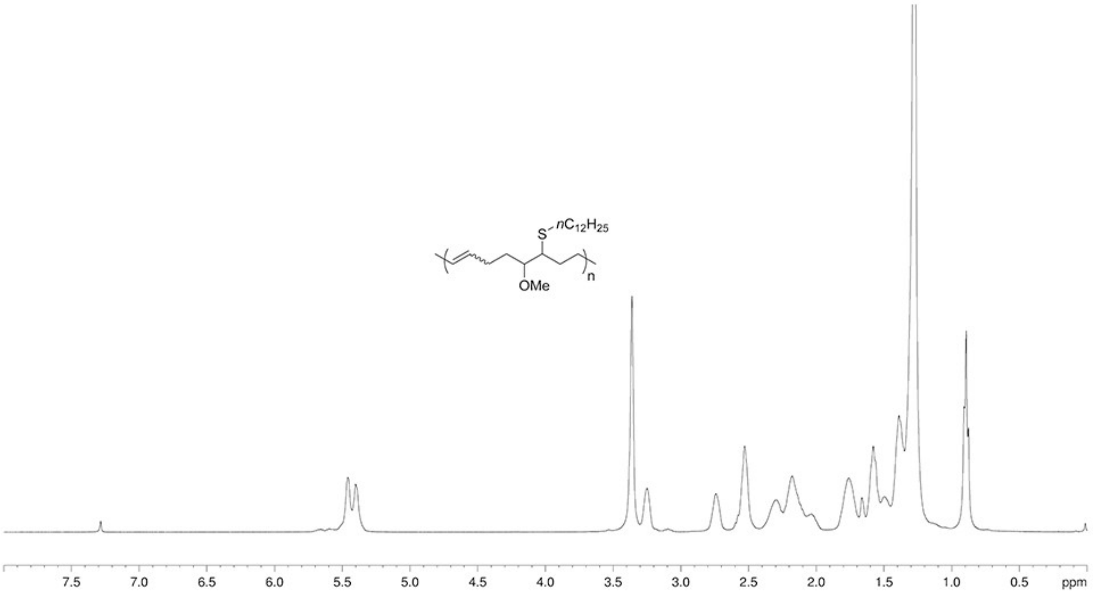

Figure S54. ^{1}H NMR (400 MHz, $CDCl_3$, 25 °C) of Polymer **6g**.

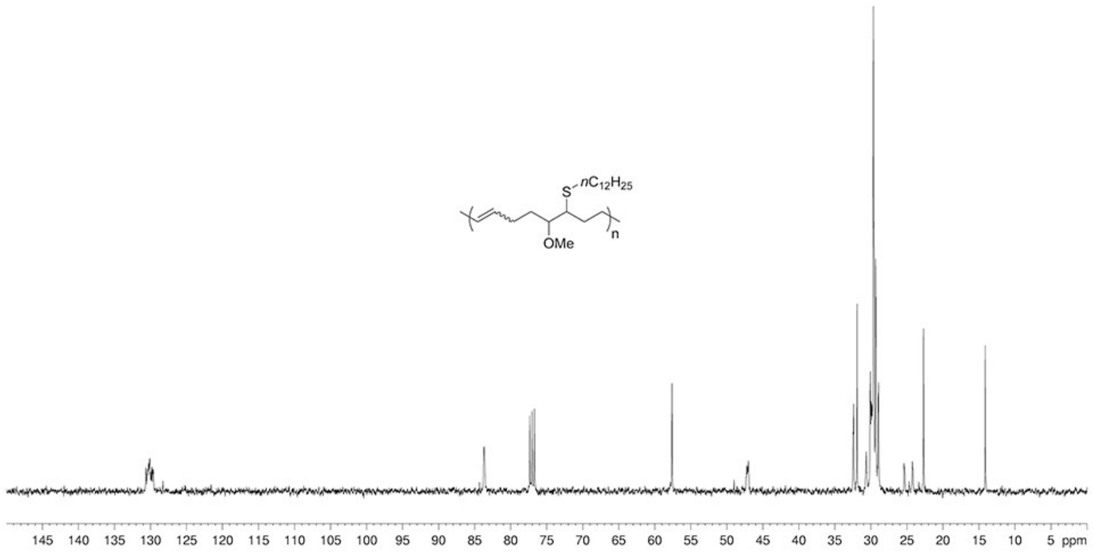

Figure S55. ^{13}C NMR (100 MHz, $CDCl_3$, 25 °C) of Polymer **6g**.

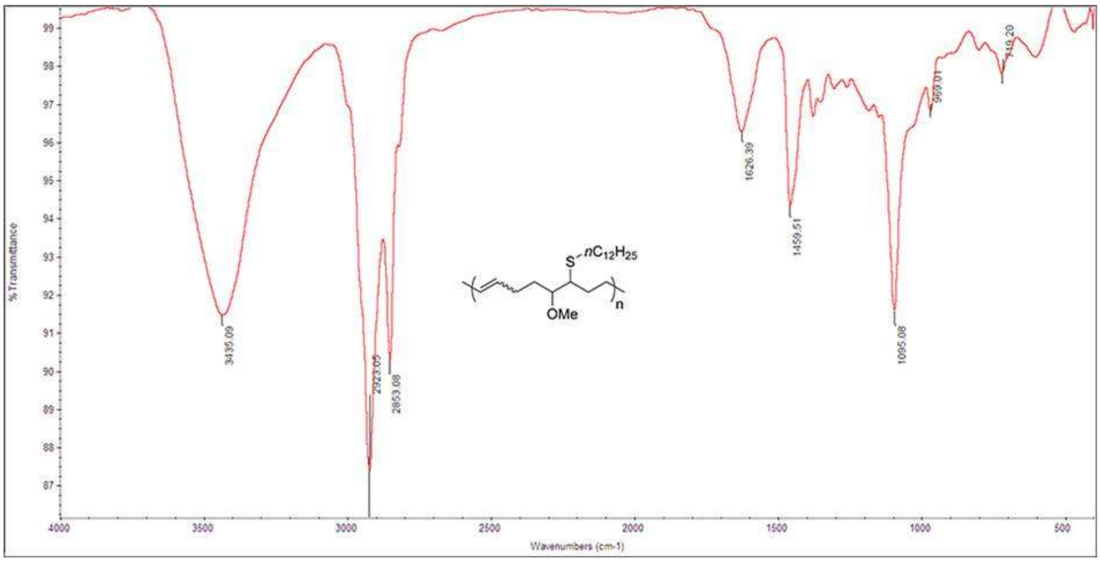

Figure S56. FT-IR spectrum of Polymer **6g**.

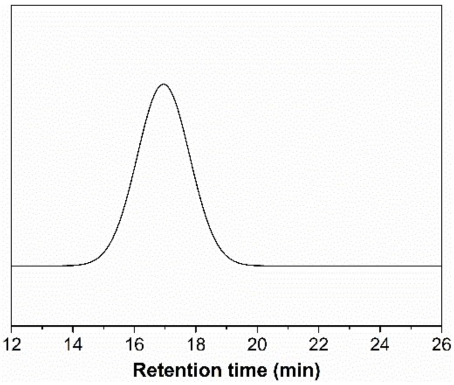

Figure S57. GPC Characterization of Polymer **6g**.

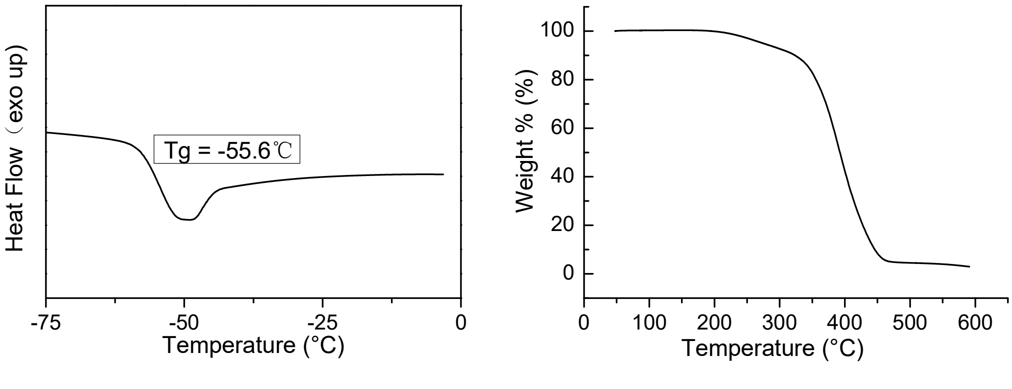

Figure S58. DSC and TGA Characterization of Polymer **6g**.

Section V: *Synthesis and Characterization* of *Monomers* ***7a****-****7h***

V-1: *The synthesis and Characterization of* ***7a***

After the solution of intermediate **4a** (1.0 mmol based on the amount of **1a**) was collected into an oven-dried vial equipped with a stir bar, the mixture was concentrated. The residue was added onto silica gel, and eluted with 0-2% EtOAc in petroleum ether. Within 30 min, **4a** was completely into **7a** during the silica gel chromatography, and the collected eluent was concentrated to afford 166.5 mg **7a** as a colorless oil (67% isolated yield).

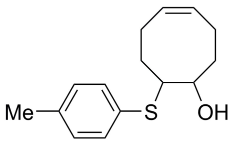

7a ^{1}H NMR (400 MHz, $CDCl_3$) δ: 7.38 (d, *J* = 8.1 Hz, 2H), 7.14 (d, *J* = 7.9 Hz, 2H), 5.75-5.67 (m, 1H), 5.58-5.51 (m, 1H), 3.77-3.66 (m, 1H), 3.41-3.24 (m, 2H), 2.60-2.45 (m, 1H), 2.46-2.38 (m, 1H), 2.36 (s, 3H), 2.32-2.23 (m, 2H), 2.20-2.12 (m, 1H), 2.11-2.01 (m, 1H), 1.88-1.79 (m, 1H), 1.78-1.70 (m, 1H) ppm; ^{13}C NMR (100 MHz, $CDCl_3$) δ: 137.8, 133.4,131.4, 129.7, 129.3, 126.9, 71.7, 55.2, 32.8, 32.1, 24.3, 24.1, 21.1 ppm; FT-IR (KBr, cm^{-1})3444, 3012, 2925, 2859, 1736, 1649, 1485, 1044,807, 733; HRMS (ESI-TOF): m/z calcd for $C_{15}H_{21}OS$ [M+H]$^+$: 249.1308; found: 249.1301.

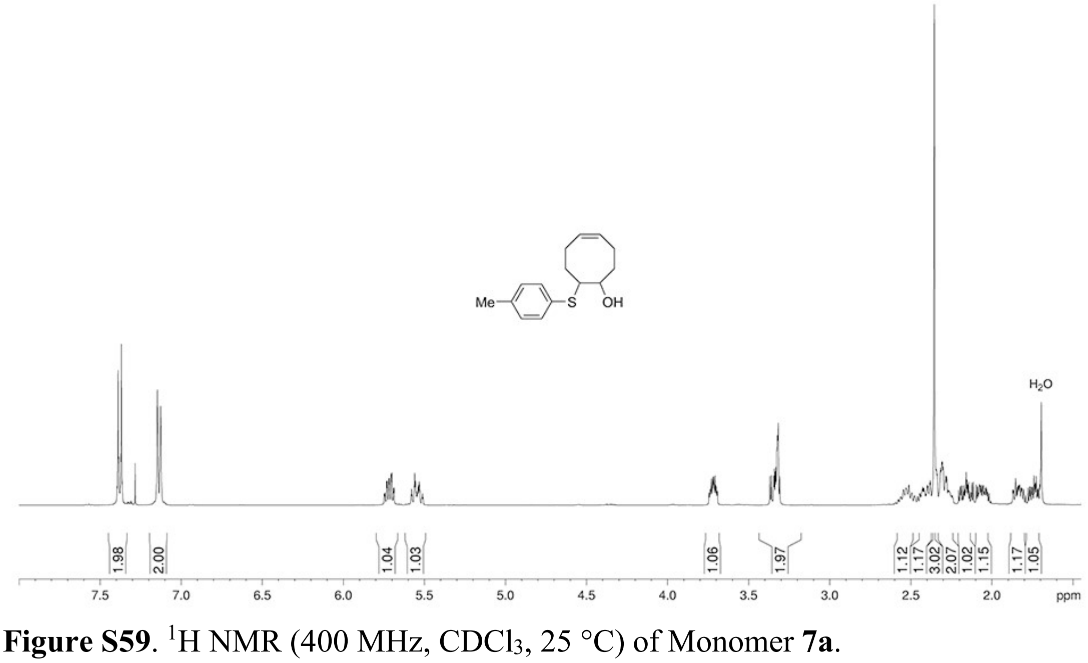

Figure S59. ^{1}H NMR (400 MHz, $CDCl_3$, 25 °C) of Monomer **7a**.

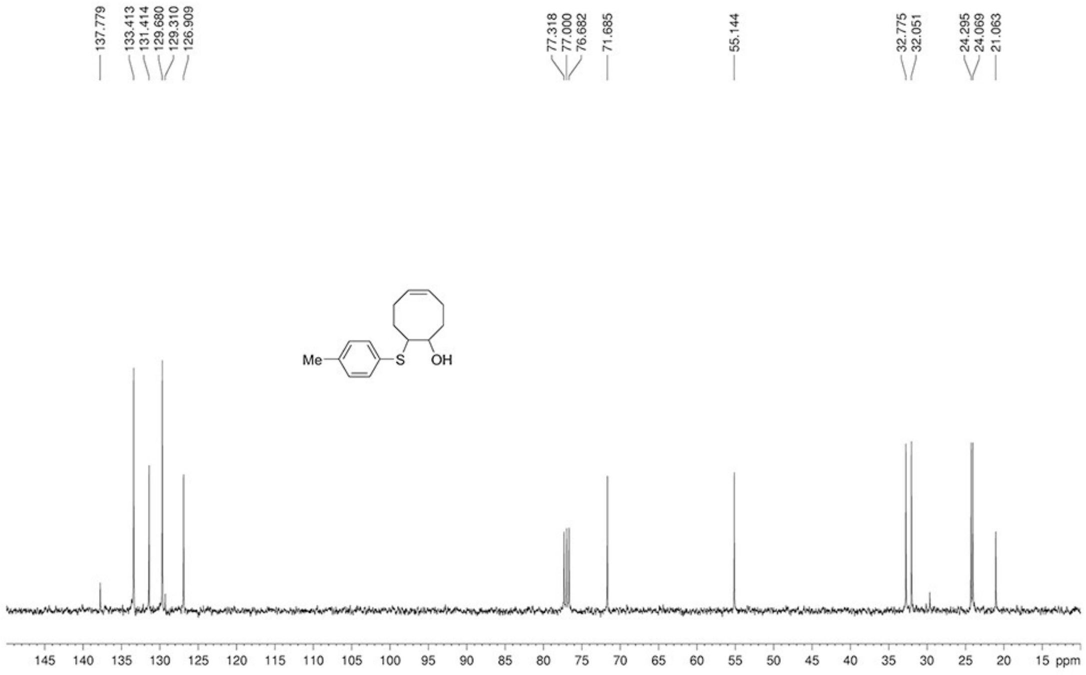

Figure S60. ^{13}C NMR (100 MHz, $CDCl_3$, 25 °C) of Monomer **7a**.

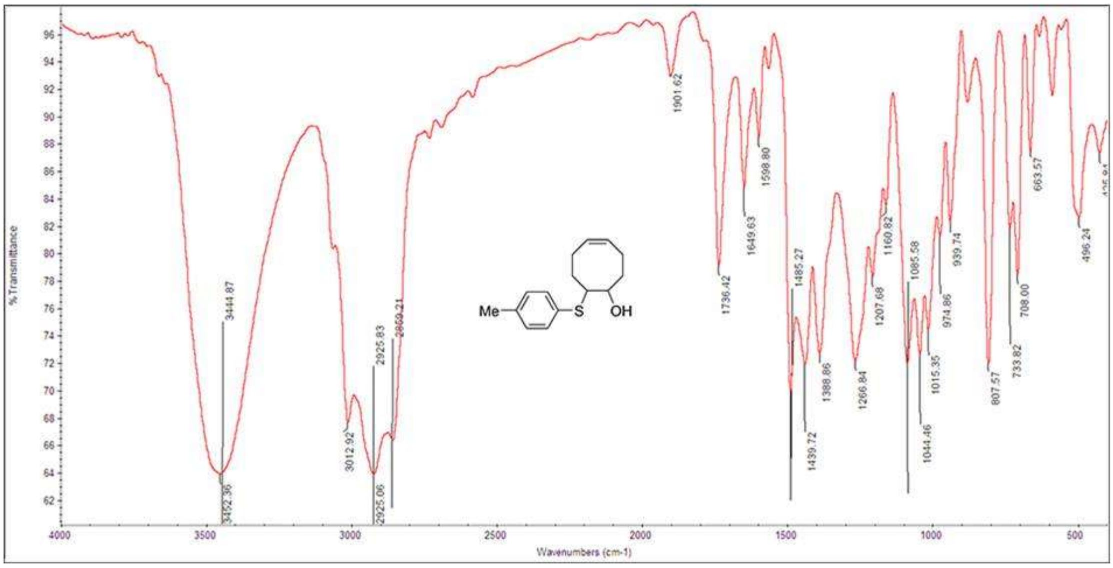

Figure S61. FT-IR spectrum of Monomer **7a**.

*V-2: The synthesis and characterization of 7**b** and 7**c***

After the solution of intermediate **4b** (1.0 mmol based on the amount of **1b**) was collected into an oven-dried vial equipped with a stir bar, the mixture was concentrated. The residue was added onto silica gel, and eluted with 0-1% EtOAc in petroleum ether. Within 30 min, **4b** was completely into 5-ArS,6-OH-COE **7a** (Ar = 4-Br-Phenyl or 4-F-Phenyl) during the silica gel chromatography, and the collected eluent was

concentrated to afford corresponding FCOE **7a** as a colorless oil. An oven-dried vial equipped with a stir bar was charged with the obtained **7a** and anhydrous THF. NaH (1.5 mmol) was added into the vial under nitrogen with an ice-water bath. After stirring at 0 ºC for 30 min, TBSCl (1.3 mmol) and imidazole (2.0 mmol) were added into the vial. Then, the mixture was stirred at room temperature for 12 h. When the reaction was completed as monitored by TLC analysis, the mixture was concentrated under vacuum. The residue was treated with DCM (150 mL) and $NaHCO_3$ (20 mL) saturated aqueous solution. The separated organic layer was washed brine for two times (2*10 mL), dried over Na_2SO_4, concentrated under vacuum. The residue was purified by column chromatography (eluting with 0-1% EtOAc in petroleum ether) to afford **7b** and **7c** in 63% and 65% isolated yield.

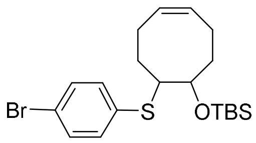

7b ^{1}H NMR (400 MHz, $CDCl_3$) δ: 7.40 – 7.36 (m, 2H), 7.26 – 7.20 (m, 2H), 5.76 – 5.69 (m, 1H), 5.64 – 5.57 (m, 1H), 4.01 – 3.96 (m, 1H), 3.60 – 3.54 (m, 1H), 2.50 – 2.32 (m, 2H), 2.20 – 2.03 (m, 4H), 1.83 – 1.67 (m, 2H), 0.93 – 0.90 (s, 9H), 0.11 (s, 3H), 0.10 (s, 3H) . ^{13}C NMR (100 MHz, $CDCl_3$) δ: 137.0, 132.0, 131.7, 130.6, 128.5, 119.6, 74.0, 53.7, 35.6, 32.1, 25.9, 24.5, 22.7, 18.3, -4.6, -4.7 ppm. FT-IR (KBr, cm^{-1}): 3016, 2950, 2929, 2855, 1471, 1385, 1252, 1090, 1007, 977, 836, 775, 730.

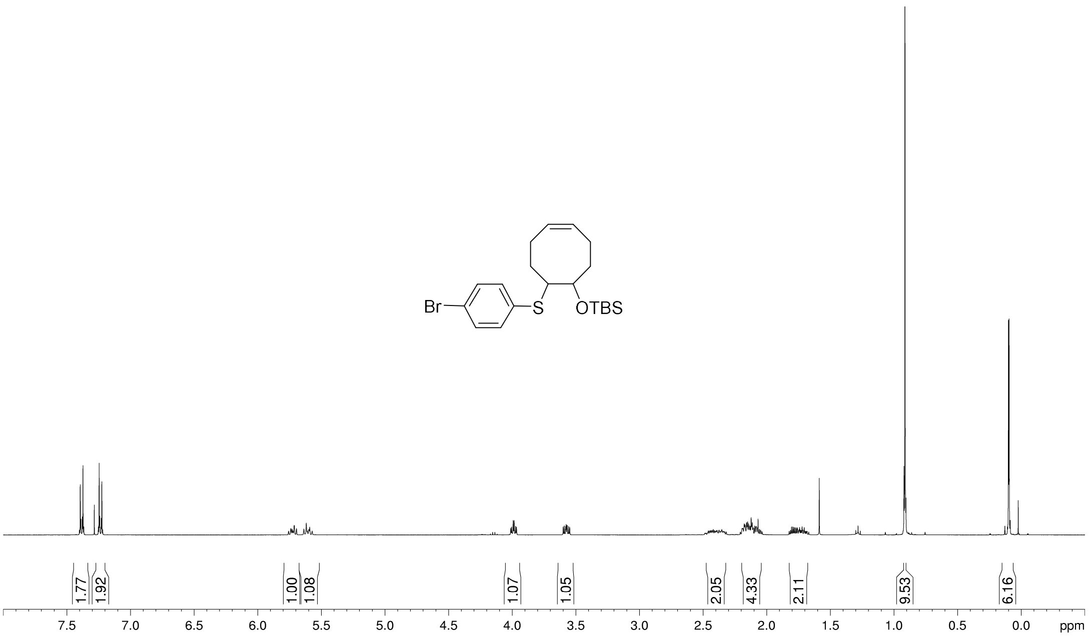

Figure S62.^{1}H NMR (400 MHz, $CDCl_3$, 25 °C) of Monomer **7b**.

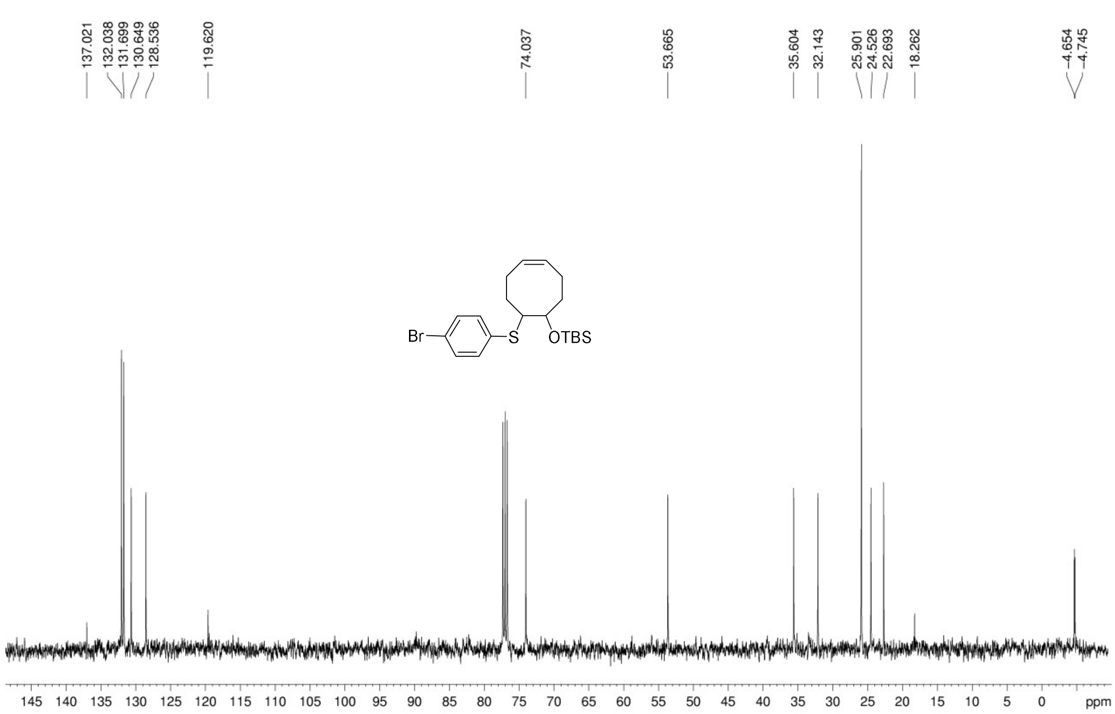

Figure S63.^{13}C NMR (100 MHz, $CDCl_3$, 25 °C) of Monomer **7b**.

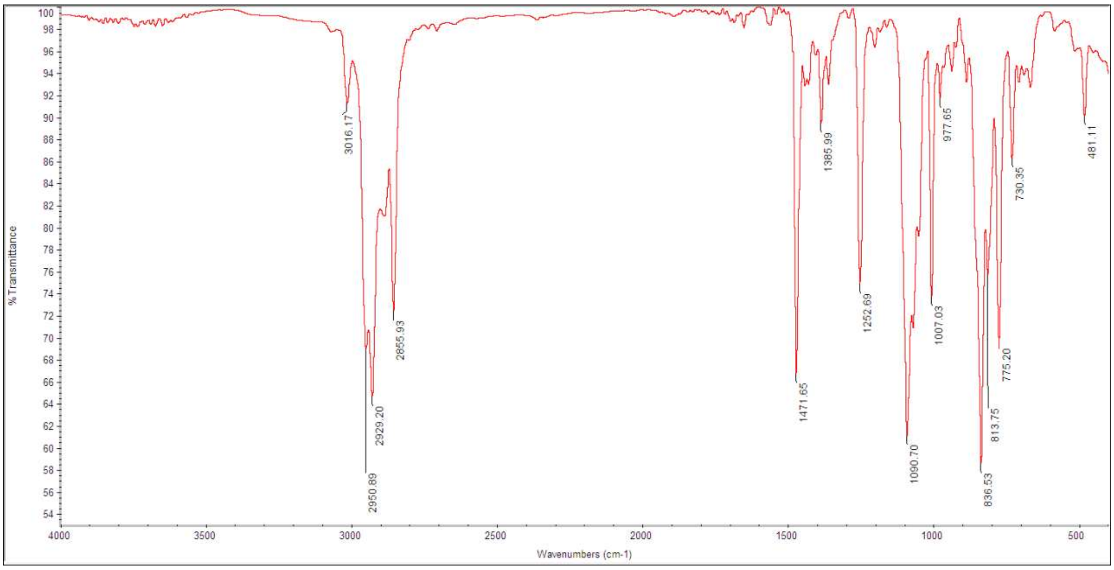

Figure S64. FT-IR spectrum of Monomer **7b**.

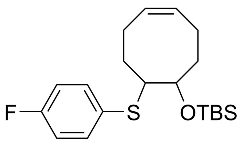

7c ^{1}H NMR (400 MHz, $CDCl_3$) δ: 7.40-7.37 (m, 2H), 7.00-7.96 (m, 2H), 5.74-5.68 (m, 1H), 5.62-5.56(m, 1H), 4.02-3.97 (m, 1H), 3.52-3.48 (m, 1H), 2.46-2.30 (m, 2H), 2.17-2.02 (m, 4H), 1.83-1.65 (m, 2H), 0.93 (s, 9H), 0.11 (s, 3H), 0.10 (s, 3H); ^{13}C NMR (100 MHz, $CDCl_3$) δ: 161.8 (d, J = 244.0 Hz), 133.6(d, J = 8.0 Hz), 132.4, 130.5, 128.7, 115.8 (d, J=22.0 Hz), 74.0, 54.8, 35.6, 32.0, 25.9, 24.5, 22.7, 18.3, -4.6, -4.8 ppm; FT-IR (KBr, cm^{-1}) 2930, 2857, 1589, 1489, 1467, 1087, 834, 774; HRMS (ESI-TOF): m/z calcd for $C_{20}H_{32}FOSSi$ $[M+H]^+$: 367.1922; found: 367.1922.

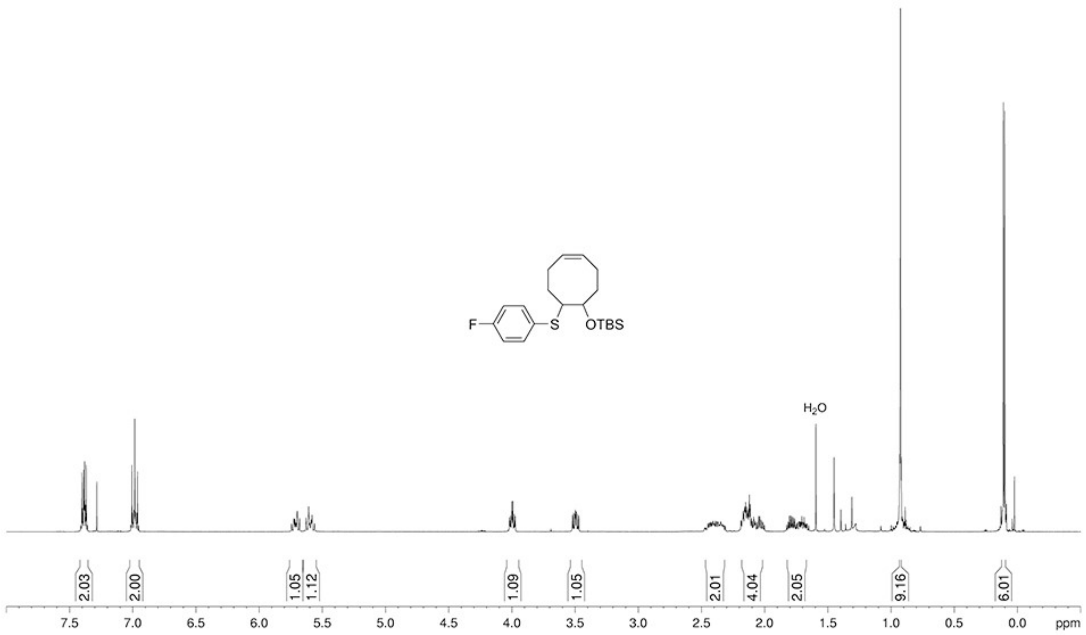

Figure S65. ^{1}H NMR (400 MHz, $CDCl_3$, 25 °C) of Monomer **7c**.

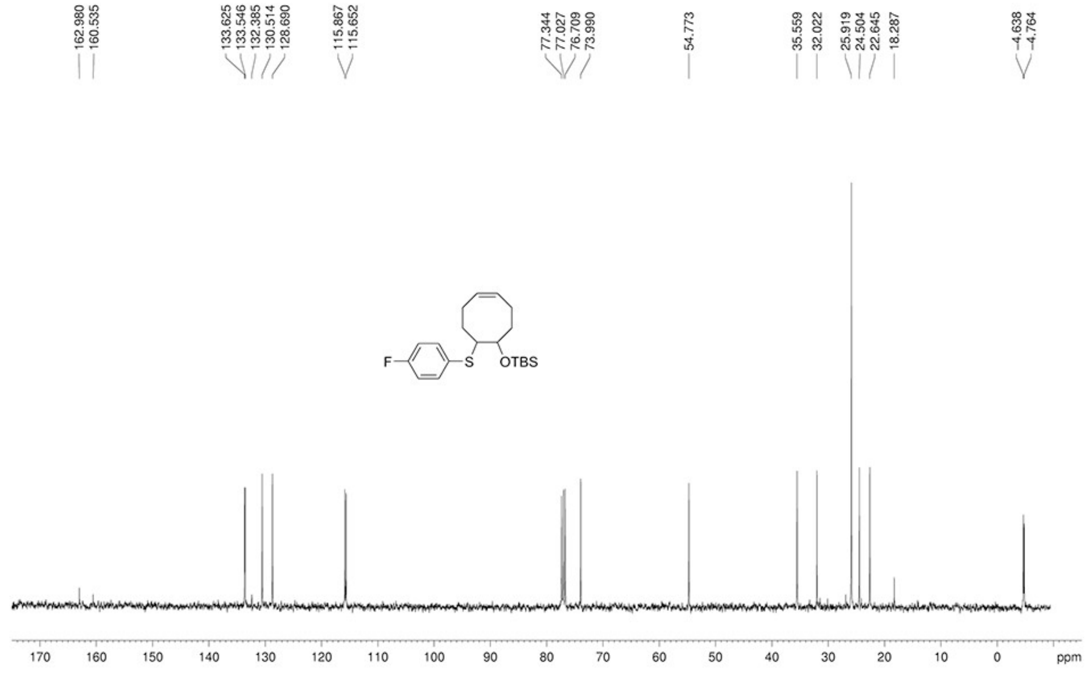

Figure S66. ^{13}C NMR (100 MHz, $CDCl_3$, 25 °C) of Monomer **7c**.

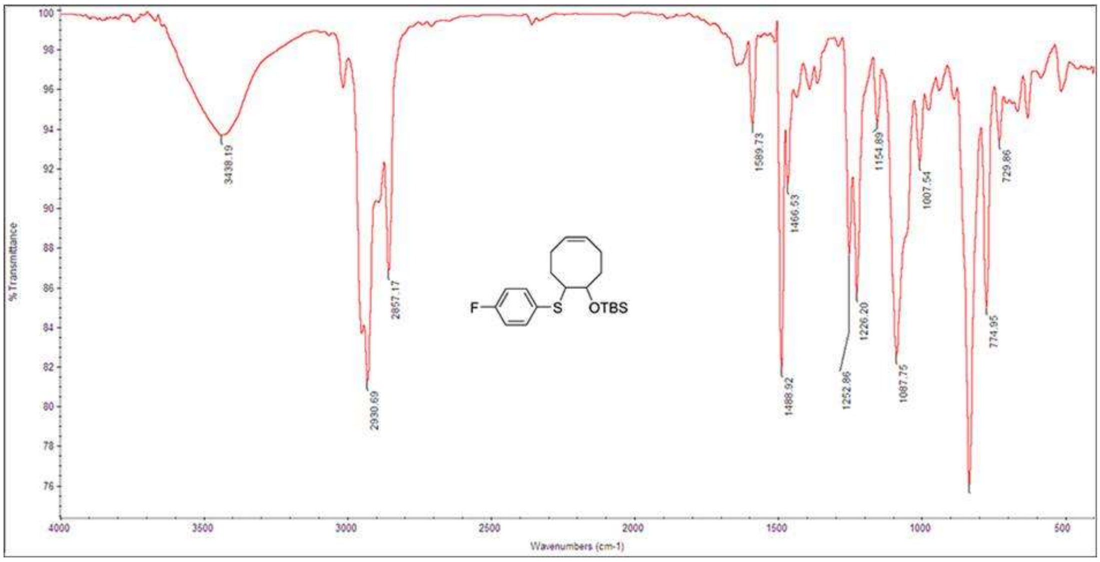

Figure S67. FT-IR spectrum of Monomer **7c**.

*V-3: The synthesis and characterization of 7**d***

An oven-dried vial equipped with a stir bar was charged with the crude product of **7h** (the amount of **7h** is based on 1.0 mmol **1b**) and anhydrous THF. NaH (1.5 mmol) was added into the vial under nitrogen with an ice-water bath. After stirring at 0 ºC for 30 min, BnBr (1.2 mmol) was added into the vial. Then, the mixture was stirred at room temperature for 12 h. When the reaction was completed as monitored by TLC analysis, the mixture was concentrated under vacuum. The residue was treated with DCM (150 mL) and NH_4Cl (20 mL) saturated aqueous solution. The separated organic layer was washed brine for two times (2*10 mL), dried over Na_2SO_4, concentrated under vacuum. The residue was purified by column chromatography (eluting with 0-1% EtOAc in petroleum ether) to afford 210.8 mg **7d** in 62% isolated yield.

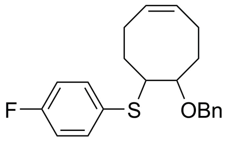

7d ^{1}H NMR (400 MHz, $CDCl_3$) δ: 7.43-7.28 (m, 7H), 7.00-6.94 (m, 2H), 5.74-5.65 (m, 1H), 5.62-5.54 (m, 1H), 4.61 (d, *J*=1.7 Hz, 2H), 3.71-3.67 (m, 1H), 3.63-3.57 (m, 1H), 2.51-2.41 (m, 2H), 2.25-2.17 (m, 2H), 2.14-2.06 (m, 2H), 1.93-1.86 (m, 1H), 1.75-1.69 (m, 1H) ppm; ^{13}C NMR (100 MHz, $CDCl_3$) δ:162.0 (d, *J* = 245.1 Hz), 138.5, 134.4 (d, *J* = 8.0 Hz), 131.5, 130.3, 128.3, 128.3, 127.8, 127.5, 115.8 (d, *J* = 21.0 Hz), 81.3, 72.3,

53.6, 32.0, 31.1, 24.6, 23.3 ppm; FT-IR (KBr, cm^{-1}) 3020, 2929, 2850, 1588, 1488, 1456, 1224, 1090, 828, 735; HRMS (ESI-TOF): m/z calcd for $C_{21}H_{24}FOS$ $[M+H]^+$: 343.1526; found: 343.1525.

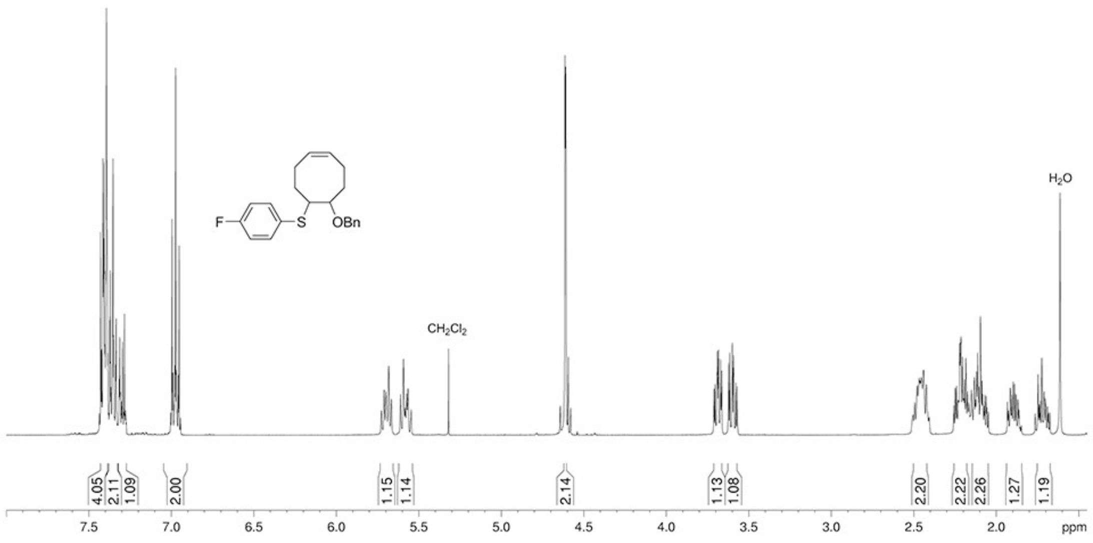

Figure S68. ^{1}H NMR (400 MHz, $CDCl_3$, 25 °C) of Monomer **7d**.

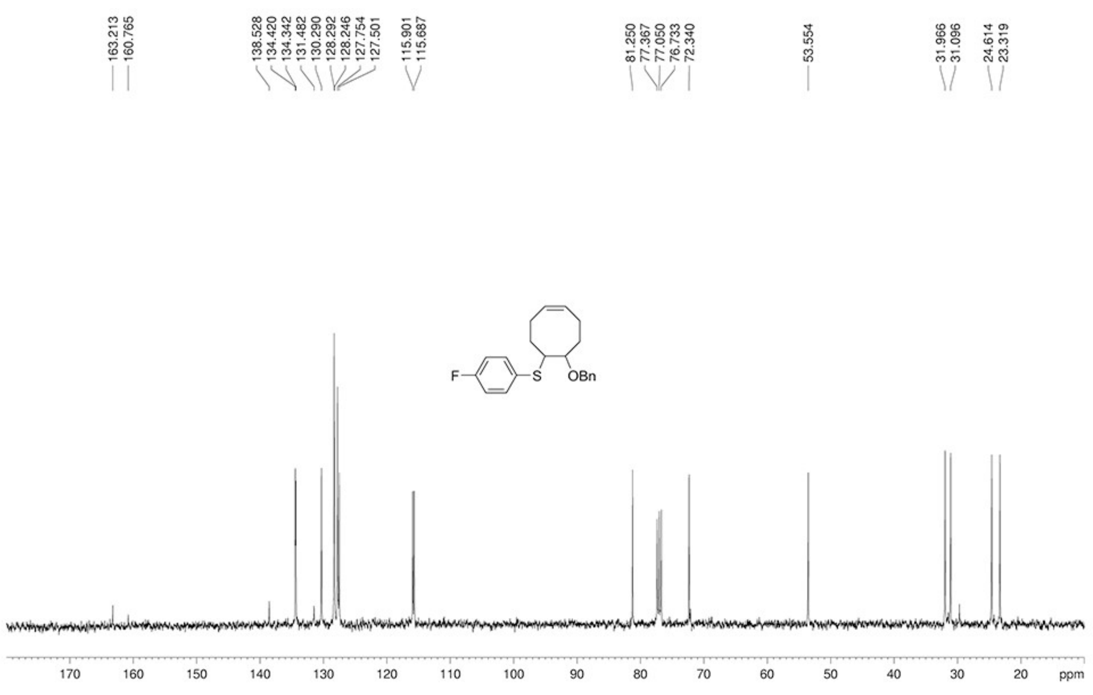

Figure S69. ^{13}C NMR (100 MHz, $CDCl_3$, 25 °C) of Monomer **7d**.

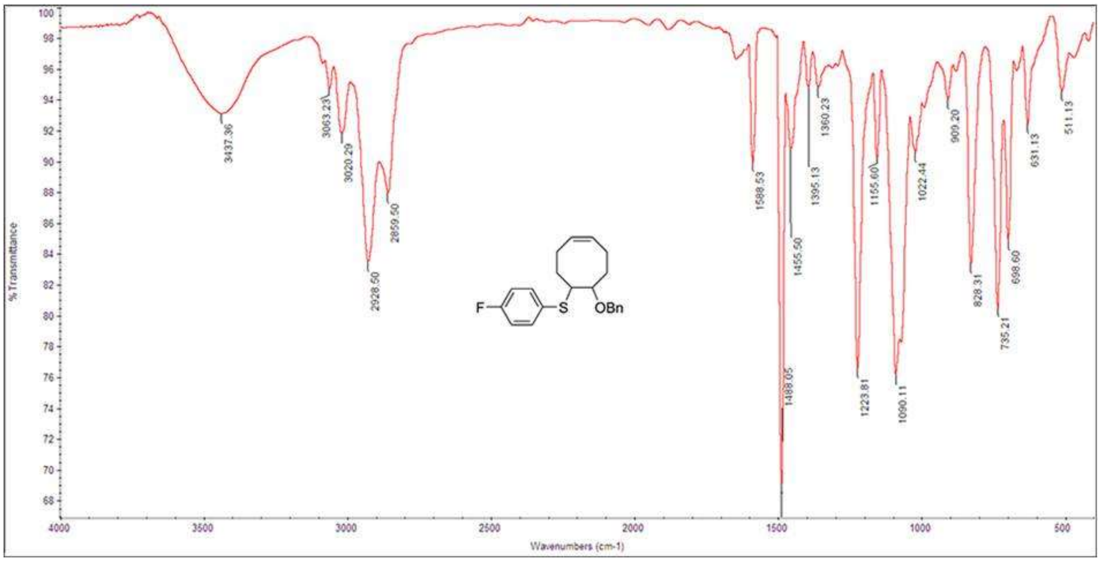

Figure S70. FT-IR spectrum of Monomer **7d**.

*V-4: The synthesis and characterization of 7**e***

An oven-dried vial equipped with a stir bar was charged with the crude product of **7h** (the amount of **7h** is based on 1.0 mmol **1b**) and 1.5 mL anhydrous DCM. Dicyclohexylcarbodiimid (DCC, 2.0 mmol), acetic acid (2.0 mmol) and DMAP (0.05 mmol) was added into the vial at 0 ºC. The mixture was stirred at room temperature for 12 h. When the reaction was completed as monitored by TLC analysis, the mixture was concentrated under vacuum. The residue was treated with DCM (150 mL) and NH_4Cl (20 mL) saturated aqueous solution. The separated organic layer was washed brine for two times (2*10 mL), dried over Na_2SO_4, concentrated under vacuum. The residue was purified by column chromatography (eluting with 0-2% EtOAc in petroleum ether) to afford 158.6 mg **7e** (54% isolated yield).

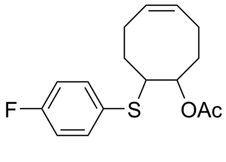

7e ^{1}H NMR (400 MHz, $CDCl_3$) δ: 7.44-7.37 (m, 2H), 7.04-6.97 (m, 2H), 5.78-5.66 (m, 1H), 5.66-5.55 (m, 1H), 5.16-5.11 (m, 1H), 3.56-3.51 (m, 1H), 2.54-248 (m, 1H), 2.43-2.32 (m, 1H), 2.24-2.04 (m, 4H), 2.01 (s, 3H), 1.84-1.75 (m, 2H) ppm; ^{13}C NMR (100 MHz, $CDCl_3$) δ: 170.2, 162.1 (d, J = 245.0 Hz), 134.0(d, J = 8.0 Hz), 130.8, 130.2, 128.0, 115.9 (d, J = 22.0 Hz), 75.5, 51.2, 32.2, 31. 7, 24.5, 23.6, 21.1 ppm; FT-IR (KBr,

cm^{-1}) 3016, 2935, 1735, 1642, 1589, 1448, 1229, 1087, 1031, 830, 733; HRMS (ESI-TOF): m/z calcd for $C_{16}H_{20}FO_2S$ $[M+H]^+$: 295.1163; found: 295.1162.

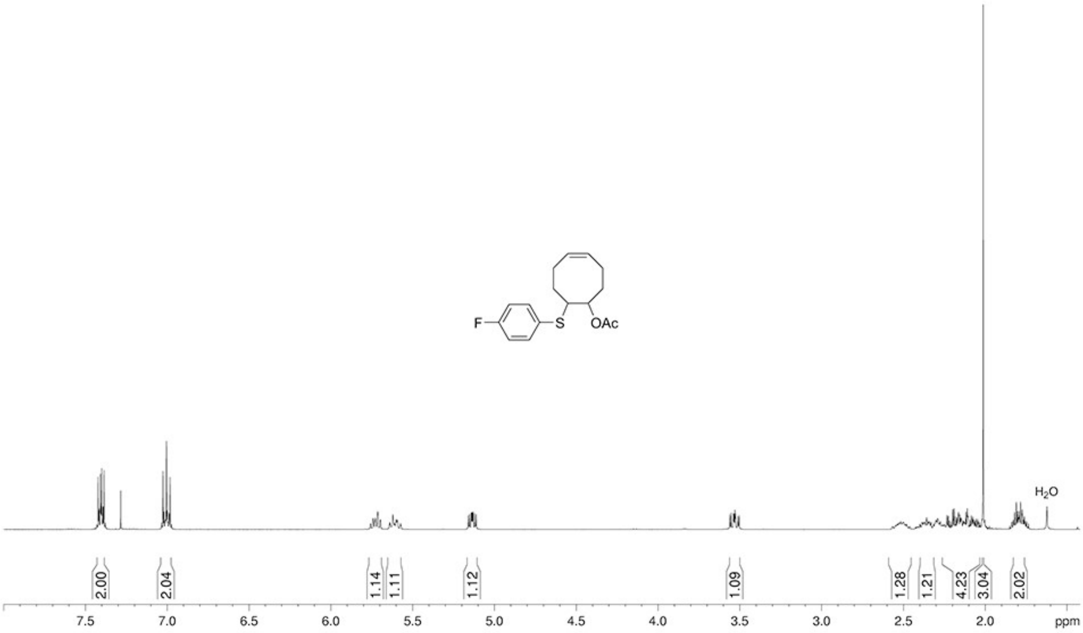

Figure S71. ^{1}H NMR (400 MHz, $CDCl_3$, 25 °C) of Monomer **7e**.

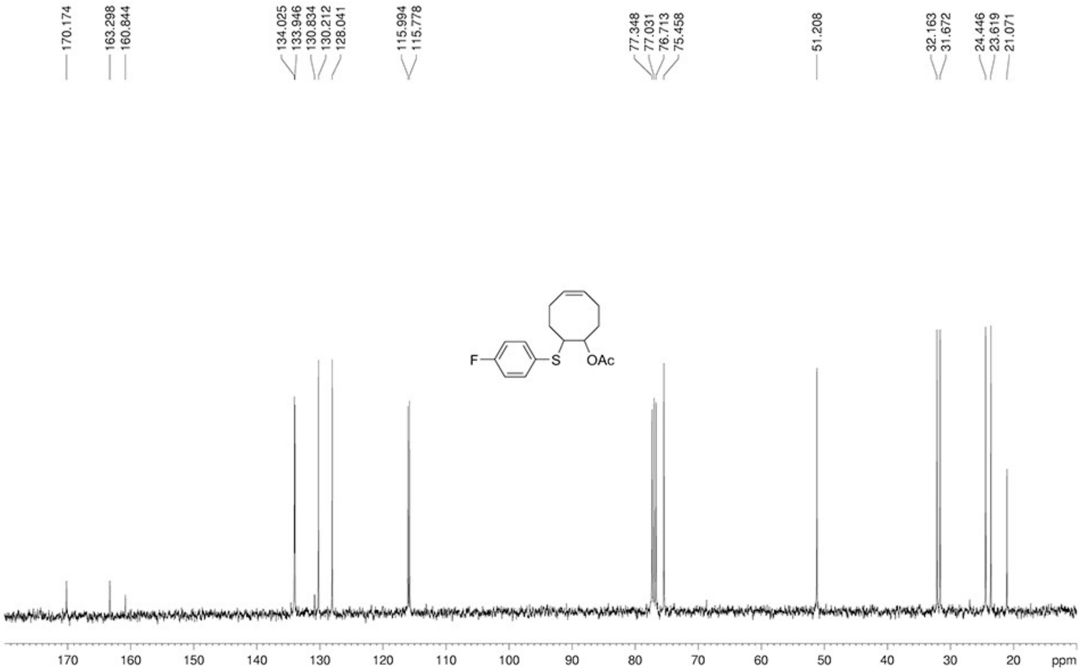

Figure S72. ^{13}C NMR (100 MHz, $CDCl_3$, 25 °C) of Monomer **7e**.

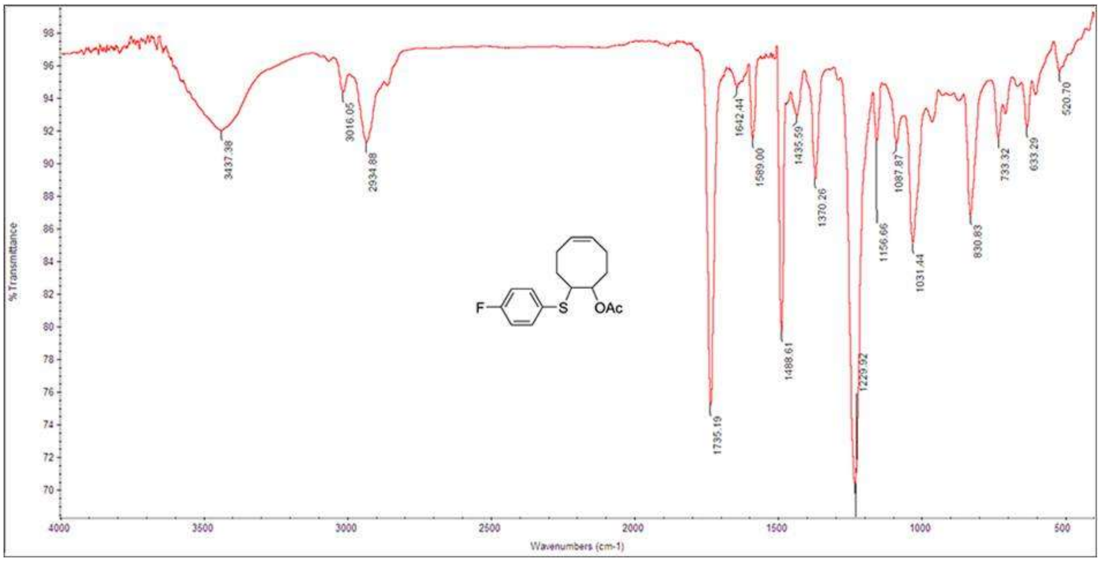

Figure S73. FT-IR spectrum of Monomer **7e**.

V-5: *The synthesis and characterization of 7**f***

After the solution of intermediate **4a** (0.5 mmol based on the amount of **1a**) was collected into an oven-dried vial equipped with a stir bar, morpholine (2.0 mmol) was added via a syringe at room temperature. When the substitution reaction was completed as monitored by TLC analysis, the mixture was treated with DCM (100 mL) and $NaHCO_3$ (15 mL) saturated aqueous solution. The separated organic layer was washed brine for two times (2*10 mL), dried over Na_2SO_4, concentrated under vacuum. The residue was purified by column chromatography (eluting with 0-1% EtOAc in petroleum ether) to afford 93.1 mg **7e** (59% isolated yield) as a solid. The crystal of **7f** is obtained via recrystallization from a mixed solvent of DCM and *n*Hexane (for the X-ray characterization result of **7f**, see Section VIII).

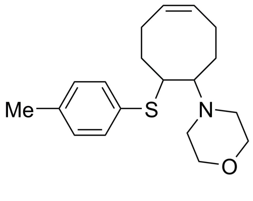

7f ^{1}H NMR (400 MHz, $CDCl_3$) δ: 7.32-7.25 (m, 2H), 7.10 (d, J = 7.9 Hz, 2H), 5.67-5.55 (m, 2H), 3.77-3.70 (m, 4H), 3.49-3.44 (m, 1H), 2.94-2.86 (m, 2H), 2.80-2.75(m, 1H), 2.54-2.46 (m, 2H), 2.45-2.26 (m, 5H), 2.23-2.16 (m, 1H), 2.08-1.89 (m, 3H), 1.68-1.54 (m, 2H) ppm; ^{13}C NMR (100 MHz, $CDCl_3$) δ: 136.2, 134.0, 132.3, 130.1, 129.5,

128.6, 67.0, 65.6, 53.3, 50.5, 33.3, 25.9, 25.1, 24.1, 21.1ppm; FT-IR (KBr, cm^{-1}) 2928, 2855, 1640, 1490, 1446, 1113, 807, 731; HRMS (ESI-TOF): m/z calcdfor $C_{19}H_{28}NOS$ $[M+H]^+$: 318.1886; found: 318.1880.

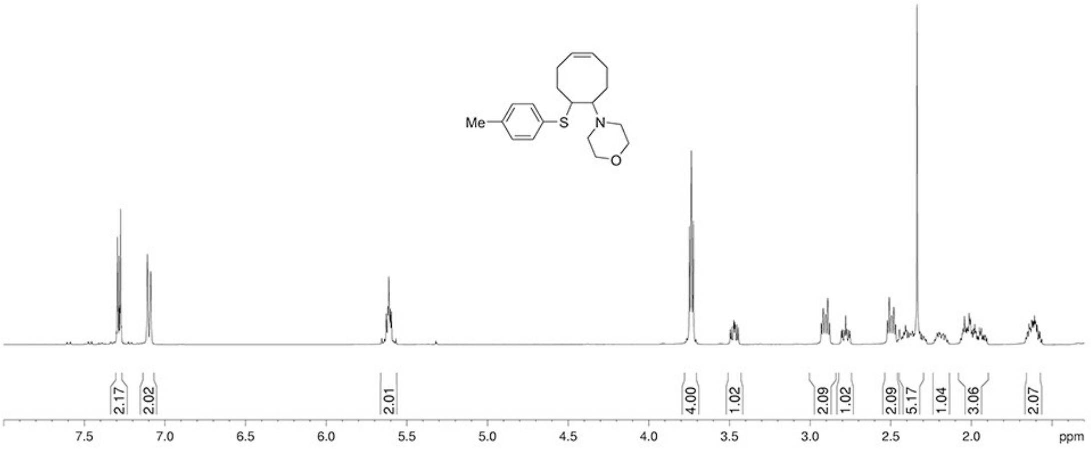

Figure S74. ^{1}H NMR (400 MHz, $CDCl_3$, 25 °C) of Monomer **7f**.

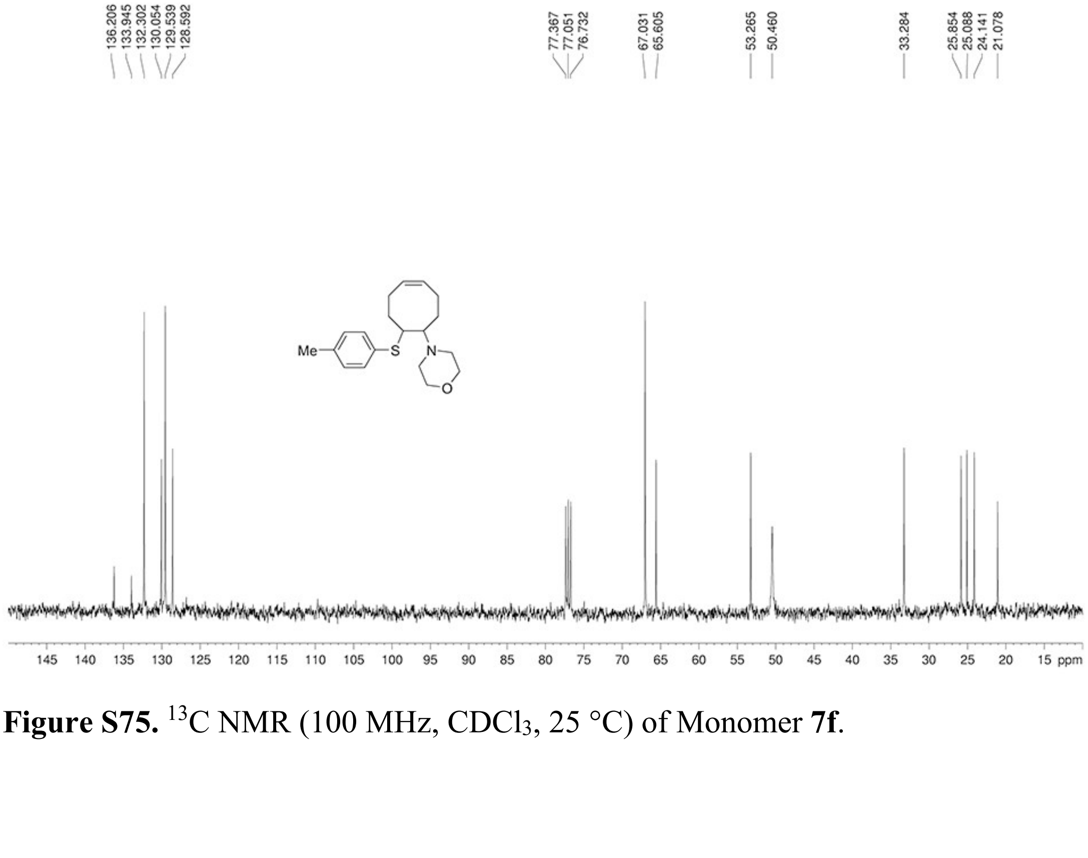

Figure S75. ^{13}C NMR (100 MHz, $CDCl_3$, 25 °C) of Monomer **7f**.

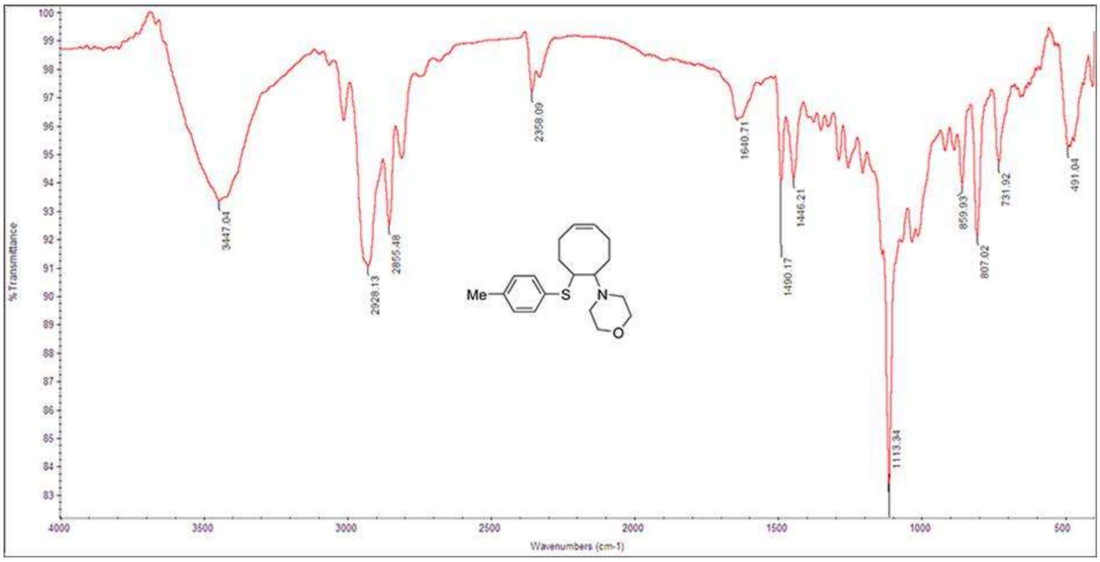

Figure S76. FT-IR spectrum of Monomer **7f**.

V-6: The synthesis and characterization of 7g

After the solution of intermediate **4a** (0.5 mmol based on the amount of **1a**) was collected into an oven-dried vial equipped with a stir bar, N(*n*Bu)$_4$N$_3$ (2.0 mmol) was added at room temperature. When the substitution reaction was completed as monitored by TLC analysis, the mixture was treated with DCM (100 mL) and $NaHCO_3$ (15 mL) saturated aqueous solution. The separated organic layer was washed brine for two times (2*10 mL), dried over Na_2SO_4, concentrated under vacuum. The residue was purified by column chromatography (eluting with 0-2% EtOAc in petroleum ether) to afford 75.1 mg **7f** (55% isolated yield).

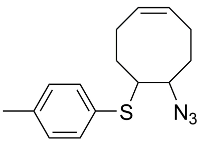

7g ^{1}H NMR (400 MHz, $CDCl_3$) δ: 7.41-7.33 (m, 2H), 7.18-7.09 (m, 2H), 5.75-5.54 (m, 2H), 3.75-3.70 (m, 1H), 3.48-3.42 (m, 1H), 2.53-2.45 (m, 2H), 2.36 (s, 3H), 2.32-2.11 (m, 4H), 1.95-1.88 (m, 1H), 1.83-1.75 (m, 1H) ppm; ^{13}C NMR (100 MHz, $CDCl_3$) δ: 137.4, 133.0, 131.2, 129.8, 129.1, 129.0, 65.0, 52.7, 32.0, 31.8, 24.7, 23.7, 21.1 ppm; FT-IR (KBr, cm^{-1}) 3017, 2926, 2098, 1650, 1490, 1439, 1259, 1094, 808, 734; HRMS (ESI-TOF): m/z calcd for $C_{15}H_{20}N_3S$ [M+H]$^+$: 274.1372; found: 274.1366.

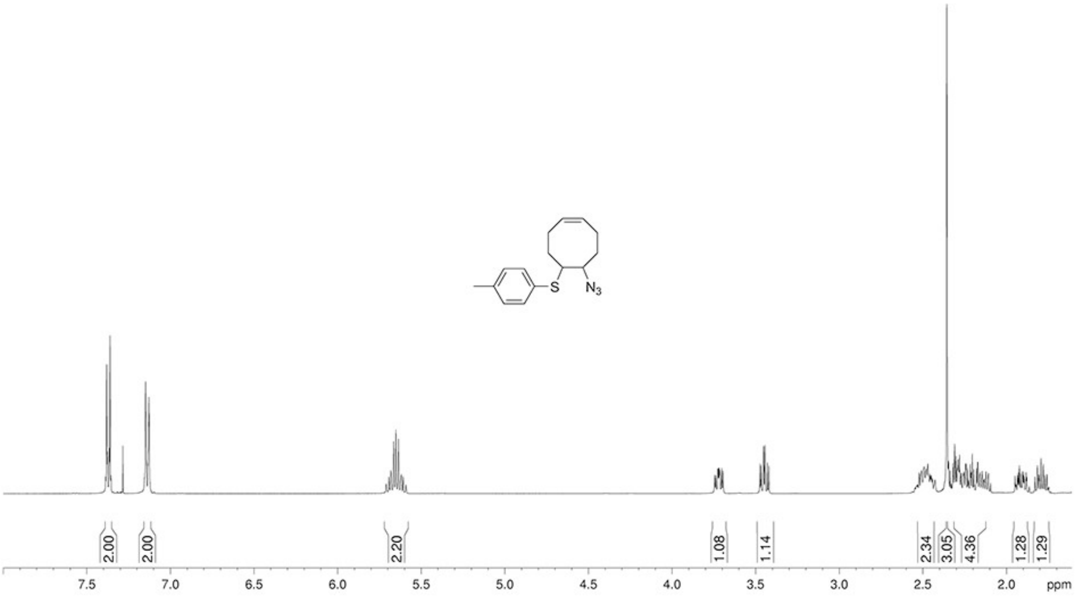

Figure S77. ^{1}H NMR (400 MHz, $CDCl_3$, 25 °C) of Monomer **7g**.

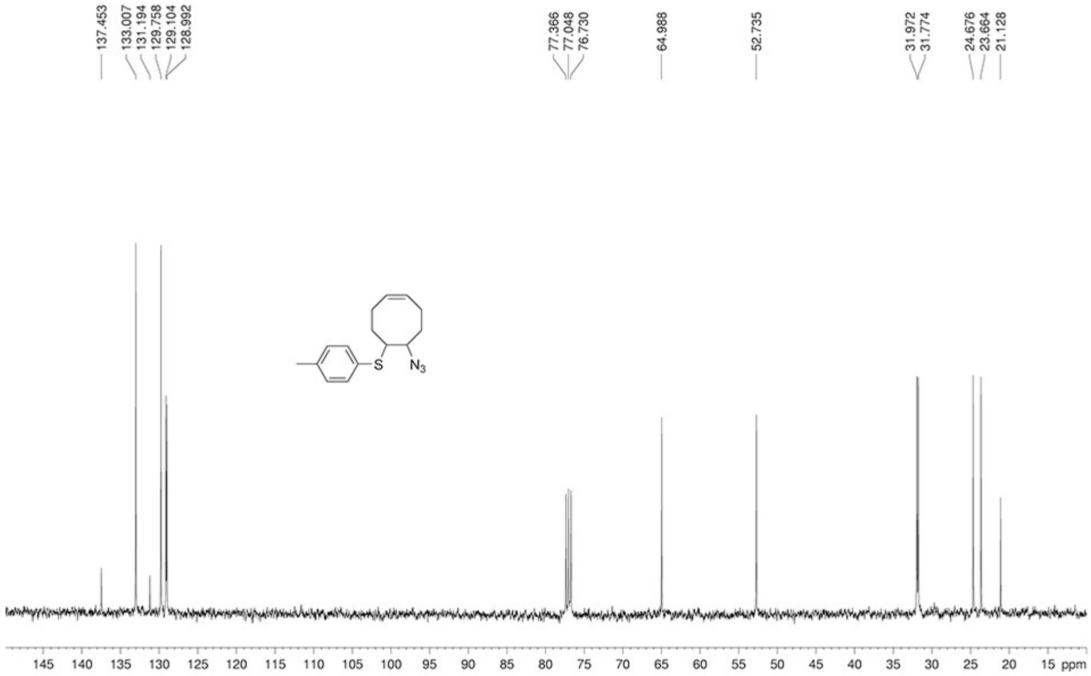

Figure S78. ^{13}C NMR (100 MHz, $CDCl_3$, 25 °C) of Monomer **7g**.

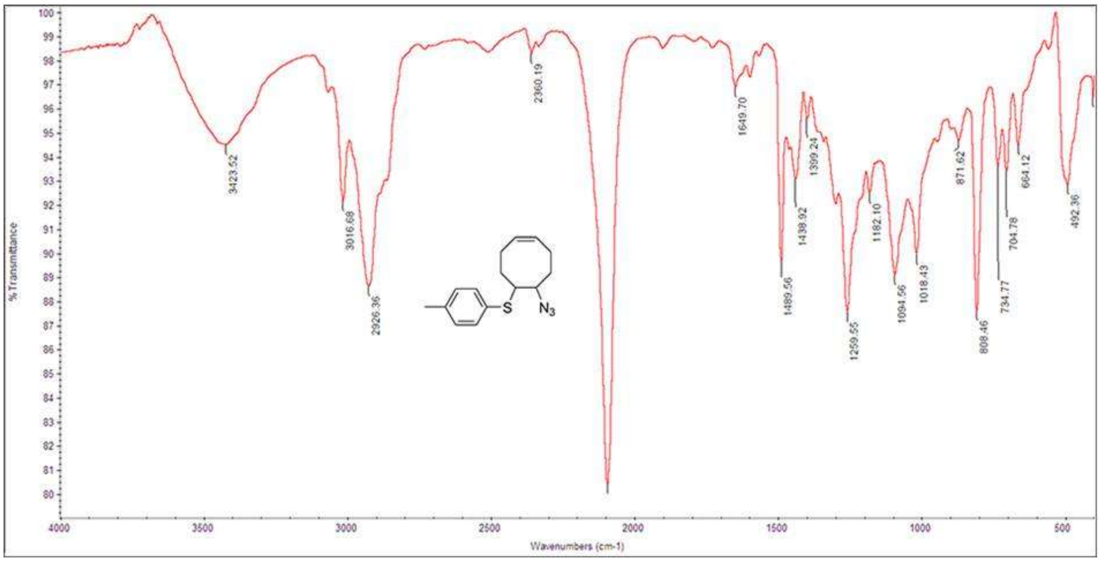

Figure S79. FT-IR spectrum of Monomer **7g**.

*V-7: The synthesis and characterization of 7**h***

After the solution of intermediate **4a** (0.5 mmol based on the amount of **1a**) was collected into an oven-dried vial equipped with a stir bar, imidazole (3.0 mmol) was added. The mixture was stirred at 60 °C overnight. When the substitution reaction was completed as monitored by TLC analysis, the mixture was treated with DCM (100 mL) and $NaHCO_3$ (15 mL) saturated aqueous solution. The separated organic layer was washed brine for two times (2*10 mL), dried over Na_2SO_4, concentrated under vacuum. The residue was purified by column chromatography (eluting with 0-20% EtOAc in petroleum ether) to afford 66.8 mg **7h** (45% isolated yield).

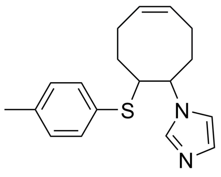

7h ^{1}H NMR (400 MHz, $CDCl_3$) δ: 7.54 (s, 1H), 7.12-7.10 (m, 2H), 7.07-7.03 (m, 3H), 6.94 (t, J = 1.2 Hz, 1H), 5.79-5.73 (m, 1H), 5.70-5.64 (m, 1H), 4.51-4.32 (m, 1H), 3.65-3.59 (m, 1H), 2.69-2.59 (m 2H), 2.51-2.42 (m, 1H), 2.32 (s, 3H), 2.29-2.22 (m, 2H), 2.20-2.14 (m, 2H), 1.90-1.82 (m, 1H) ppm; ^{13}C NMR (100 MHz, $CDCl_3$) δ: 137.7, 136. 9, 133.3, 130.7, 130.5, 129.7, 128.9, 127.2, 118.0, 60.0, 55.4, 34.3, 31.2, 25.4, 24.0,

21.1ppm; FT-IR (KBr, cm^{-1}) 3014, 2923, 1645, 1498, 1226, 1081, 808, 732, 663; HRMS (ESI-TOF): m/z calcd for $C_{18}H_{23}N_2S$ $[M+H]^+$: 299.1576; found: 299.1568.

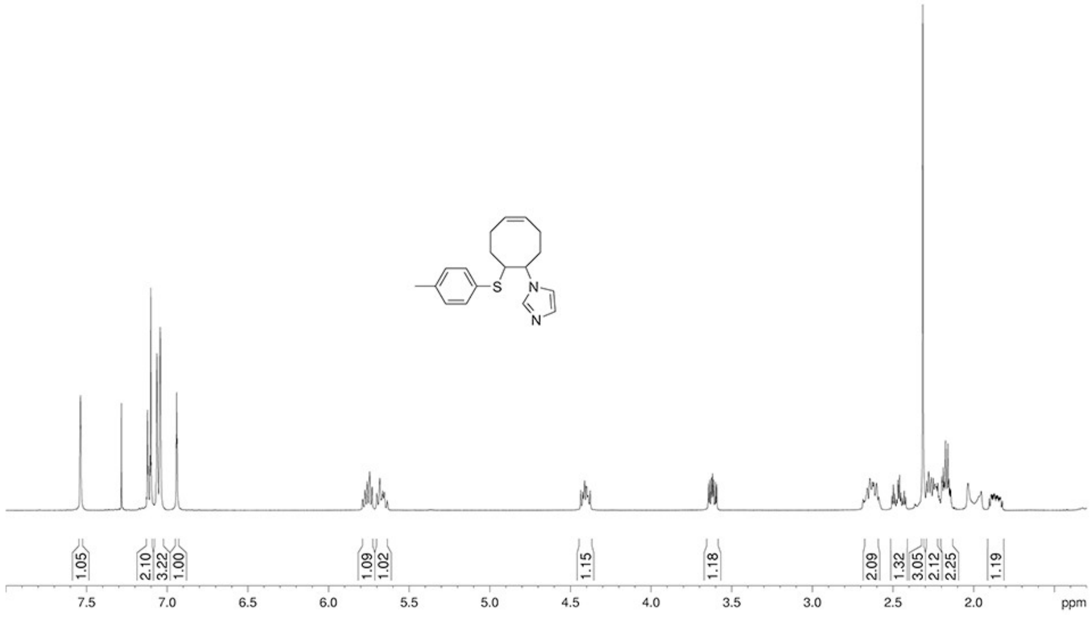

Figure S80. ^{1}H NMR (400 MHz, $CDCl_3$, 25 °C) of Monomer **7h**.

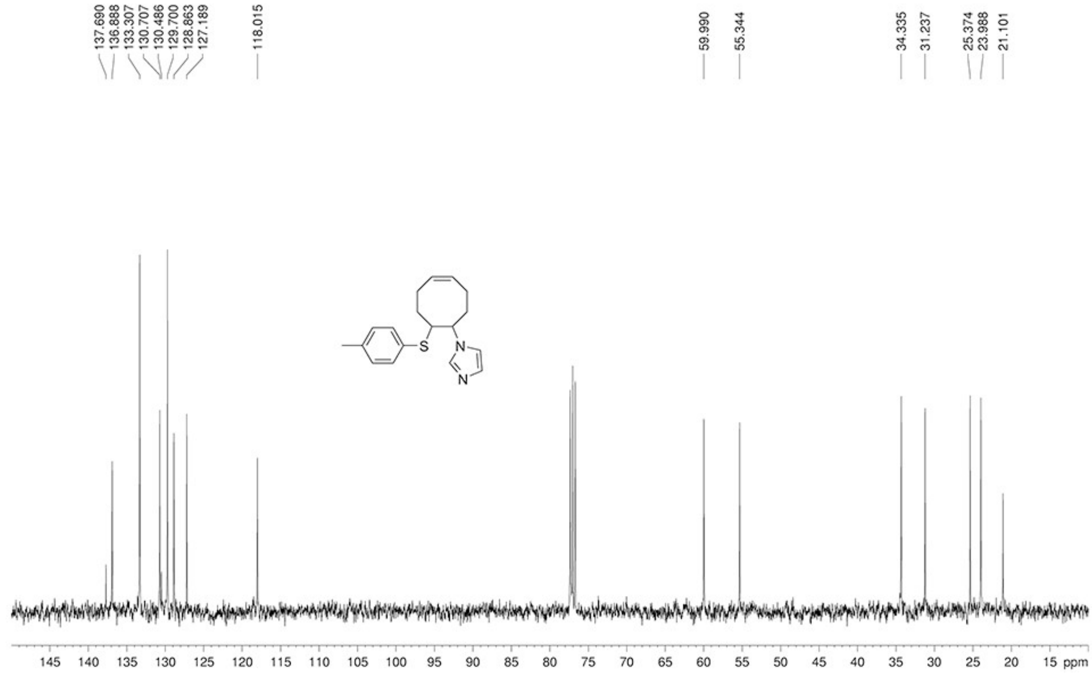

Figure S81. ^{13}C NMR (100 MHz, $CDCl_3$, 25 °C) of Monomer **7h**.

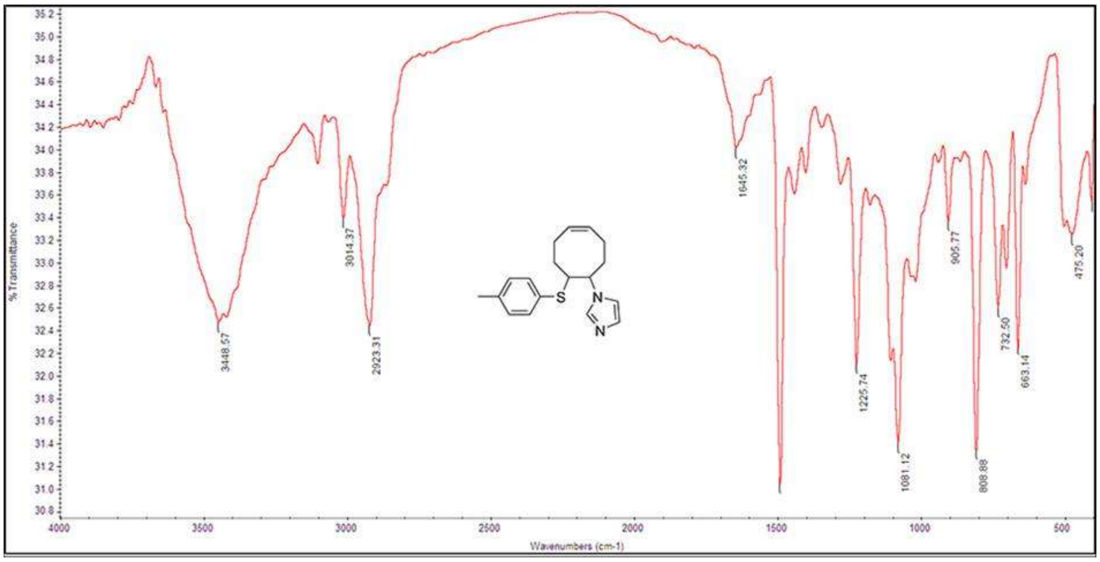

Figure S82. FT-IR spectrum of Monomer **7h**.

Section VI *Synthesis and Characterization* of *Polymer*s **8a-8f**

VI-1: *General procedure for the synthesis of* ***8a-8g***

An oven-dried vial equipped a stir bar was charged with 1.0 mL solution of monomer **7** (0.5 M) in anhydrous DCM under N_2. The G2 (Grubbs second-generation) catalyst solution (100μL, 8.5 mg/mL in degassed DCM) was added via a micro syringe into the vial at room temperature. After stirring for 24-48 h, the mixture was concentrated and was dropwisely added into MeOH with vigorously stirring. Solid compound was collected and re-dissolved in minimal amount of DCM. The precipitation procedure was repeated for three times in total. The combined organic layer was concentrated. The resulted residue was purified by column chromatography to recovery the unreacted monomer and calculated the monomer conversion. The produced polymer was characterized with ^{1}H NMR, ^{13}C NMR, FT-IR, GPC, DSC and TGA analysis.

VI-2: *Characterization of* ***8a-8g***

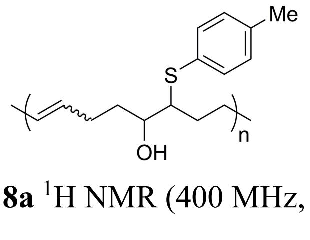

8a ^{1}H NMR (400 MHz, $CDCl_3$) δ:7.41-7.24 (m, 2H), 7.20-7.01 (m, 2H), 5.50-5.27 (m, 2H), 3.67-3.46 (m,1H), 3.02-2.86 (m, 1H), 2.75-2.47 (m, 1H), 2.45-1.94 (m, 6H), 1.86-

1.39 (m, 5H) ppm; FT-IR (KBr, cm^{-1}) 3436, 3008, 2917, 2856, 1650, 1491, 1088, 807, 705.

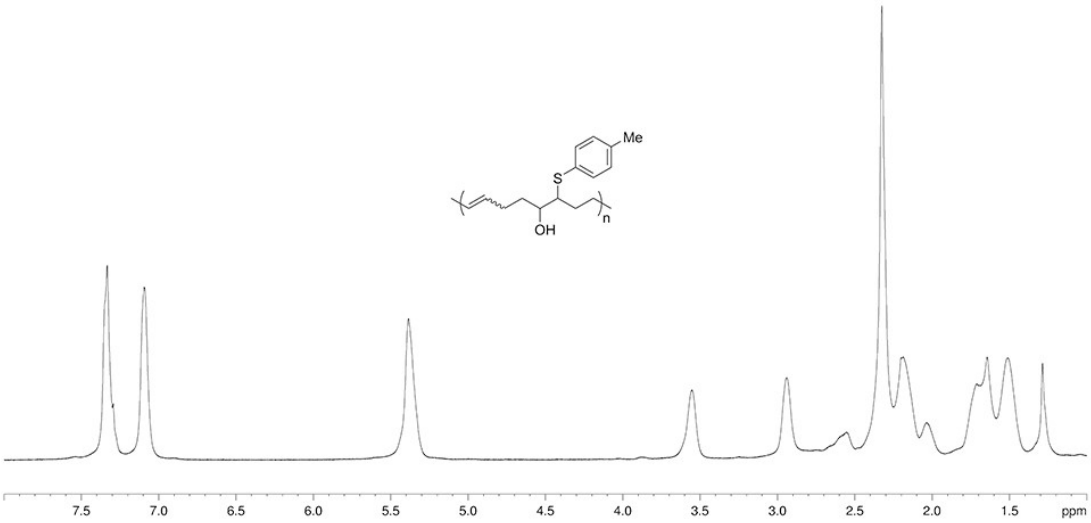

Figure S83. ^{1}H NMR (400 MHz, $CDCl_3$, 25 °C) of Polymer **8a**.

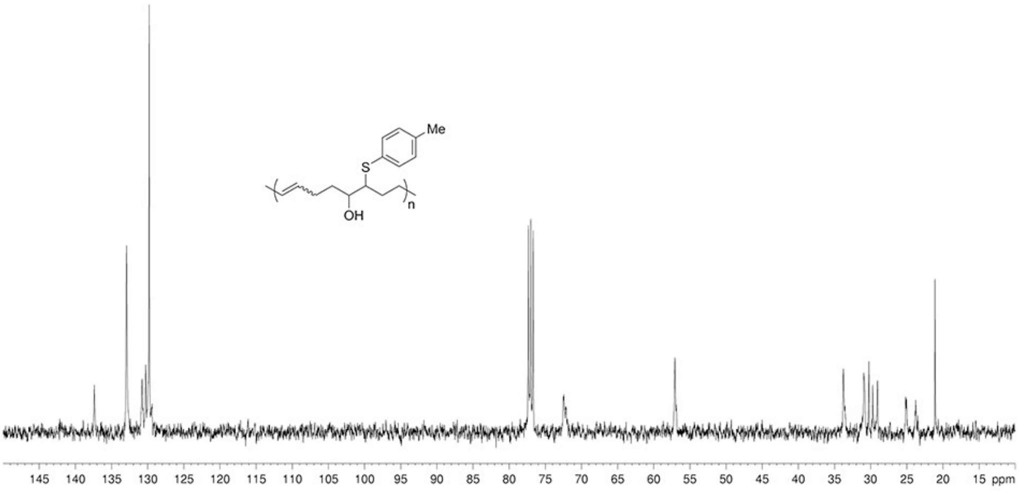

Figure S84. ^{13}C NMR (100 MHz, $CDCl_3$, 25 °C) of Polymer **8a**.

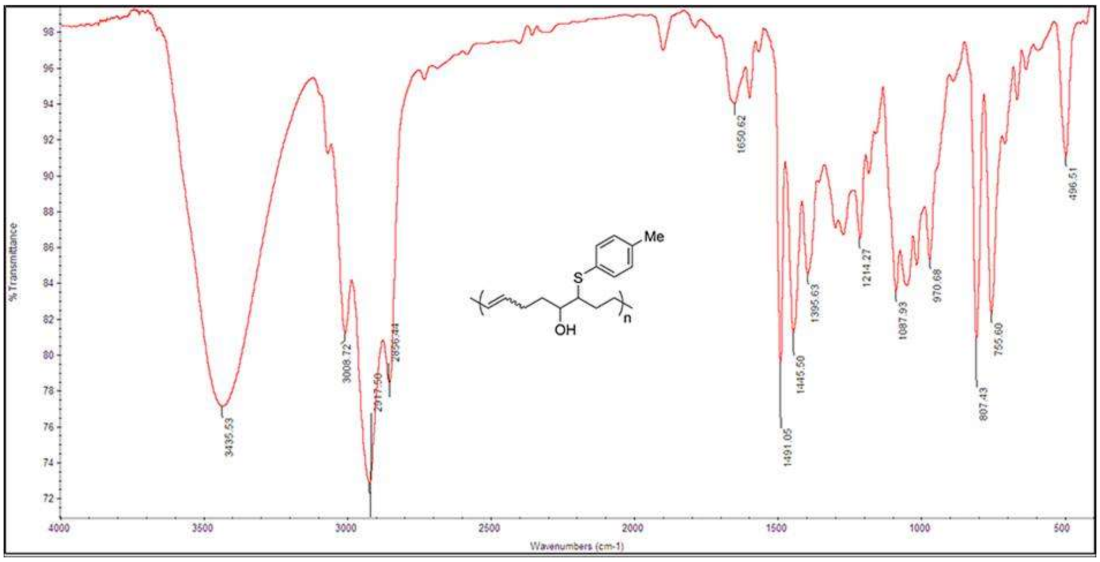

Figure S85. FT-IR spectrum of Polymer **8a**.

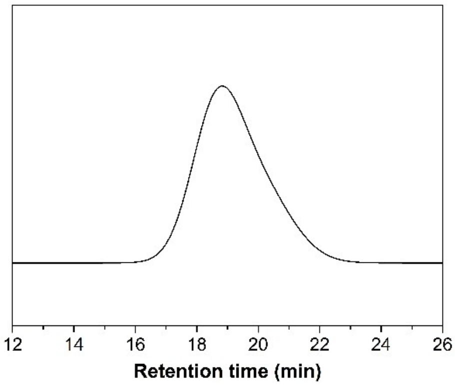

Figure S86. GPC Characterization of Polymer **8a**.

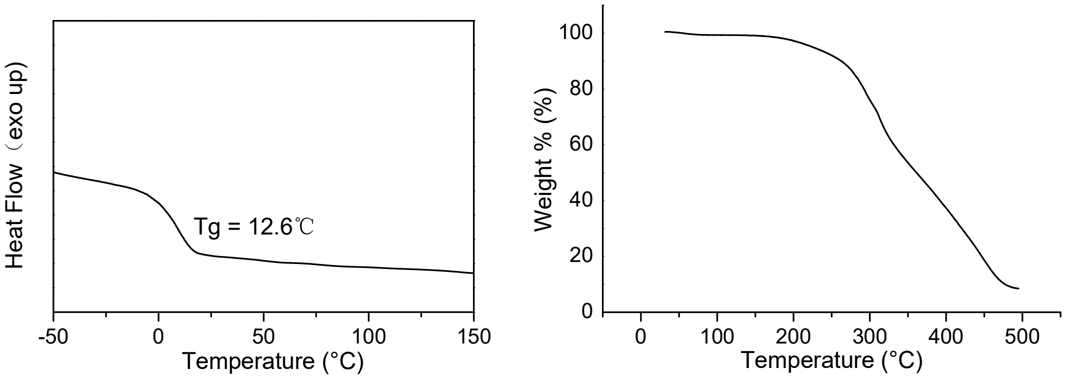

Figure S87. DSC and TGA Characterization of Polymer **8a**.

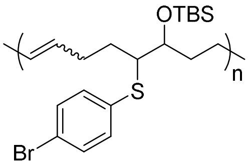

8b ^{1}H NMR (400 MHz, $CDCl_3$) δ: 7.72 – 7.07 (m, 1H), 5.44 (t, 1H), 3.76 (d, 1H), 3.07 (s, 1H), 2.39 – 1.74 (m, 2H), 1.44 (s, 1H), 0.42 – -0.44 (m, 2H). FT-IR (KBr, cm^{-1}): 3363, 2922, 2851, 1659, 1632, 1467, 1411, 1378, 1090, 600, 471.

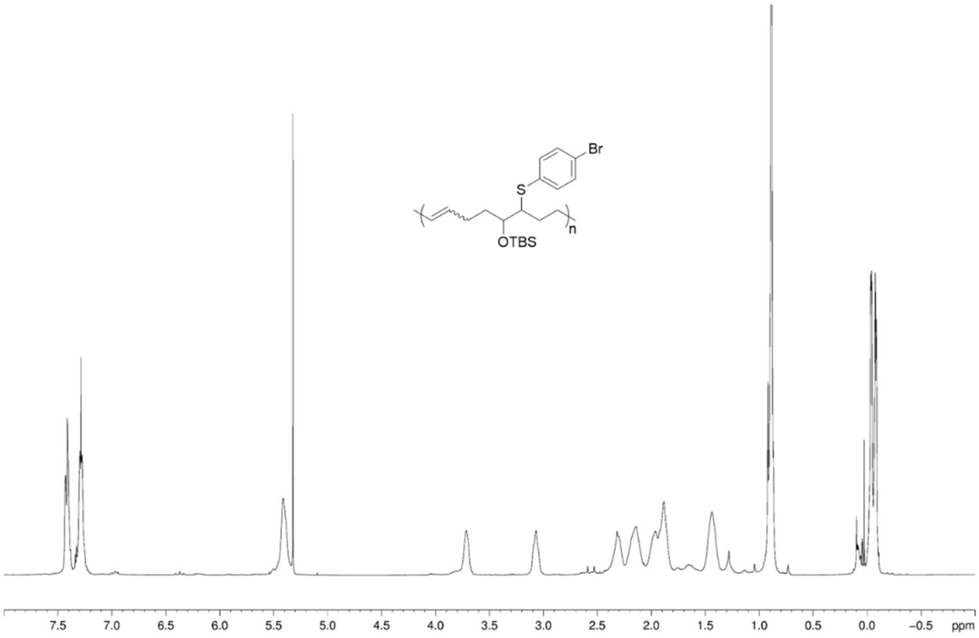

Figure S88. ^{1}H NMR (400 MHz, $CDCl_3$, 25 °C) of Polymer **8b**.

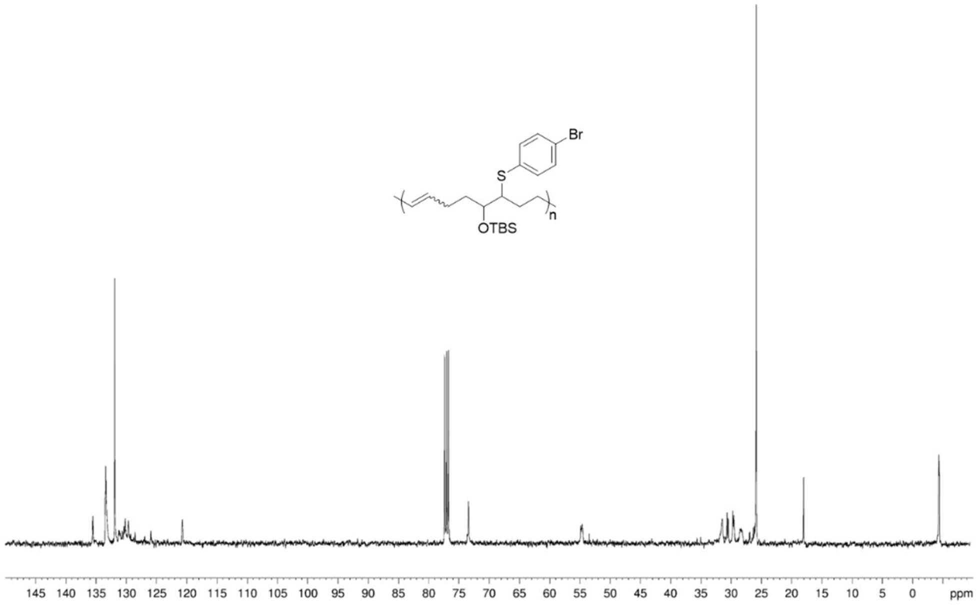

Figure S89.^{13}C NMR (100 MHz, $CDCl_3$, 25 °C) of Polymer **8b**.

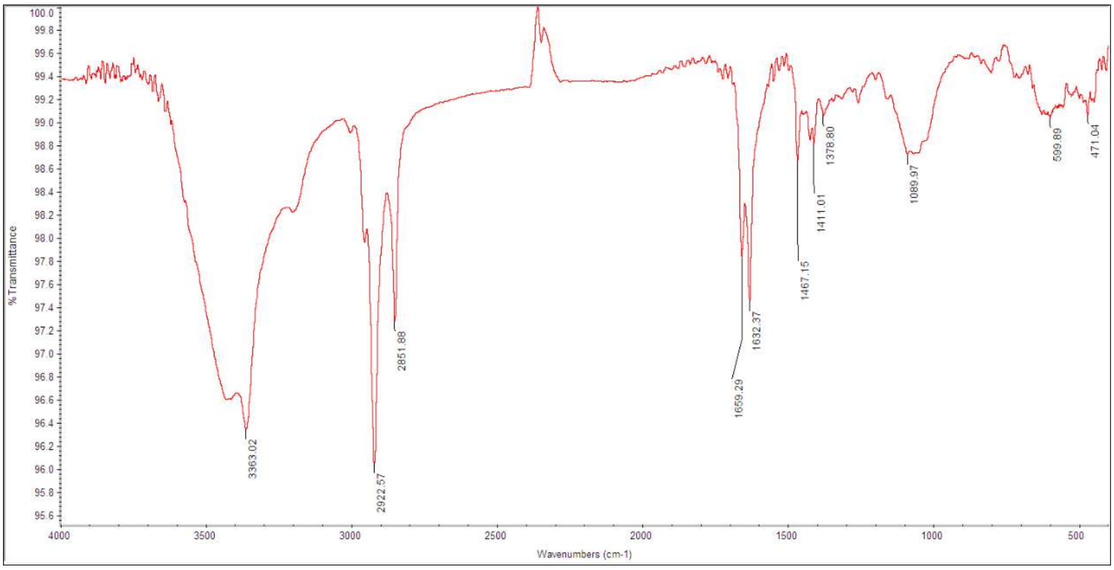

Figure S90. FT-IR spectrum of Polymer **8b**.

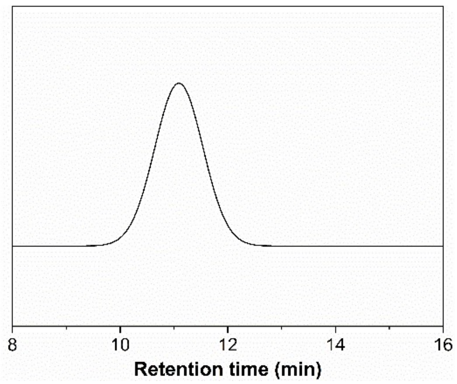

Figure S91. GPC Characterization of Polymer **8b**.

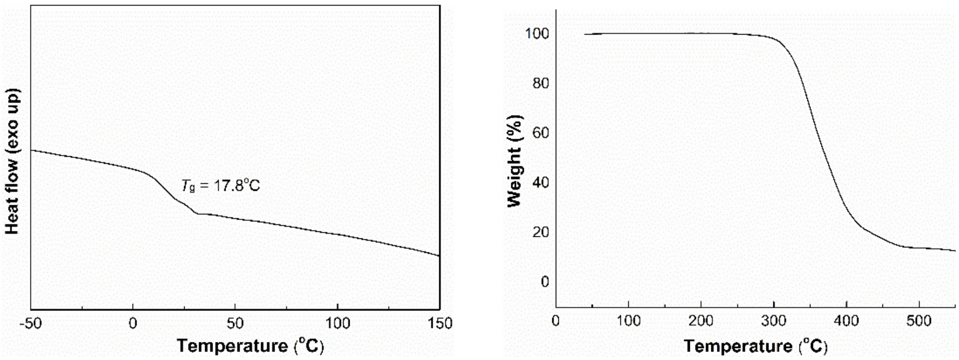

Figure S92. DSC and TGA Characterization of Polymer **8b**.

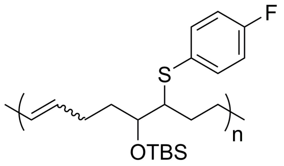

8c ^{1}H NMR (400 MHz, $CDCl_3$) δ:7.50-7.33 (m, 2H), 7.06-6.91 (m,2H), 5.50-5.30 (m, 2H), 3.76-3.61 (m, 1H), 3.06-2.91 (m, 1H), 2.50-2.06 (m, 3H), 2.05-1.80 (m, 3H), 1,53-1.33 (m, 2H), 0.96-0.76 (m, 9H), -0.03--2.10 (m, 6H) ppm; FT-IR (KBr, cm^{-1}) 2948, 2931, 2857, 1590, 1489, 1467, 1227, 1077, 836, 774.

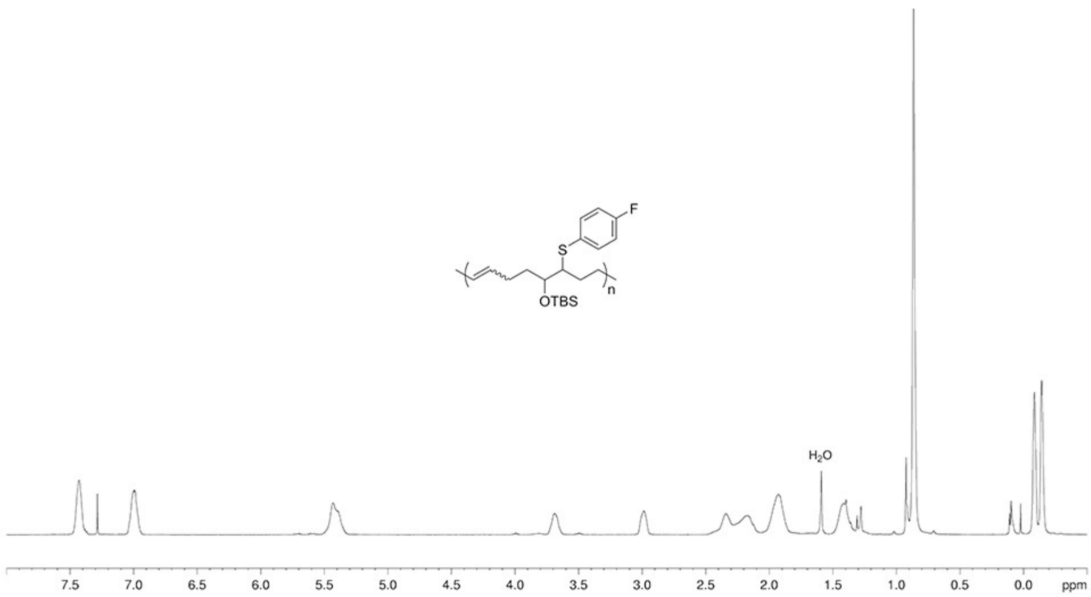

Figure S93. ^{1}H NMR (400 MHz, $CDCl_3$, 25 °C) of Polymer **8c**.

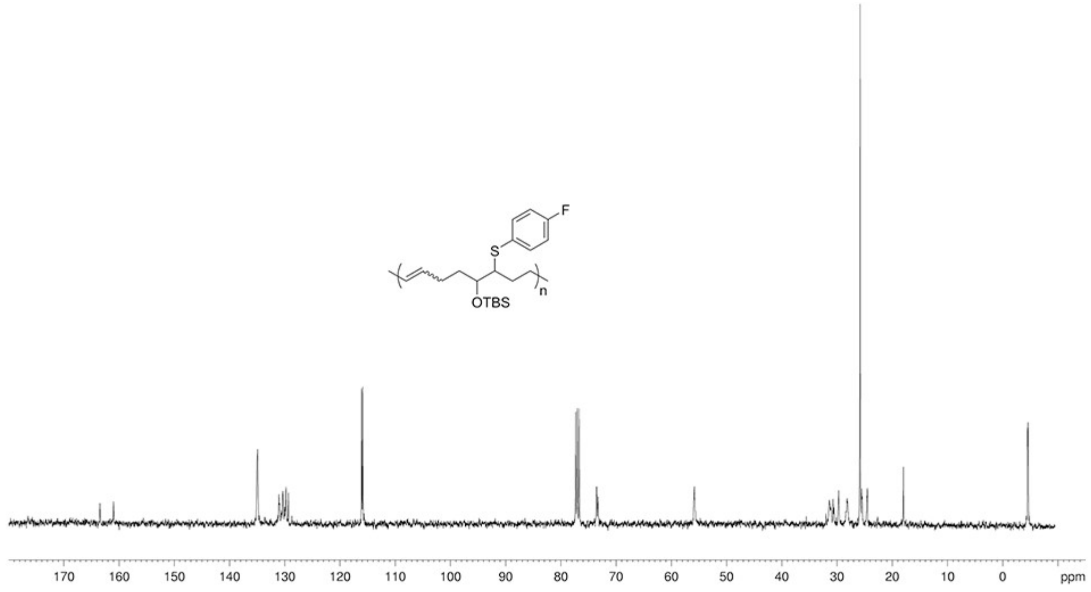

Figure S94. ^{13}C NMR (100 MHz, $CDCl_3$, 25 °C) of Polymer **8c**.

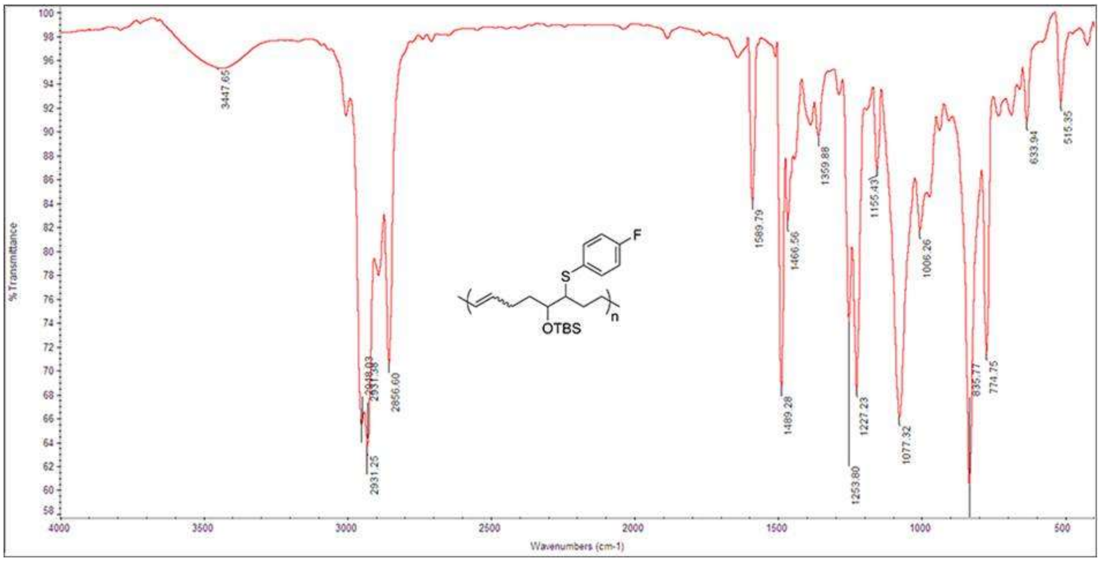

Figure S95. FT-IR spectrum of Polymer **8c**.

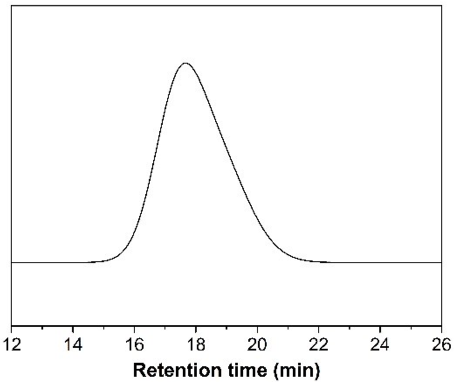

Figure S96. GPC Characterization of Polymer **8c**.

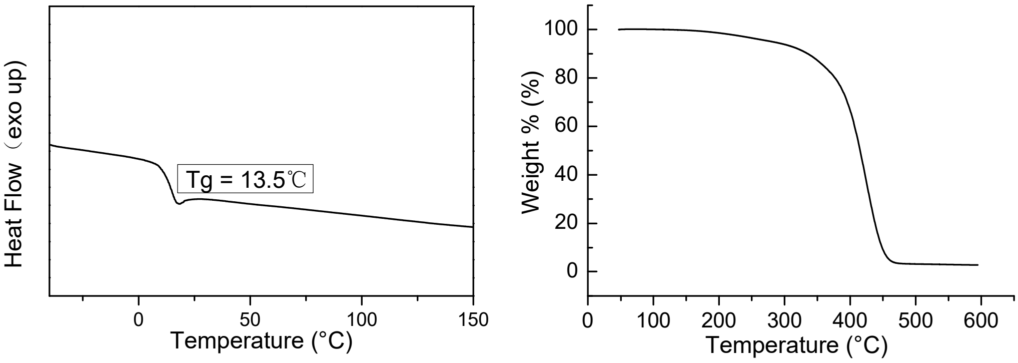

Figure S97. DSC and TGA Characterization of Polymer **8c**.

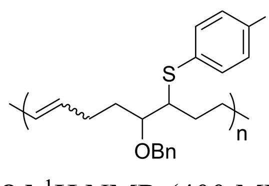

8d ^{1}H NMR (400 MHz, $CDCl_3$) δ: 7.50-7.10 (m, 7H), 7.10-6.88 (m, 2), 5.50-5.16 (m, 2H), 4.52-4.20 (m, 2H), 3.50-3.30 (m, 1H), 3.29-3.07 (m, 1H), 2.49-1.76 (m, 6H), 1.70-1.34 (m, 2H) ppm; FT-IR (KBr, cm^{-1}) 3063, 3029, 2925, 2856, 1588, 1489, 1451, 1225, 1085, 825, 737.

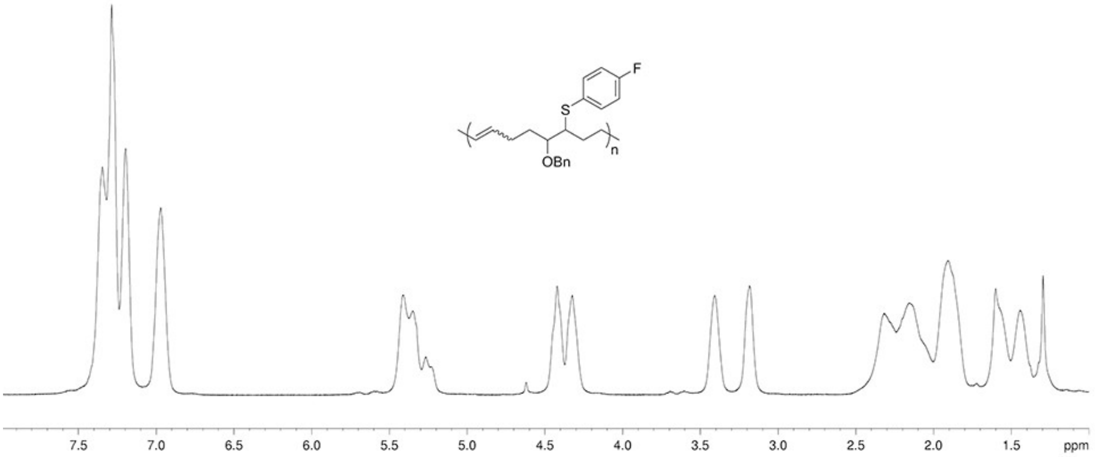

Figure S98. ^{1}H NMR (400 MHz, $CDCl_3$, 25 °C) of Polymer **8d**.

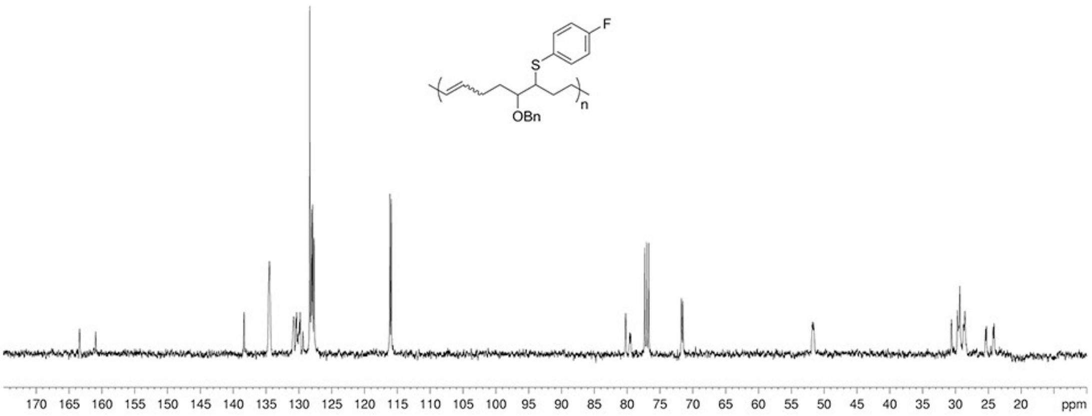

Figure S99. ^{13}C NMR (100 MHz, $CDCl_3$, 25 °C) of Polymer **8d**.

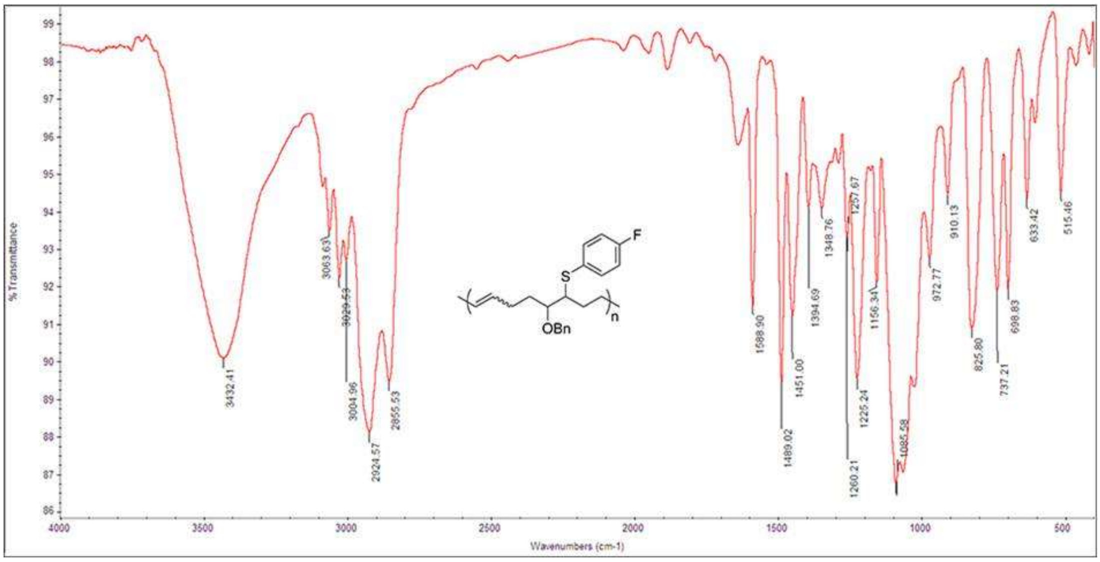

Figure S100. FT-IR spectrum of Polymer **8d**.

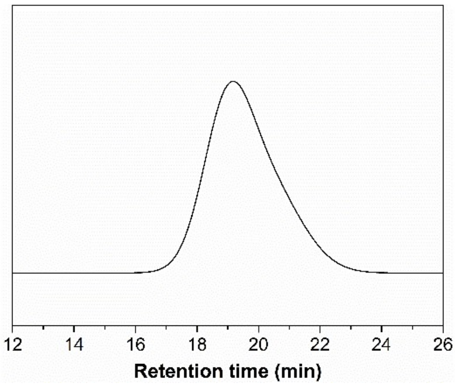

Figure S101. GPC Characterization of Polymer **8d**.

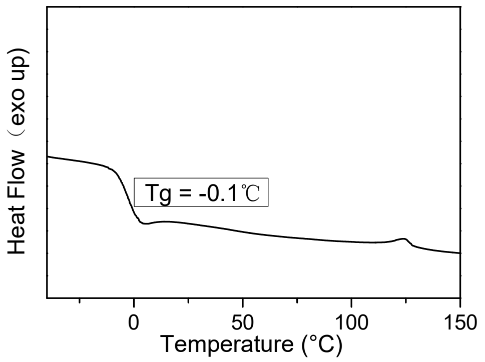

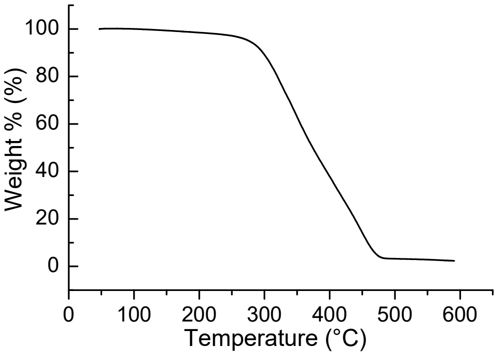

Figure S102. DSC and TGA Characterization of Polymer **8d**.

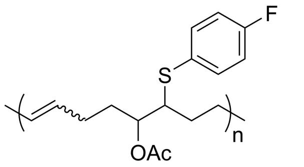

8e ^{1}H NMR (400 MHz, $CDCl_3$) δ: 7.50-7.35 (m, 2H), 7.10-6.90 (m, 2H), 5.44-5.22 (m, 2H), 5.04-4.89 (m, 1H), 3.20-3.05 (m, 1H), 2.36-1.79 (m, 8H), 1.78-1.64 (m, 2H), 1.56-1.37 (m,1H); FT-IR (KBr, cm^{-1}) 2930, 2853, 1737, 1589, 1490, 1443, 1372, 1232, 1021, 829.

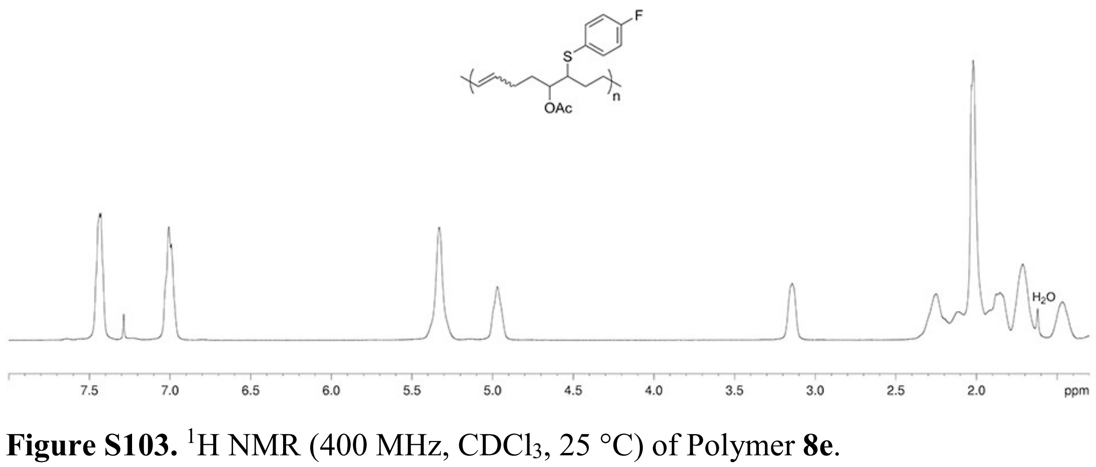

Figure S103. ^{1}H NMR (400 MHz, $CDCl_3$, 25 °C) of Polymer **8e**.

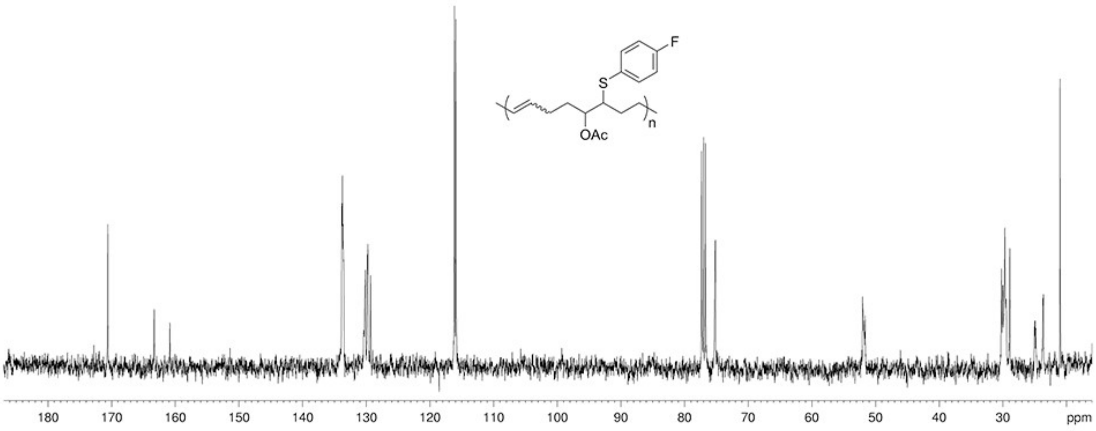

Figure S104. ^{13}C NMR (100 MHz, $CDCl_3$, 25 °C) of Polymer **8e**.

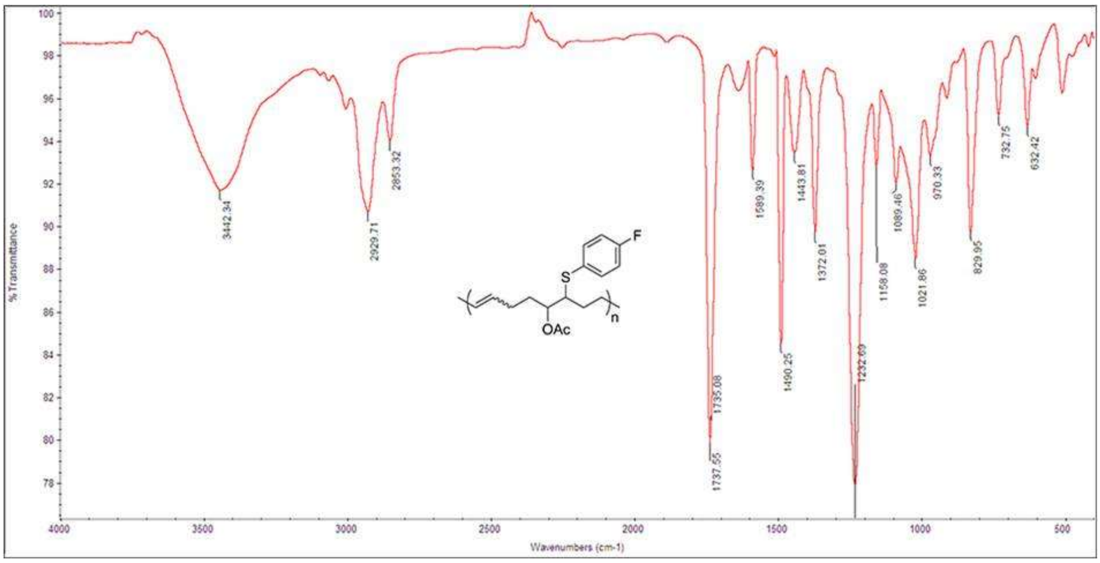

Figure S105. FT-IR spectrum of Polymer **8e**.

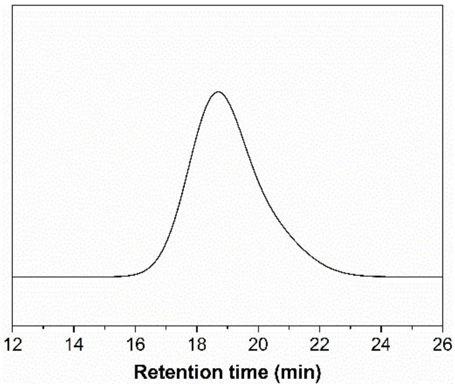

Figure S106. GPC Characterization of Polymer **8e**.

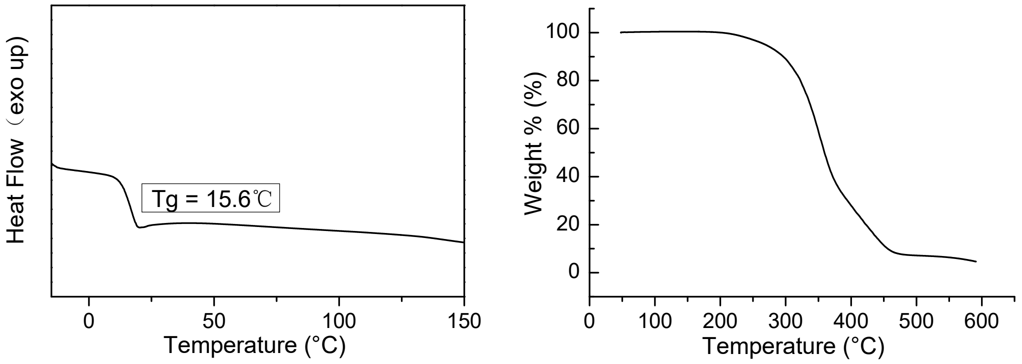

Figure S107. DSC and TGA Characterization of Polymer **8e**.

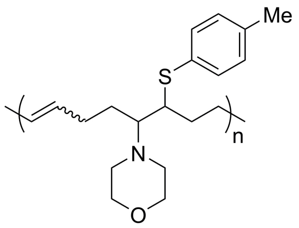

8f ^{1}H NMR (400 MHz, $CDCl_3$) δ: 7.40-7.23 (m, 2H), 7.19-7.01 (m, 2H), 5.52-5.30 (m, 2H), 3.70-3.50 (m, 4H), 3.33-3.15 (m, 1H), 2.70-1.90 (m, 12H), 1.90-1.39 (m, 4H) ppm; FT-IR (KBr, cm^{-1}) 2948, 2851, 1632, 1491, 1449, 1116, 807, 493.

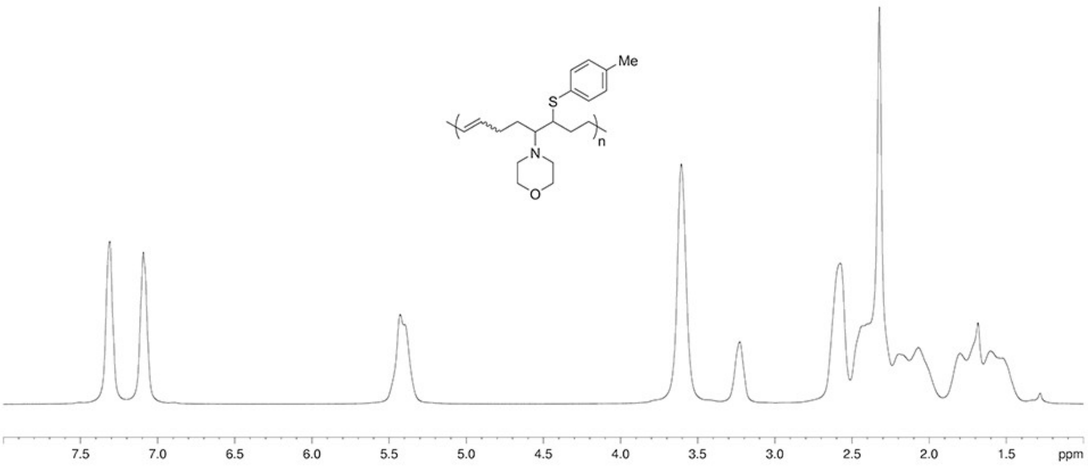

Figure S108. ^{1}H NMR (400 MHz, $CDCl_3$, 25 °C) of Polymer **8f**.

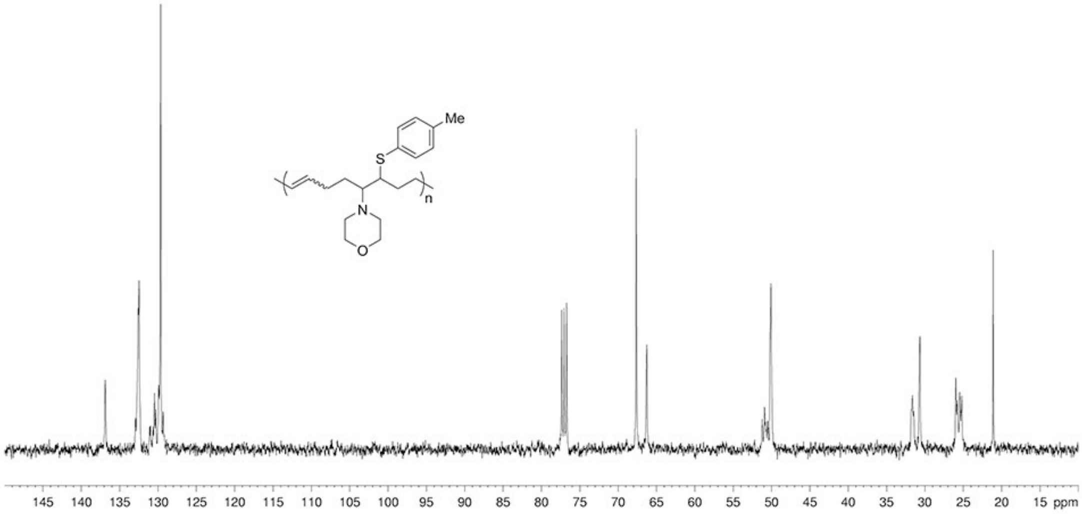

Figure S109. ^{13}C NMR (100 MHz, $CDCl_3$, 25 °C) of Polymer **8f**.

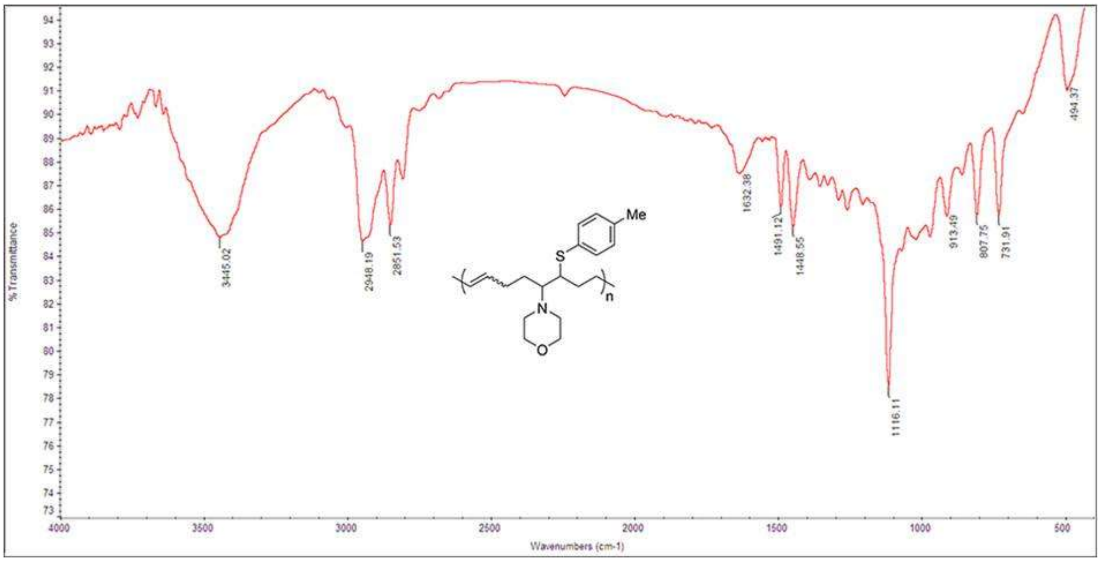

Figure S110. FT-IR spectrum of Polymer **8f**.

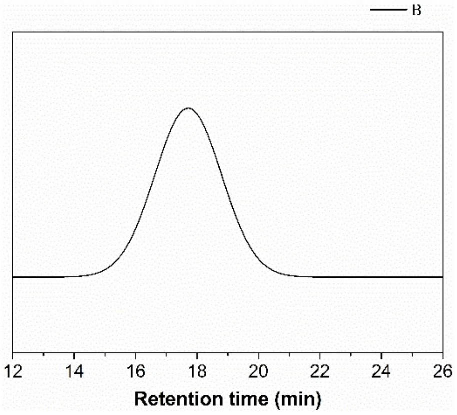

Figure S111. GPC Characterization of Polymer **8f**.

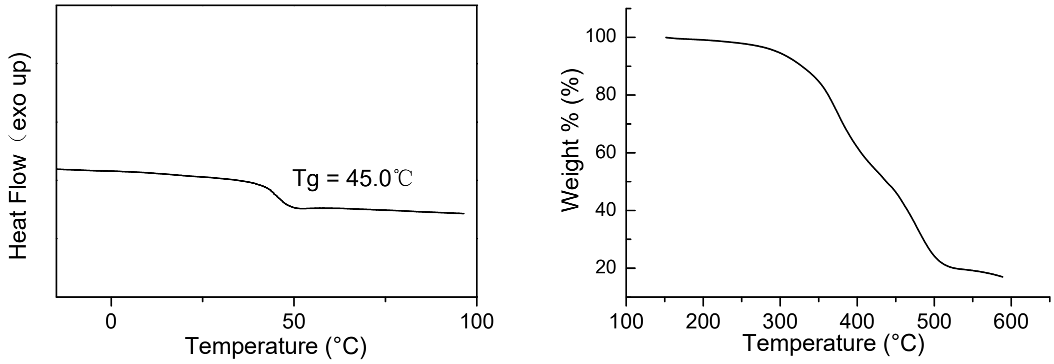

Figure S112. DSC and TGA Characterization of Polymer **8f**.

Section VII:** Synthesis and Characterization of Polymers **9

In a round bottom flask equipped with a stir bar, the unsaturated polymer **6e** (161 mg,

0.53 mmol olefin) was dissolved in 4 mL hot (80°C) *o*-xylene. The solution was cooled to 45°C, and *p*-tosyl hydrazide (494 mg, 2.65 mmol), trihexylamine (716 mg, 2.65 mmol) and 2,6-di-t-butyl-4-methylphenol (BHT, 5 mg, 4%) were added. The mixture was heated at 120 ºC for 10 h. After reaction, the mixture was cooled to room temperature. The solution was washed with water (20 mL×3) and the mixture was concentrated and was dropwisely added into MeOH with vigorously stirring. Solid compound was collected and re-dissolved in minimal amount of DCM. The precipitation procedure was repeated for three times in total to afford target product **9** in 88% isolated yields.

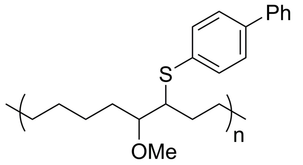

9 ^{1}H NMR (400 MHz, CDCl3) δ: 7.58-7.52 (m, 2H), 7.51-7.36 (m, 6H), 7.35-7.27 (m, 1H), 3.35-3.18 (m, 5H), 1.86-1.54 (m, 4H) ppm, 1.51-1.18 (m, 8H) ppm; FT-IR (KBr, cm^{-1}) 2928, 2854, 1478, 1091, 759.

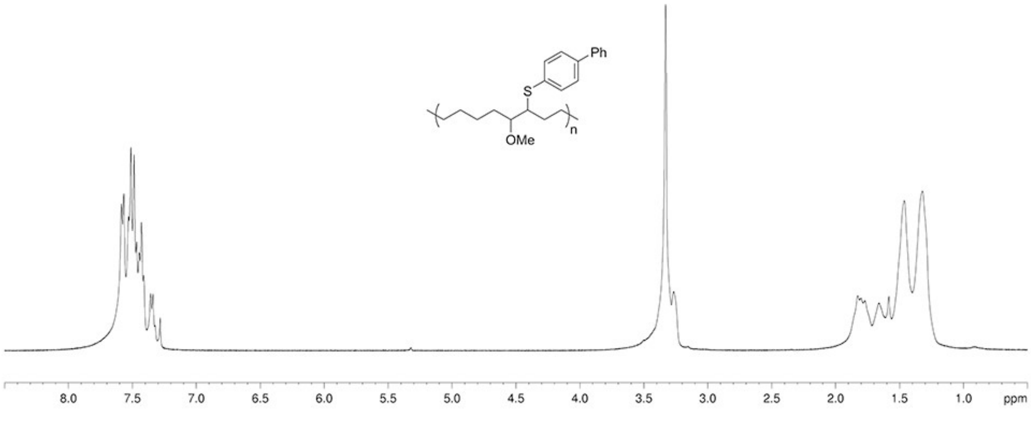

Figure S113 ^{1}H NMR (400 MHz, $CDCl_3$, 25 °C) of Polymer **9**.

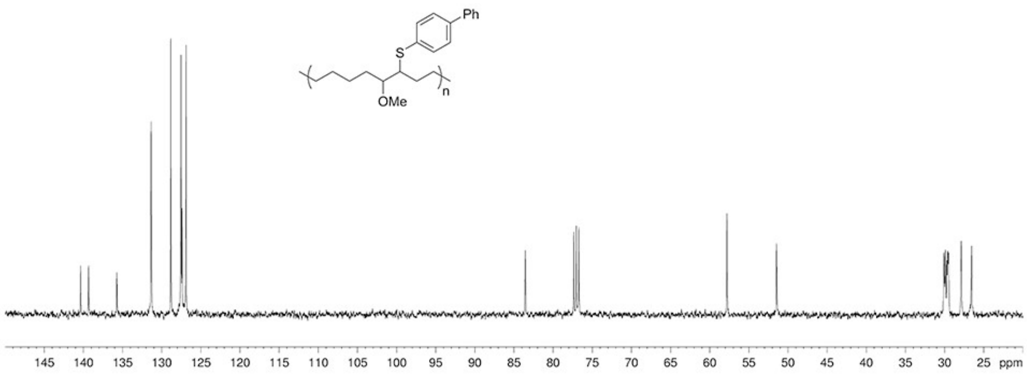

Figure S114. ^{13}C NMR (100 MHz, $CDCl_3$, 25 °C) of Polymer **9**.

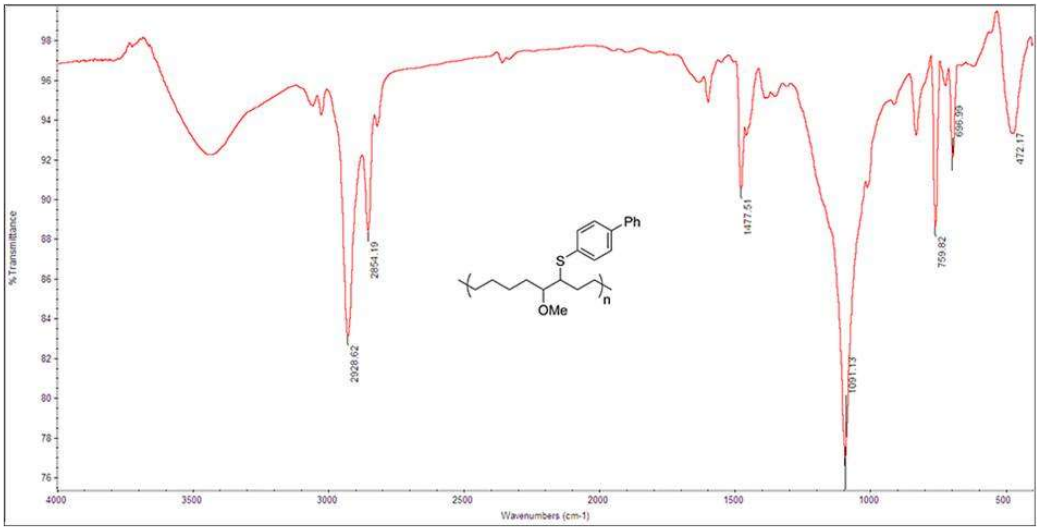

Figure S115. FT-IR spectrum of Polymer **9**.

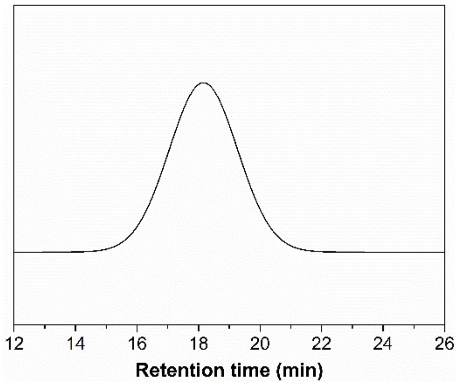

Figure S116. GPC Characterization of Polymer **9**.

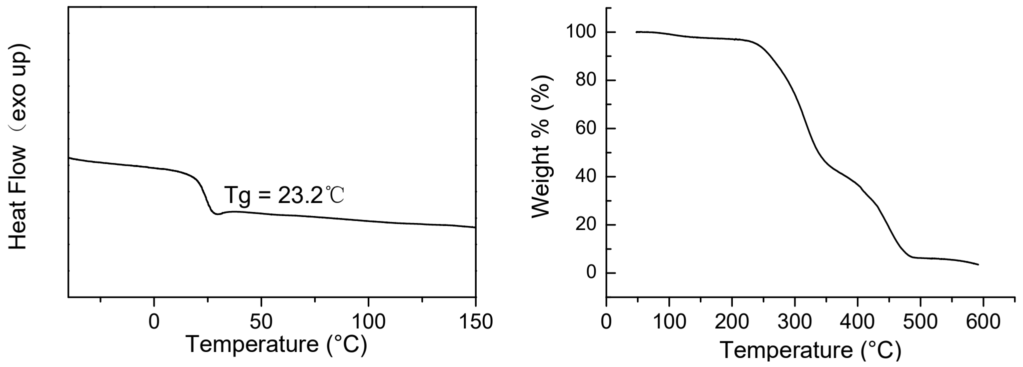

Figure S117. DSC and TGA Characterization of Polymer **9**.

Section VIII: *X-ray Characterization of **7f***

Crystals of compound 7e suitable for X-ray analysis were obtained by slow evaporation from *n*-hexane. Crystallographic data have been deposited with the Cambridge Crystallographic Data Centre (CCDC # 1562950). Copies of the data can be obtained free of charge through application to the CCDC, 12 Union Road, Cambridge CB21EZ, UK (fax: (+44)-1223-336-033; e-mail: deposit@ccdc.cam.ac.uk).

Table S2. Crystal data and structure refinement for **7e**.

Empirical formula	C_{19} $H_{27}NOS$	
Formula weight	317.47	
Temperature	293(2) K	
Wavelength	0.71073 Å	
Crystal system	Orthorhombic	
Space group	P b c a	
Unit cell dimensions	a = 13.4779(14) Å	a = 90°.
	b = 8.1983(9) Å	b = 90°.
	c = 31.894(3) Å	g = 90°.
Volume	3524.1(7) Å^3	
Z	8	
Density (calculated)	1.197 Mg/m^3	

Absorption coefficient	0.186 mm^{-1}
F(000)	1376
Crystal size	0.200 x 0.170 x 0.130 mm^3
Theta range for data collection	1.978 to 25.498°.
Index ranges	-16<=h<=14, -9<=k<=9, -38<=l<=38
Reflections collected	18890
Independent reflections	3265 [R(int) = 0.0361]
Completeness to theta = 25.242°	99.9 %
Absorption correction	Semi-empirical from equivalents
Max. and min. transmission	0.7456 and 0.6769
Refinement method	Full-matrix least-squares on F^2
Data / restraints / parameters	3265 / 0 / 200
Goodness-of-fit on F^2	1.048
Final R indices [I>2sigma(I)]	R1 = 0.0466, wR2 = 0.1143
R indices (all data)	R1 = 0.0563, wR2 = 0.1210
Extinction coefficient	n/a
Largest diff. peak and hole	0.342 and -0.151 e.$Å^{-3}$

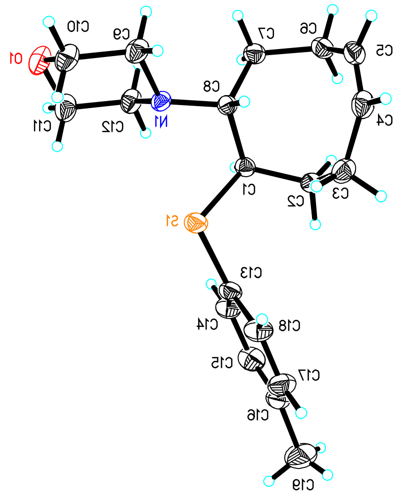

Figure S118. ORTEP diagram of **7f**.

Table S3. Atomic coordinates (x 10^4) and equivalent isotropic displacement parameters (Å^{2}x 10^3) for (7e). U(eq) is defined as one third of the trace of the orthogonalized U^{ij} tensor.

	x	y	z	U(eq)
S(1)	10003(1)	1946(1)	6634(1)	46(1)
N(1)	10374(1)	2148(2)	5777(1)	35(1)
O(1)	11655(1)	41(2)	5330(1)	58(1)
C(1)	9034(1)	2738(2)	6278(1)	35(1)
C(2)	8388(1)	4026(2)	6487(1)	41(1)
C(3)	8855(2)	5698(2)	6546(1)	50(1)
C(4)	8920(2)	6828(2)	6177(1)	50(1)
C(5)	8646(2)	6634(2)	5783(1)	46(1)
C(6)	8187(1)	5162(2)	5582(1)	44(1)
C(7)	8902(1)	3730(2)	5518(1)	40(1)
C(8)	9594(1)	3319(2)	5886(1)	33(1)
C(9)	11139(1)	2786(2)	5503(1)	47(1)
C(10)	11984(1)	1589(3)	5474(1)	54(1)
C(11)	10906(2)	-566(3)	5599(1)	58(1)
C(12)	10035(1)	578(2)	5624(1)	45(1)
C(13)	9337(1)	1717(2)	7111(1)	40(1)
C(14)	8624(1)	530(3)	7162(1)	50(1)
C(15)	8152(2)	331(3)	7542(1)	59(1)
C(16)	8388(2)	1288(3)	7884(1)	57(1)
C(17)	9100(2)	2472(3)	7830(1)	60(1)
C(18)	9562(2)	2696(3)	7449(1)	53(1)
C(19)	7901(2)	1011(4)	8306(1)	93(1)

Table S4. Bond lengths [Å] and angles [°] for **7f**.

S(1)-C(13)	1.7761(18)
S(1)-C(1)	1.8489(16)
N(1)-C(9)	1.449(2)
N(1)-C(12)	1.450(2)
N(1)-C(8)	1.466(2)
O(1)-C(11)	1.415(2)
O(1)-C(10)	1.421(3)
C(1)-C(2)	1.524(2)
C(1)-C(8)	1.535(2)
C(1)-H(1)	0.9800
C(2)-C(3)	1.519(3)
C(2)-H(2A)	0.9700
C(2)-H(2B)	0.9700
C(3)-C(4)	1.500(3)
C(3)-H(3A)	0.9700
C(3)-H(3B)	0.9700
C(4)-C(5)	1.319(3)
C(4)-H(4)	0.9300
C(5)-C(6)	1.501(3)
C(5)-H(5)	0.9300
C(6)-C(7)	1.533(3)
C(6)-H(6A)	0.9700
C(6)-H(6B)	0.9700
C(7)-C(8)	1.536(2)
C(7)-H(7A)	0.9700
C(7)-H(7B)	0.9700
C(8)-H(8)	0.9800
C(9)-C(10)	1.506(3)
C(9)-H(9A)	0.9700
C(9)-H(9B)	0.9700
C(10)-H(10A)	0.9700
C(10)-H(10B)	0.9700
C(11)-C(12)	1.505(3)
C(11)-H(11A)	0.9700

C(11)-H(11B)	0.9700
C(12)-H(12A)	0.9700
C(12)-H(12B)	0.9700
C(13)-C(18)	1.377(3)
C(13)-C(14)	1.377(3)
C(14)-C(15)	1.379(3)
C(14)-H(14)	0.9300
C(15)-C(16)	1.380(3)
C(15)-H(15)	0.9300
C(16)-C(17)	1.376(3)
C(16)-C(19)	1.513(3)
C(17)-C(18)	1.378(3)
C(17)-H(17)	0.9300
C(18)-H(18)	0.9300
C(19)-H(19A)	0.9600
C(19)-H(19B)	0.9600
C(19)-H(19C)	0.9600
C(13)-S(1)-C(1)	101.94(8)
C(9)-N(1)-C(12)	109.97(14)
C(9)-N(1)-C(8)	114.66(14)
C(12)-N(1)-C(8)	115.82(14)
C(11)-O(1)-C(10)	109.90(15)
C(2)-C(1)-C(8)	114.99(14)
C(2)-C(1)-S(1)	112.13(12)
C(8)-C(1)-S(1)	105.21(11)
C(2)-C(1)-H(1)	108.1
C(8)-C(1)-H(1)	108.1
S(1)-C(1)-H(1)	108.1
C(3)-C(2)-C(1)	116.28(15)
C(3)-C(2)-H(2A)	108.2
C(1)-C(2)-H(2A)	108.2
C(3)-C(2)-H(2B)	108.2
C(1)-C(2)-H(2B)	108.2
H(2A)-C(2)-H(2B)	107.4
C(4)-C(3)-C(2)	119.07(16)
C(4)-C(3)-H(3A)	107.5
C(2)-C(3)-H(3A)	107.6
C(4)-C(3)-H(3B)	107.5

C(2)-C(3)-H(3B)	107.6
H(3A)-C(3)-H(3B)	107.0
C(5)-C(4)-C(3)	131.01(18)
C(5)-C(4)-H(4)	114.5
C(3)-C(4)-H(4)	114.5
C(4)-C(5)-C(6)	128.30(18)
C(4)-C(5)-H(5)	115.8
C(6)-C(5)-H(5)	115.8
C(5)-C(6)-C(7)	114.38(15)
C(5)-C(6)-H(6A)	108.7
C(7)-C(6)-H(6A)	108.7
C(5)-C(6)-H(6B)	108.7
C(7)-C(6)-H(6B)	108.7
H(6A)-C(6)-H(6B)	107.6
C(6)-C(7)-C(8)	116.66(14)
C(6)-C(7)-H(7A)	108.1
C(8)-C(7)-H(7A)	108.1
C(6)-C(7)-H(7B)	108.1
C(8)-C(7)-H(7B)	108.1
H(7A)-C(7)-H(7B)	107.3
N(1)-C(8)-C(1)	110.03(13)
N(1)-C(8)-C(7)	113.39(13)
C(1)-C(8)-C(7)	113.03(14)
N(1)-C(8)-H(8)	106.6
C(1)-C(8)-H(8)	106.6
C(7)-C(8)-H(8)	106.6
N(1)-C(9)-C(10)	109.84(16)
N(1)-C(9)-H(9A)	109.7
C(10)-C(9)-H(9A)	109.7
N(1)-C(9)-H(9B)	109.7
C(10)-C(9)-H(9B)	109.7
H(9A)-C(9)-H(9B)	108.2
O(1)-C(10)-C(9)	111.43(16)
O(1)-C(10)-H(10A)	109.3
C(9)-C(10)-H(10A)	109.3
O(1)-C(10)-H(10B)	109.3
C(9)-C(10)-H(10B)	109.3
H(10A)-C(10)-H(10B)	108.0

O(1)-C(11)-C(12)	111.59(17)
O(1)-C(11)-H(11A)	109.3
C(12)-C(11)-H(11A)	109.3
O(1)-C(11)-H(11B)	109.3
C(12)-C(11)-H(11B)	109.3
H(11A)-C(11)-H(11B)	108.0
N(1)-C(12)-C(11)	108.93(16)
N(1)-C(12)-H(12A)	109.9
C(11)-C(12)-H(12A)	109.9
N(1)-C(12)-H(12B)	109.9
C(11)-C(12)-H(12B)	109.9
H(12A)-C(12)-H(12B)	108.3
C(18)-C(13)-C(14)	118.25(17)
C(18)-C(13)-S(1)	119.79(15)
C(14)-C(13)-S(1)	121.89(15)
C(13)-C(14)-C(15)	120.63(19)
C(13)-C(14)-H(14)	119.7
C(15)-C(14)-H(14)	119.7
C(14)-C(15)-C(16)	121.4(2)
C(14)-C(15)-H(15)	119.3
C(16)-C(15)-H(15)	119.3
C(17)-C(16)-C(15)	117.59(18)
C(17)-C(16)-C(19)	121.3(2)
C(15)-C(16)-C(19)	121.1(2)
C(16)-C(17)-C(18)	121.3(2)
C(16)-C(17)-H(17)	119.3
C(18)-C(17)-H(17)	119.3
C(13)-C(18)-C(17)	120.8(2)
C(13)-C(18)-H(18)	119.6
C(17)-C(18)-H(18)	119.6
C(16)-C(19)-H(19A)	109.5
C(16)-C(19)-H(19B)	109.5
H(19A)-C(19)-H(19B)	109.5
C(16)-C(19)-H(19C)	109.5
H(19A)-C(19)-H(19C)	109.5
H(19B)-C(19)-H(19C)	109.5

Symmetry transformations used to generate equivalent atoms:

Table S5. Anisotropic displacement parameters ($Å^2$x 10^3) for **7f**. The anisotropic displacement factor exponent takes the form: $-2p^2[h^2 a^{*2}U^{11} + ... + 2 h k a^* b^* U^{12}]$

	U^{11}	U^{22}	U^{33}	U^{23}	U^{13}	U^{12}
S(1)	36(1)	64(1)	38(1)	11(1)	1(1)	7(1)
N(1)	35(1)	34(1)	34(1)	0(1)	6(1)	-2(1)
O(1)	52(1)	54(1)	67(1)	-11(1)	19(1)	5(1)
C(1)	31(1)	39(1)	34(1)	3(1)	-1(1)	0(1)
C(2)	40(1)	47(1)	35(1)	5(1)	6(1)	5(1)
C(3)	58(1)	50(1)	43(1)	-10(1)	3(1)	1(1)
C(4)	52(1)	35(1)	62(1)	-6(1)	7(1)	-2(1)
C(5)	51(1)	36(1)	53(1)	7(1)	6(1)	4(1)
C(6)	42(1)	46(1)	43(1)	8(1)	-4(1)	3(1)
C(7)	45(1)	40(1)	36(1)	1(1)	-2(1)	0(1)
C(8)	37(1)	31(1)	32(1)	0(1)	1(1)	-1(1)
C(9)	44(1)	44(1)	52(1)	0(1)	14(1)	-5(1)
C(10)	40(1)	62(1)	60(1)	-4(1)	14(1)	-2(1)
C(11)	58(1)	41(1)	74(1)	-2(1)	16(1)	5(1)
C(12)	44(1)	37(1)	55(1)	-5(1)	9(1)	-3(1)
C(13)	36(1)	51(1)	34(1)	8(1)	-5(1)	2(1)
C(14)	49(1)	56(1)	44(1)	4(1)	-6(1)	-6(1)
C(15)	45(1)	70(2)	62(1)	18(1)	3(1)	-8(1)
C(16)	51(1)	77(2)	43(1)	16(1)	6(1)	17(1)
C(17)	66(1)	76(2)	38(1)	-4(1)	-5(1)	4(1)
C(18)	52(1)	63(1)	44(1)	3(1)	-7(1)	-10(1)
C(19)	91(2)	131(3)	58(2)	21(2)	28(1)	16(2)

Table S6. Hydrogen coordinates (x 10^4) and isotropic displacement parameters ($Å^2$x 10^3) for **7f**.

	x	y	z	U(eq)
H(1)	8607	1824	6196	42
H(2A)	7789	4155	6322	49
H(2B)	8192	3621	6761	49
H(3A)	8487	6257	6764	60
H(3B)	9523	5535	6650	60
H(4)	9202	7838	6236	60
H(5)	8752	7524	5608	56
H(6A)	7919	5477	5311	53
H(6B)	7637	4799	5754	53
H(7A)	9310	3962	5275	49
H(7B)	8513	2767	5454	49
H(8)	9930	4336	5963	40
H(9A)	11380	3817	5612	56
H(9B)	10866	2977	5226	56
H(10A)	12483	2009	5284	65
H(10B)	12288	1467	5748	65
H(11A)	11180	-719	5878	69
H(11B)	10684	-1619	5498	69
H(12A)	9740	703	5348	54
H(12B)	9536	138	5811	54
H(14)	8460	-144	6938	60
H(15)	7664	-465	7569	71
H(17)	9272	3134	8055	72
H(18)	10033	3518	7419	63
H(19A)	8017	1939	8483	140
H(19B)	8178	55	8434	140
H(19C)	7201	864	8268	140

Table S7. Torsion angles [°] for **7f**.

C(13)-S(1)-C(1)-C(2)	42.38(14)
C(13)-S(1)-C(1)-C(8)	168.06(12)
C(8)-C(1)-C(2)-C(3)	-45.3(2)
S(1)-C(1)-C(2)-C(3)	74.83(17)
C(1)-C(2)-C(3)-C(4)	77.9(2)
C(2)-C(3)-C(4)-C(5)	-1.2(3)
C(3)-C(4)-C(5)-C(6)	-1.0(4)
C(4)-C(5)-C(6)-C(7)	-72.6(3)
C(5)-C(6)-C(7)-C(8)	43.9(2)
C(9)-N(1)-C(8)-C(1)	-161.42(14)
C(12)-N(1)-C(8)-C(1)	68.81(18)
C(9)-N(1)-C(8)-C(7)	70.9(2)
C(12)-N(1)-C(8)-C(7)	-58.88(19)
C(2)-C(1)-C(8)-N(1)	165.31(14)
S(1)-C(1)-C(8)-N(1)	41.43(16)
C(2)-C(1)-C(8)-C(7)	-66.80(19)
S(1)-C(1)-C(8)-C(7)	169.31(12)
C(6)-C(7)-C(8)-N(1)	-169.39(15)
C(6)-C(7)-C(8)-C(1)	64.5(2)
C(12)-N(1)-C(9)-C(10)	-57.4(2)
C(8)-N(1)-C(9)-C(10)	170.01(15)
C(11)-O(1)-C(10)-C(9)	-57.4(2)
N(1)-C(9)-C(10)-O(1)	57.2(2)
C(10)-O(1)-C(11)-C(12)	58.7(2)
C(9)-N(1)-C(12)-C(11)	57.9(2)
C(8)-N(1)-C(12)-C(11)	-170.06(15)
O(1)-C(11)-C(12)-N(1)	-59.1(2)
C(1)-S(1)-C(13)-C(18)	-112.85(16)
C(1)-S(1)-C(13)-C(14)	70.23(17)
C(18)-C(13)-C(14)-C(15)	0.0(3)
S(1)-C(13)-C(14)-C(15)	176.92(16)
C(13)-C(14)-C(15)-C(16)	-1.2(3)
C(14)-C(15)-C(16)-C(17)	1.2(3)
C(14)-C(15)-C(16)-C(19)	-177.1(2)
C(15)-C(16)-C(17)-C(18)	-0.1(3)
C(19)-C(16)-C(17)-C(18)	178.2(2)

C(14)-C(13)-C(18)-C(17)	1.2(3)
S(1)-C(13)-C(18)-C(17)	-175.85(16)
C(16)-C(17)-C(18)-C(13)	-1.1(3)

Symmetry transformations used to generate equivalent atoms:
